# Ethical aspects of the use of social robots in caring for older people – a systematic qualitative review

**DOI:** 10.1007/s11019-025-10313-3

**Published:** 2026-02-05

**Authors:** Marianne Leineweber, Clara Victoria Keusgen, Marc Bubeck, Robert Ranisch, Joschka Haltaufderheide, Corinna Klingler

**Affiliations:** Junior Professorship for Medical Ethics with Focus on Digitization, Faculty of Health Sciences Brandenburg, University of Potsdam, Am Mühlenberg 9, 14476 Potsdam, Germany

**Keywords:** Social robots, Socially assistive robots, Health services for the aged, Dementia, Ethics, Systematic review

## Abstract

The use of social robotics in care for older persons is increasingly discussed as one way of meeting emerging care needs due to scarce resources. While many potential benefits are associated with robotic care technologies, there is a variety of ethical challenges. To support steps towards a responsible implementation and use, this review develops an overview on ethical aspects of the use of social robots in care for older people from a decision-makers’ perspective. Electronic databases were queried using a comprehensive search strategy based on the key concepts of “ethical aspects”, “social robotics” and “elderly care”. Abstract and title screening was conducted by two authors independently. Full-text screening was conducted by one author following a joint consolidation phase. Data was extracted using MAXQDA24 by one author, based on a consolidated coding framework. Analysis was performed through modified qualitative content analysis. A total of 1,518 publications were screened, and 248 publications were included. We have organized our analysis in a scheme of ethical hazards, ethical opportunities and unsettled questions, identifying at least 60 broad ethical aspects affecting three different stakeholder groups. While some ethical issues are well-known and broadly discussed our analysis shows a plethora of potentially relevant aspects, often only marginally recognized, that are worthy of consideration from a practical perspective. The findings highlight the need for a contextual and detailed evaluation of implementation scenarios. To make use of the vast knowledge of the ethical discourse, we hypothesize that decision-makers need to understand the specific nature of this discourse to be able to engage in careful ethical deliberation.

**Supplementary Information:**

The online version contains supplementary material available at 10.1007/s11019-025-10313-3.

## Background

Demographic change and the persistent care worker shortage have prompted discussion on the use of assistive robotic technologies as potential solutions for expected gaps in care for older people (Berner et al. 2020; Haltaufderheide et al. 2023a). As a result, extensive efforts are being made in the area of research, research funding and development of robotic technologies (Wright 2024). The industry is more and more investing in the development of care robots (Buxbaum and Sen 2018). In some countries like Japan, where a high percentage of the population comprises older citizens, the government already provides subsidies to care facilities for the purchase of robots (Savage 2022), shifting from a developmental to an implementation and use focus. While it is fair to assume that some hopes and expectations regarding robotic technologies are exaggerated and warrant critical reflection, it is widely acknowledged that technological support through robots will assume an increasingly important role in care.

Robotic systems designed to support care work can be grouped roughly into two overlapping categories of (physically) assistive and socially assistive robots (Maalouf et al. 2018; Matarić and Scassellati 2016). While the former support tasks such as bringing, carrying, lifting activities of the caregivers or physical activities of the care recipients, the latter facilitate social interaction. Systems of that kind are known as social robots, socially assistive robots or companion robots. They can support social interaction^1^ or even interact with older adults themselves (Kehl 2018a), thereby simulating emotions or use natural language^2^. As such they gain access to particularly sensitive areas of human life. For that reason, the use of social robotics in care for older adults is considered particularly ethically sensitive, while also providing novel opportunities to connect older people and improve their well-being (Kachouie et al. 2014).

While there are many potential benefits associated with the use of social robotics, there are also a variety of ethical challenges discussed in the literature (Mordoch et al. 2013; Vandemeulebroucke et al. 2018; Boada et al. 2021): Advocates often emphasize that the use of robotics may mitigate upcoming resource shortages by relieving caregivers, especially in particularly time-intensive or physically demanding tasks (Haltaufderheide et al. 2023a; Sharkey and Sharkey 2012). More cautious perspectives, on the other hand, argue that the “human touch of care” may be lost and care relationships thereby impoverished (Kehl 2018b; Sparrow and Sparrow 2006) or that robots cannot consider the individually varying needs of older people (Remmers 2020). In addition, questions of autonomy and dependency (Boada et al. 2021), dignity of caring relationships (Felber et al. 2022), adequate data protection (Felzmann et al. 2016), as well as issues related to deceptions (Sharkey and Sharkey 2021) are frequently discussed.

Consequently, the research discourse notes a variety of ethical challenges and opportunities that might vary depending on the type of robot, implementation scenario and broader sociotechnical arrangement, as well as the ethical perspective (Vandemeulebroucke et al. 2018). Given that it is to be expected that social robots will become a part of nursing care practices for older people, decision-makers on the micro-, meso- and macro-level will have to consider and navigate these ethical issues arising. While this topic is critically discussed in the literature (Haltaufderheide et al. 2020; Hung et al. 2022; Wachsmuth 2018) and increasingly addressed by national ethics bodies (Bioethikkommission beim Bundeskanzleramt Österreich 2019; Deutscher Ethikrat 2020), no pertinent standards for the use of social robots in care settings have been developed. For both the development of guidelines and individual decision-making, knowing about relevant ethical aspects arising in this context and being able to engage with the existing ethical knowledge will be paramount. Against this background, this systematic review aims to provide a broad overview of relevant ethical aspects concerning the use of social robotics in care for older people as discussed in the literature following the question: What are the ethical aspects of using social robots in care for older adults? In the following, we will outline the methods for realizing the review, including the search, the screening process and the analysis of the data. Then, we will present our results regarding our research question and discuss their implications.

## Methods

The review is reported following the RESERVE guideline for systematic reviews in ethics (Kahrass et al. 2023). A protocol was agreed upon by the authors and has been registered with PROSPERO (Klingler et al. 2023). Unlike other reviews on this topic that primarily focus on ethical challenges (Boada et al. 2021) or specific aspects and concepts (Vandemeulebroucke et al. 2018), our aim was to include all relevant aspects – including the opportunities or benefits of technologies – deemed significant for ethical decision-making. The goal of this work is thereby purely descriptive. It does not evaluate or weigh up the aspects discussed in the literature as no accepted criteria or procedures for synthesizing normative data have been developed (Klingler and Mertz 2021). We assume that decision-makers will need to take further evaluative steps and apply arguments raised to their context as discussed below. Nevertheless, this overview serves as a valuable resource for decision-makers at both local and policy levels, providing a clear synthesis of the ethical aspects that should be considered.

### Search strategy

The search strategy was developed in collaboration with an information specialist from the Potsdam University Library. Preliminary searches were made to determine feasibility and overlap of inclusions. As Mathew et al. (2024) argue, an adequate search strategy is not determined by the absolute number of databases but by whether producers and users of the review can be reasonably assured that the likelihood of missing relevant studies is negligible. In line with this reasoning, we assumed that relevant discussions would take place within the ethical, nursing, medical, and technical literature. Our search strategy was, thus, tailored to the interdisciplinary and ethics-oriented nature of the topic: alongside major health and nursing databases (PubMed/Medline, CINAHL) and a technical database (TIB-Portal), we also included BELIT, a specialized database for ethics in the life sciences, as well as grey literature (policy briefs, ethics council statements) available in the TIB-Portal and BELIT. While additional databases could have been consulted, the incremental yield for this particular research question is likely limited, thus, our strategy balances comprehensiveness with efficiency.

Search strings were constructed around the three core concepts: (a) ethical aspects, (b) social robotics, and (c) elderly care^3^. In doing so, we followed Droste et al. (2010), who argue that the PICO framework is unsuitable for ethics-related topics. Instead, we narrowed the scope by specifying the ethical dimension, the intervention, and the user group or care setting. The search string development was informed by a preliminary search and literature screening to identify relevant terminology. The search string for PubMed is presented in Table 1.

**Table 1:** Search string used in PubMed/Medline

Concept	Corresponding part of search string
(a) ethical aspects	(ethics[MeSH Terms] OR human rights[MeSH Terms] OR ethic*[Text Word] OR moral*[Text Word]) AND
(b) social robotics	(robotics[MeSH Terms] OR robot*[Text Word] OR (social*[Text Word] AND assistiv*[Text Word]) OR (social*[Text Word] AND interactiv*[Text Word])) AND
(c) elderly care	(aged[MeSH Terms] OR aged[Text Word] OR geriatr*[Text Word] OR elder*[Text Word] OR senior* [Text Word] OR nursing home*[Text Word] OR dement*[Text Word])

Search strings for the other databases were modeled after the PubMED-string (see Online Resource 4) and adjusted for database-specific features such as language or literature type. The search was conducted in February and March of 2023.

### Inclusion/exclusion criteria

As at least two terms used in the research question are contested and only vaguely specified in the literature, we developed operationalizable criteria for the purpose of this review. We understand “social robots” as robots whose primary purpose is to either support social interaction as a mediator (socially assistive robots) or serve as an actual interaction partner (socially interactive robots) – a definition employed by Kehl (2018a), building on the work of Feil-Seifer and Matarić (2005).

In specifying what constitutes a robot, we are guided by Fosch-Villaronga and Drukarch (2022), who define a “robot” as a “movable machine that performs tasks either automatically or with degree of autonomy”. A “machine” is operationalized as having a physical body capable of interacting with its environment. This definition was deemed appropriate as it allows us to exclude neighboring technologies that fall outside the scope of this review, such as exoskeletons, cleaning robots, or virtual avatars. To determine whether this criterion was met, respective device descriptions were checked using included literature and – where necessary – further sources describing them in more detail.

Terms like “ethical issues” or “ethical aspects” are also inconsistently used in the literature (Schofield et al. 2021). We used the latter to refer to hazards, opportunities or unsettled questions that need to be considered (or clarified) when determining how to responsibly handle a given phenomenon. To operationalize the term “ethical aspects”, we employed a principle-based approach that presumes certain ethical principles as relevant orientation points in the field of robotics/AI: autonomy, beneficence, nonmaleficence, justice and explicability (Beauchamp and Childress 2019; Floridi et al. 2018).

We defined ethical aspects as instances where at least one of the five principles is promoted (ethical opportunities), violated (ethical hazards), or where there is ambiguity or a conflict between different ethical claims or principles (unsettled questions). By choosing such a substantive approach, we reduced the risk of overlooking relevant literature that does not explicitly use normative terminology to describe ethical aspects. Further context-related criteria (e.g., publications had to self-identify as discussing elderly care, as we did not want to introduce an age cutoff) and formal inclusion criteria (to guarantee a certain quality of publications and due to language limitations) were defined upfront.^4^

### Screening procedures

Two researchers independently screened title and abstract of identified literature independently using Colandr (Cheng et al. 2018). During the title/abstract screening, we adopted an inclusive approach, including all papers that addressed ethical aspects of using robots in elderly care, even if we were uncertain whether social robotics were specifically discussed. To manage the significant quantity of findings, collected volumes were not included in their entirety in the full-text screening; instead, only potentially relevant book chapters were considered. One researcher (ML) screened the title, table of contents, and summary descriptions of identified books to determine relevant chapters.

Access to full texts was sought via various libraries, and authors of relevant publications were contacted directly when necessary. We were able to retrieve 295 publications for full-text screening – a number significantly higher than expected. Accordingly, after a consolidation phase, it was decided that the full-text screening would be conducted by only one reviewer (either ML or CVK), in parallel with analysis. The first 25 publications were screened jointly by at least two reviewers, one of whom (CK) has extensive experience in conducting systematic reviews in ethics. All decisions were discussed extensively, and any uncertainties arising during the full-text screening were resolved through further discussion with CK.

### Quality appraisal

As outlined by Mertz (2019), there are many open questions regarding how to conduct quality appraisal in systematic reviews that address normative questions. Due to the lack of an appropriate methodology, we have decided to not conduct a quality appraisal as part of our review. However, we attempted to ensure a baseline level of quality for the included literature by defining formal inclusion criteria (e.g., focus on academic publications and policy documents).

### Data extraction

Based on our research question and context of interest we did not conduct data extraction in a standardized, quantitative format. Instead, a qualitative document analysis was conducted using MAXQDA24, as described below. However, we did extract certain information from the papers to be able to describe our sample, such as bibliographic information (see Online Resource 2), the disciplinary affiliation of the first author, and the type of articles. Visualizations of the sample based on those dimensions have been made available as supplemental online material (see Online Resource 3).

### Data analysis and synthesis

The data was analyzed and synthesized using qualitative content analysis adapted from Kuckartz (2018) and Schreier (2012). Synthesis in this context refers to the systematic process of reducing, structuring, and integrating heterogeneous findings into a coherent coding frame that captures recurring patterns across studies. We deemed an inductive qualitative approach as outlined above especially suitable for this task as it presents a structured method to develop higher abstraction layers based on the available material through a process of reduction, reorganization and subsumption. First, a purposively selected sample – maximum variation sampling (Patton 2001) – of 10 publications was analyzed independently by two authors (CK and ML). Categories describing the material were developed inductively using the strategies of summarizing and subsumption, as outlined by Schreier (2012). The resulting coding frame as the first step of our synthesis was discussed between the two authors, and a consolidated framework was developed. This framework was used to analyze a second purposively selected sample of 10 papers.

This step was conducted by three reviewers independently (CK, CVK, and ML). The resulting preliminary coding frame was discussed with the rest of the team to ensure its validity, consistency, and clarity of the synthesized categories. Once robustness of the coding frame and a shared understanding among the authors of this paper were established (by an additional joint analysis of five papers by CK and CVK), the remaining literature was analyzed by only one reviewer (ML or CVK). The two reviewers met regularly with CK to discuss new codes or uncertainties that arose during analysis.

After the first 100 publications, further works were only used to validate the framework. Relevant text passages were checked for representation in the coding frame but were only explicitly coded if they discussed an ethical aspect not already included in the framework or where they constituted a particularly illustrative example quote.

## Results

**Fig. 1:**
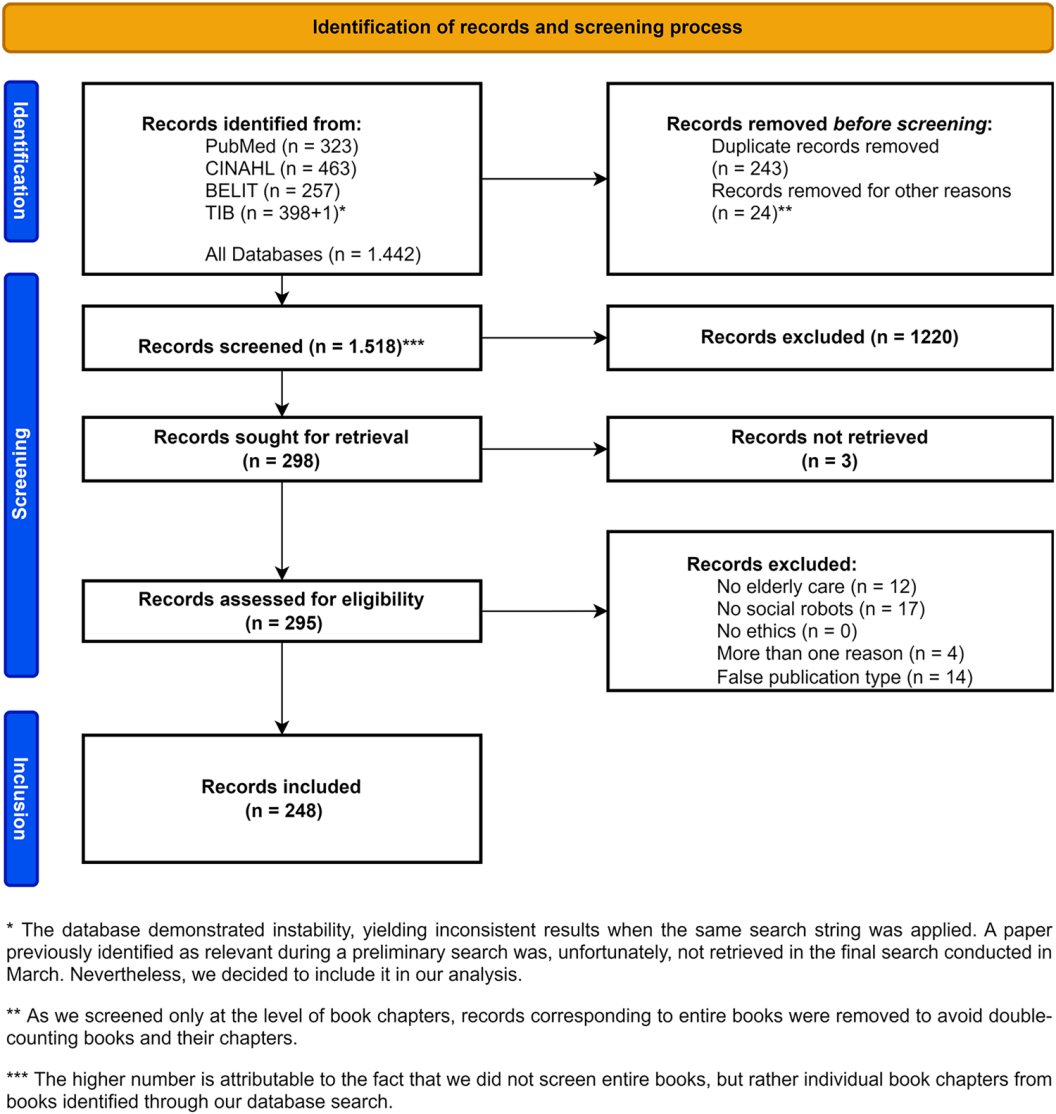
PRISMA 2020 Flow diagram of identification of studies via databases

Our literature search identified 1.442 publications across the mentioned four databases. After the removal of duplicates and identification of potentially relevant book chapters, 1.518 publications remained for title-abstract screening. During this process, 1.220 publications were excluded. The remaining 298 publications were sought for retrieval, and all but three publications could be obtained. Therefore 295 publications were included in full-text screening. In this phase, 47 publications were excluded due to different reasons (see Fig. 1 for full information on screening process), leaving 248 publications for analysis (see bibliographic information of included publications in Online Resource 2).

### Hazards and opportunities

In the following, we use a framework of ethical opportunities and hazards to structure our reporting. This framework acknowledges that the integration of technologies often brings various benefits but also involves trade-offs, sometimes resembling a “Faustian Bargain” as Neil Postman (1992) called it, where advancements provide new possibilities while simultaneously taking away or compromising certain values or goods. Ethical opportunities and hazards are categorized according to their impact on three primary stakeholder groups: care recipients, care providers/facilities (including informal carers), and society and the healthcare system. This approach allows for a balanced examination of how these technologies contribute to the realization of goods while also highlighting their potential risks or costs.

For each main category (e.g., ethical hazards/opportunities) we identified subcategories, which we differentiated up to the third level (decreasing in abstraction) to make visible the wide variety of perspectives and potential usages of social robotics. Only the first-level subcategories are presented in the paper, but an overview of all subcategories, including example quotes for transparency, is available as supplemental material (see Online Resource 1). At the first level, we identified 60 subcategories, with 141 subcategories on the second level and 123 on the third. Most categories on either level pertain to the care recipient group.

Besides these categories, our analysis revealed a number of unsettled questions which were highlighted as ethically salient but could not be clearly categorized within the framework of ethical opportunities and hazards. These include, for example, ethical arguments pointing to conflicts between different stakeholder groups, potential issues affecting all stakeholders in divergent ways, or aspects which were conceptually, normatively or empirically too ambiguous to be classified as a hazard or an opportunity. These aspects are, therefore, presented separately. We, thus, understand these questions to present issues which either require a context-specific judgement or to be based on unclear, unsettled or ambiguous normative or empirical claims which require further investigation or debate to be decided.

In the following, we will provide a short narrative overview of our findings. As the ethical aspects encountered in the literature are extensive, a more in-depth introduction of the diverse aspects will only be exemplary. For a detailed overview, consult Online Resource 1.

#### For care recipients

The ethical opportunities and hazards for care recipients primarily relate to the ethical dimensions of *well-being and health*, *privacy*, *discrimination*, *independency*, *informed decision-making*, *social connection*, *adaptation of care to individual needs*, *efficiency*, *deception*,* trust*, and the *impact of lacking regulation* as captured in the respective first-level categories denoting a positive or negative impact (see Table 2 for a full overview of first-level categories). It is argued that engagement in relations with social robots may positively impact perceptions of *quality of life*, various health parameters such as positive physiological reactions to the interaction (Draper and Sorrell 2017) or increased bodily or social activity as well as a positive effects on the overall *well-being* (Jecker 2021). In addition, it is hypothesized that older people’s health and wellbeing could benefit from safety measures provided by some devices (e.g., fall protection, oversight of daily household tasks) (Bioethikkommission beim Bundeskanzleramt Österreich 2019). However, caution is warranted with regard to a satisfaction of emotional and, in some cases, physical needs, which some authors suggest may only be superficially “satisficed” rather than fully satisfied in a broader and ethically significant sense (Frennert and Östlund 2014; Misselhorn 2018). As, for example, Remmers (2019) argues, caregivers might withdraw from care due to the availability of technical alternatives leading to a loss of relationship work that characterizes good care.

*“All these developments are associated with benefits such as physical and cognitive relief for care staff*,* but also risks such as the loss of experience-saturated judgment and relationship work based on this*,* among other things. [translation by the authors]” (*Remmers 2019, 413).

**Table 2:** Ethical opportunities and hazards for care recipients in the use of social robots in care for older people

Main category	Subcategory (first-level)
Ethical opportunities for care recipients	Improving/ensuring well-being/health
	Restoring privacy
	Promoting positive perception/preventing stigmatization/discrimination of care recipient
	Providing support in daily living (esp. where capacities reduced)
	Sustaining independent and established lifestyle
	Furthering social connection
	Supports tailoring care approaches to needs/taste
	Support/care provided (more) efficiently
Ethical hazards for care recipients	Reducing/not adequately securing well-being/health
	Infringing the right to privacy
	Promoting discrimination/negative images of care recipient
	Increasing dependency in everyday life
	Creating barriers for autonomous and informed decision-making
	Increasing social isolation
	Care not tailored to varying needs
	Deception about relationships/personal bonds that do not exist
	Loss of trust in carers (including social robot carer)
	Beneficial social robots not/no longer put to use
	Lack of protection due to lack of guidelines/legal regulations

In addition, unauthentic social relations might let users get accustomed to a reduced range of emotional interactions machines can offer (Diaz-Orueta et al. 2020), thereby impoverishing their lifeworld (Boada et al. 2021).

Aspects of *discrimination* and *biases* are frequently discussed. Regarding the design and appearance of devices it is noted that interacting with robots that exhibit certain features (e.g., toy-like or child-like designs) may risk infantilizing or stigmatizing users (Frennert and Östlund 2014). In addition, some authors raise concerns that robots might reproduce and further stereotypic images of older people as care-dependent or may lead to infantilization of care recipients (Servaty et al. 2020). Fosch-Villaronga and Virk (2017) raise the possibility of care robots discriminating against certain groups, for example hearing or speech impaired persons who are not able to participate in interactions with devices based on acoustic interfaces. With respect to the interaction of social robots with care recipients, it has, however, been positively highlighted that the consistent behavior of these devices – regardless of the age, race, or gender of care recipients – may help circumvent implicit or explicit biases in caring practices (Weßel et al. 2022).

*Quality of care* is another dimension subject to both opportunities and hazards. On the one hand, quality of care could improve as robots reduce caregiver workload, thereby mitigating risks of neglect or even abuse stemming from caregiver fatigue (Noori et al. 2019). On the other hand, quality may decline if robots are assigned tasks they are not (yet) capable of performing effectively or were never designed to handle (Zöllick et al. 2020). Aspects of *independency* reflect a similar ambiguity. Social robots may enable care recipients to become less dependent on other people (Haltaufderheide et al. 2020). As Wiertz (2020) argues:

*“The option to rely on assistive technology systems instead of being dependent on human caregivers can thus prima facie be understood as a valuable option of gaining a degree of independence.”* (Wiertz 2020, 53).

However, relying on a robot may simultaneously increase technological dependency (Li et al. 2020), leading to significant challenges, for example, in case of robot malfunctions. Devices that motivate technological dependency, for example, by offering excessive assistance or by motivating delegation of even simple tasks may thereby contribute to a deterioration of habits and skills instead of maintaining them (Li et al. 2020).

Privacy concerns are another major theme. Health data collection and the (digital) transfer of such data by social robots frequently raises privacy concerns (Bendel 2020). However, robots lacking personal involvement or interests of their own may, in some cases, offer an advantage over human caregivers, who, by virtue of their roles, naturally intrude into private contexts, potentially infringing on privacy (Sharkey and Sharkey 2014). Similarly, while robots substituting human caregivers are often criticized for reducing social interaction and exacerbating loneliness, the opposite may hold true in some settings:

*“During a pandemic emergency in particular*,* the alternative to robot companionship for many older people is social isolation and loneliness. Without support*,* older adults are left to languish. Under these conditions*,* sociable robots do not rob older adults of human companionship but afford companionship where it is lacking.”* (Jecker 2021, 39).

On most of these dimensions, the usage of social robotics is seen to potentially produce both hazards and opportunities, where their realization often depends on specific contextual factors. However, some dimensions lean more heavily toward potential hazards, such as debates around *deception* regarding the robot’s nature or the relationship with it (Matarić and Scassellati 2016). While the term deception is sometimes used as a prescriptive term (Danaher 2020), implying that deception inherently constitutes a moral wrong, other sources emphasize that the risk of deception might be particularly pronounced in robots with anthropomorphic or zoomorphic design, especially when interacting with patients suffering from neurocognitive impairments such as dementia.

#### For care providers

At the level of care providers and care facilities the ethical opportunities and hazards can be assigned to the dimensions of *well-being/health*,* privacy*,* working conditions*,* relationships*,* nursing as profession*,* technological literacy*,* care facility management*, and the *impact of lacking regulations* (see first-level categories in Table 3 for a more detailed overview). Similar to the ethical aspects relevant to care recipients, most dimensions present both hazards and opportunities. As the dimensions *well-being/health*,* privacy* and *impact of lacking regulations* overlap with those of the care recipients, the following examples focus on aspects specific to care providers and care facilities.

**Table 3:** Ethical opportunities and hazards for care providers in the use of social robots in care for older people

Main category	Subcategory (first-level)
Ethical opportunities for care providers/facilities (including caring relatives)	Positively impacting care provider’s health and well-being
	Improving working conditions for care providers
	Positively implicates relationships
	Upgrading nursing profession
	Easing management for care facilities
Ethical hazards for care providers/facilities (including caring relatives)	Adverse effect on health/well-being of care providers
	Aggravate work situation for care providers
	Infringing on privacy of care providers
	Negatively implicates relationships
	Feeling insecure in handling social robots
	Complicating management for care facilities
	Lack of protection due to lack of guidelines/legal regulations

One of the main aspects regarding care providers concerns the conditions under which care is delivered. Social robots may mitigate difficult care conditions or alleviate the burden of a high workload in professional care (Wirth et al. 2020). Wahl et al. (2021) argue that:

*“It is well known that professional carers in geriatric care are under more psychological and physical strain than carers in other areas. High time pressure and stress*,* together with the physical consequences of work (especially back pain)*,* lead to high staff turnover. Robotic systems can be used here in various ways to provide support and relieve the burden on caregivers and/or informal or formal caregivers. [Translation by the authors]” *(Wahl et al. 2021, 63).

The use of technological support could, hence, positively affect the health and well-being of care providers. A reduced strain on care workers might have additional benefits as improving the quality of care often means providing better working conditions for caregivers (Salvini 2015). However, some authors caution that a shift in tasks and roles may lead caregivers to become supervisors of their technical aids (Frebel 2015) or face additional tasks (e.g., cleaning the device) (Hung et al. 2019). A more severe risk may lie in the disturbance of human care processes or loss of control of care. As most devices lack awareness of when to interrupt or not to interrupt care interactions (Bioethikkommission beim Bundeskanzleramt Österreich 2019) care workers need to take into account frequent interruptions of potentially sensible processes. In general, as Manzeschke (2022) argues, robotics imply a certain degree of autonomy on the side of the devices which may raise concerns about who is in control of the caring process.

Another dimension that can be positively and negatively impacted by robotic technology is the *relationship* of care recipients and providers. Relationships can be strengthened if robots take on straining tasks, such as assisting with bathroom visits (Borenstein and Pearson 2014). In contrast, the use of social robots could also negatively impact *relationships*, for example, if the care recipient loses confidence in the caregiver because the social robot is used to monitor them (Jenkins and Draper 2015), or if conflicts arise between those involved in care processes over which tasks robots should be allowed to perform (Tan et al. 2021).

A further hazard mentioned is that care providers might feel insecure or stressed when dealing with social robots, particularly if they lack the necessary technological literacy and cannot acquire relevant competencies during work hours (Niemelä et al. 2021).

Interestingly, ethical aspects relating to the care facility and its management are also highlighted, as social robots might ease or complicate their managerial tasks. For the *care facility management*, robots might turn out as a market advantage by supporting the acquisition of personnel and nursing home residents (Bleuler and Caroni 2021), or reducing costs of care (Früh and Gasser 2018). Additionally, they could be used to monitor the performance of care personnel (Draper and Sorell 2017) and can be used flexibly since, unlike human caregivers, robots do not tire or act out (Pirhonen et al. 2020). However, robots might also pose a market disadvantage if they are perceived as an indicator for low quality, potentially deterring prospective residents (Meyer 2011). Other challenges include organizing maintenance and support, especially if the technology is no longer supported, and the high costs associated with training staff to use these devices (Hung et al. 2019).

#### For society and the healthcare system

The ethical opportunities and hazards for society and the (healthcare) system can be categorized into the following dimensions: *ensuring provision of care*, *discrimination*, *social and moral development*,* economy and ecology*, *regulation and design of social robots*, *(under)standing of care/being human*,* justice*,* responsibility*,* efficiency*, and the *“rise of the robots*” (see Table 4 for a complete overview on the first-level-categories).

**Table 4:** Ethical opportunities and hazards for society in the use of social robots in care for older people

Main category	Subcategory (first-level)
Ethical opportunities for society/the (healthcare) system	Supporting state in ensuring comprehensive nursing care for all
	Reducing discrimination/stigmatization of certain groups
	Promoting desired behavior of citizens
	Important field for economic development (location advantage)
Ethical hazards for society/the (healthcare) system	Moving forward without an adequate knowledge/discussion base
	Using a misguided approach to regulation/design of social robots
	Beneficial social robots not used due to diverse barriers
	Problematic change in (under)standing of care
	Problematic change in understanding of being human
	Damaging interpersonal and societal connectedness
	Moral de-skilling
	Fueling discrimination/stigmatization of certain groups
	Unfair distribution of benefits and burdens of robot introduction
	Erosion of responsibility/gaps in the responsibility structure
	Inefficient use of social robots
	Ecologically and socially not sustainable
	Social robots get too much power and endanger humans/human society

One frequently discussed opportunity is that social robots could help to *ensure the provision of care* in the context of demographic change and a nursing shortage by taking on tasks that would otherwise require significant human resource investment (Kehl 2018c). Robots could also contribute to a more just society by being deployed to underserved populations and context, such as rural areas (Bleses and Dammert 2020). Conversely, there is the concern that the *(under)standing of care* in society could change in problematic ways through the use of social robots (Manzeschke 2019). It is argued that there is more to care than an input-output-process and that an important component of caregiving is lost when tasks are performed by robots, which cannot care in the way humans can (they take care, but do not care) (von Maur 2023).

Another prominently discussed hazard is that the *design of social robots* may be driven more by technical feasibility or engineering preferences than by the specific needs of real-world care situation, which often differ significantly from laboratory settings (Paletta et al. 2019). This misalignment could result in robots being underutilized, thereby wasting resources (Boada et al. 2021).

A less frequently raised but interesting argument is that relying on social robots in care could curtail the need for human interaction with vulnerable individuals, reducing opportunities to develop compassion and leading to moral de-skilling. As Boada et al. (2021) state:

*“… the adoption of SARs in care, by outsourcing practices central to human existence to non-human actors, could blind us from the awareness of the constitutive vulnerability and (inter)dependence of human life, thus threatening the cultivation of virtues essential to a flourishing society. More specifically, the new technological practices could reduce the opportunities for cultivating moral skills regarding human caregiving." * (Boada et al. 2021, 7).

While the introduction of social robots has been viewed as a potential hazard to social development, in some areas, their integration may also signal moral progress. For example, the use of sex robots – a type of social robot – has been argued to help break taboos surrounding sexuality in old age (Koumpis and Gees 2020). Similarly, robots designed with gender-critical perspectives could play a role in dismantling traditional gender stereotypes, thereby fostering inclusivity and equality (Weßel et al. 2021).

### Unsettled questions and themes

Unsettled questions in the scientific discourse relate to the *cost-benefit ratio of social robots*,* just allocation/prioritization*,* discrimination*, *privacy*, *deception*, *autonomy and informed decision-making*, *responsibility*, *understanding of care*, and *social robots as autonomous agents.* These issues are highlighted as being worthy of consideration from an ethical perspective but do not clearly classify as either hazard or opportunity or are conceptually, normatively or empirically too ambiguous to be classified as such. Some of the hazards and opportunities mentioned above are revisited here but framed as ethical conflicts, illustrating what is gained and lost through the use of social robots and how certain hazards and opportunities are interconnected. A prominent example is the question of how to ethically appraise the possible *deception* of care recipients given the potential for positive effects on well-being. As mentioned above, particularly cognitively impaired patients can mistake social robots for real humans or animals. At the same time, there are indications in the literature that certain opportunities, like reduced stress (Wada and Shibata 2007) or increased communication between care recipient and relatives (Sharkey and Sharkey 2011) may better be realized where at least some level of deception is accepted. In the literature it remains normatively unresolved whether the deceit is ethically acceptable given the resulting health gains (Kreis 2018).

Further aspects mentioned in this context concern, for example, the question whether privacy risks associated with monitoring and transmitting health data – for instance to ensure well-being through immediate emergency care if needed – are justifiable (Kehl 2018b). Again, this largely depends on the normative stance one adopts. Conflicts arising from the design of robots are less frequently discussed. Weßel et al. (2021) argue that designing robots based on problematic gender stereotypes (e.g., portraying women as subservient and gentle) can enhance the well-being and robot acceptance of care recipients who grew up with these stereotypes, but potentially at the expense of societal progress toward gender diversity.

Further unsettled aspects noted in the literature do not concern conflicts but rather conceptual gaps that complicate decision-making. One such gap is the question of how to operationalize the concept of responsibility in the context of social robots: Who bears *responsibility* for the actions of social robots, particularly when the robot causes harm or negative consequences for users (Feil-Seifer and Matarić 2011)? This is not a trivial question, as certain hazards only arise due to a lack of clear ascriptions of responsibilities and liabilities—for example, the risk that care recipients face prohibitive costs if a care robot damages property because designers, vendors, or care facilities are not legally assigned responsibility (Schmidhuber and Stöger 2021).

There is also broader uncertainty regarding the concept of “good care”. How we understand (good) care will heavily influence how we evaluate the employment of social robots. Further unsettled questions are summarized in Table 5.

**Table 5:** Unsettled questions in the use of social robots in care for older people

Main category	Subcategory (first-level)
Unsettled questions	Do the benefits of social robots outweigh the costs?
	Who should receive/how to allocate scarce (robotic) resources?
	Whose interests regarding social robot usage should be prioritized?
	Design by stereotype to foster wellbeing/compliance while reproducing harmful effects/reducing acceptance of diverse ways of life?
	What privacy risks are acceptable for security gains?
	Is deception about relationships acceptable to realize benefits?
	Should control/access of social robots be restricted to reduce risk of harm?
	Who should/how to decide when people are cognitively impaired?
	Who bears responsibility for the social robot and its actions?
	(When) should social robots be allowed to act without consent to protect?
	Should autonomy be bestowed onto social robots (at all/to what extent)?
	What normative status should we assign social robots?
	How do we understand the concept of care?

## Discussion

Our results testify to a vast array of ethical aspects to consider when using social robots in care practices. This includes addressing the interests from different stakeholder groups as well as various value dimensions. As our findings indicate, most of these dimensions entail both potentially negative as well as positive ethical aspects.

From a perspective of decision-making – broadly defined as seeking normative action guidance and practical orientation – the discourse captured within this review may initially appear to lead to an impasse, offering little actionable guidance for concrete scenarios. However, it is important to understand that the actual realization of most of the identified ethical aspects is largely contingent on the specific circumstances of each situation (Lee et al. 2017). This implies that sweeping judgements about the use of social robots in care for older adults are often misplaced (Wahl et al. 2021) and fail to reflect the depth and complexity of ethical considerations outlined in this discourse. Secondly, it suggests that any practical application of this knowledge requires careful analysis of the specifics of the situation. This includes essential aspects such as the care setting, the type of the device used, its intended role, and the tasks assigned to it. Perhaps most importantly, it requires consideration of the characteristics, interests, and preferences of care receivers and care givers (Geier et al. 2020).

With this, our results also testify to some key challenges arising when making ethical decisions about the use of social robots from a practical perspective. The first key challenge is about being aware of and acknowledging the different needs and interests connected to the various perspectives involved. It is well-known, for example, that social robots in care for older adults are often ill-prepared to fulfill tasks care providers/recipients consider relevant or helpful (Frennert and Östlund 2014; Kehl 2018b, c) and decision makers do not know how and whose interests are to be taken into account. One reason is that development is often driven more by technical motivations and possibilities than actual care needs. It is, therefore, crucial that decision makers are aware of the plurality of perspectives and interests and enable greater engagement with affected user groups. This applies especially during early decisions such as in the design and testing process but is not limited to that. As a result of growing experience and becoming more acquainted with a technology, interests may evolve during later stages, necessitating periodic reassessments (Flandorfer 2012). In addition, any assessment of social robots used in a care setting is highly dependent on the specifics of the situation. A monitoring function of a robot can, for example, in some contexts be perceived as enhancing independence by allowing individuals to remain at home for longer, while in other contexts, it may be viewed as a critical infringement on privacy. Such functions may accordingly be welcomed or rejected, depending on the particular circumstances (Frennert and Östlund 2014) but also depending on user preferences (Buhr et al. 2025). Participatory approaches to the design and implementation of social robots have been highlighted as a useful strategy to simultaneously ensure that the interests of the affected user groups are respected and that robots are practical, for example, in terms of meeting the structural requirements (Lee et al. 2017).

Secondly, we note the importance for decision-makers to recognize that the discourse on social robots, as captured with this review, is not always substantiated by empirical evidence. As social robotics is still in an early stage, much of the discourse exists in the realm of speculative hope and fear. Whether all identified hazards and opportunities are genuinely relevant will need to be evaluated against actual and future developments. For instance, the perpetuations of problematic (gender-) stereotypes are more likely when robots take anthropomorphic forms, but it remains uncertain whether technological development will continue in this direction. In addition, many claims about ethically relevant outcomes such as impact on quality of life, are not only highly intervention-, device-, and setting-specific but currently lack sufficient empirical support (Haltaufderheide et al. 2023b).

A third aspect that decision-makers, or others working with our results, should consider, is that while the realization of many hazards and opportunities depends on how robotically assisted care practices are implemented, some opportunities will only be achieved by accepting the materialization of certain hazards. These aspects are categorized as unsettled questions, as they require to take a normative position on the hierarchies of values and the permissibility of value trade-offs. One example is the use of Paro and other similar zoomorphic robots to improve the well-being of dementia patients, which may only be achievable by accepting a degree of deception – an issue, often seen as morally problematic (Sparrow 2002; Coeckelbergh 2015; Remmers 2018). Another example concerns possible privacy breaches caused by constant monitoring through social robots, which may or may not be deemed acceptable in exchange for enhanced security, particularly of dementia patients. We do not claim to have identified all unsettled questions that require such value-balancing but consider it highly likely additional trade-offs can be reconstructed from the hazards and opportunities highlighted in specific situations.

Finally, we note that terminology and concepts in the research discourse are often used imprecisely, complicating debates about robots, care, and old age. For instance, it is often argued that social robots can relieve caregivers, allowing them more time for social interactions with care recipients. However, this seems contradictory, as social robots are explicitly designed to take on or support social interactions themselves. Interestingly, many examples given in the texts included in the analysis focus on tasks unrelated to social interaction, such as disinfecting door handles, transporting objects (Bleuler and Caroni 2021), or lifting (Parviainen et al. 2019), which could just as effectively be performed by simpler assistance robots or non-robotic systems (Zöllick et al. 2020). This raises the question of whether the development of social robots should be prioritized over non-social robotic systems that may achieve similar benefits without the associated ethical hazards. At the same time, it is important to consider that routine tasks, such as carrying objects or dressing (Parviainen and Pirhonen 2017), often provide moments of interpersonal contact, and it is worth reflecting on how care recipients might be affected if these interactions are carried out by robots. Furthermore, terms like “social robots” are not morally neutral, as they evoke specific images that may not accurately reflect the robots’ actual functions. For example, the robot Pepper might be better described as an entertainment robot, as it is primarily used for games (e.g., Bingo or Memory) or as a fitness instructor.

Another important concept shaping the ethical assessment of social robotics in care for older people – often implicitly – is the understanding of “care” or “good care” (Coeckelbergh 2015). If “good care” is equated with human care (von Maur 2023) or necessarily involves interpersonal relationships (Deutscher Ethikrat 2020), this results in different evaluation standards for the use of social robotics than if “good care” is equated with the fulfillment of specific tasks (Sparrow 2016; Suwa et al. 2020). Misselhorn (2018) provides a helpful distinction in this context, between goal-oriented or practice-oriented care. She describes goal-oriented care as focusing on outcomes (e.g., bringing a needed object to the care recipient), while practice-oriented care emphasizes the social and empathic interactions inherent in caregiving activities. Robots are currently incapable of practice-oriented care, at least according to the majority opinion (Bertolini and Arian 2020; Noori et al. 2019; Steinrötter 2020; von Maur 2023). Consequently, those who adopt a practice-oriented view of care are likely to reject social robots as adequate tools in care for older people. In the literature, the concept of care is repeatedly used without much reflection. For robust evaluation and meaningful discussion, it would be important to make the underlying understanding of care in this context explicit (Kehl 2018a).

The same applies with regard to underlying perceptions of images of old age which warrant caution (Neven 2010). How old age is perceived not only influences how the prospect of care being provided by robots is evaluated but also determines the type of robots that are designed and implemented. A recurring issue is that the technical perspective on social robotics in care for older adults is often characterized by deficit-oriented images of old age, reducing older people in need of care to technical problems requiring solutions (Frennert and Östlund 2014; Kamphof 2015). A further problem is that older people are frequently viewed as a uniform group, neglecting the fundamentally different preferences, values, and priorities that exist – just as in any other human group. Such perspectives not only objectify older adults but also neglect the creativity and potential for self-determined agency in old age (Remmers 2019). Moreover, they fail to account for individual needs, which can vary widely depending on personal experiences, cultural backgrounds, and specific circumstances. Acknowledging and addressing these diverse needs is essential to developing social robots that genuinely enhance quality of life of older adults, while respecting their own values. In our opinion, it is desirable for the development of social robots in care for older adults to focus not only on compensating for lost abilities (“problem-solving”) but also on enabling older people to participate productively in society (Remmers 2019).

## Strengths and limitations

With this review we provide a comprehensive overview on the debates and arguments surrounding the use of social robotic care technologies from a decision-centric perspective. Several prior reviews have addressed related dimensions of our topic, for example, by providing overviews on the empirical evidence relevant to ethical considerations (Haltaufderheide et al. 2023a, b), by surveying ethical positions in argument-based literature (Vandemeulebroucke et al. 2018), or by reviewing ethical issues (Boada et al. 2021). In relation to those works, however, our review differs in that we take an explicitly descriptive and analytic perspective aimed at synthesizing main strands of ethical arguments and conceptual positions (rather than empirical observations or normative foundations) relevant to practical considerations of implementing social robots. Our findings come with certain limitations we want to highlight. First, although our search strategy covered key interdisciplinary databases and specialized ethics repositories, it remains possible that relevant publications indexed elsewhere were missed. However, given the overlapping coverage of the selected databases, including general and specialized sources, we expect the risk of omission to be low. 

Second, the operationalization of key terms, particularly the ethical aspects that largely determined the scope of our review, warrants caution. We based our framework for operationalization on principlism, adapted from Beauchamp and Childress (2019) for the context of robots (Floridi et al. 2018). Although this approach of operationalizing is debated, it is widely applied in normative systematic reviews (Mertz et al. 2017) and proved effective for our purposes. We deliberately avoided relying solely on authors’ self-identification of ethical aspects, as non-ethicists often do not use normative terminology, potentially leading to relevant points being overlooked. However, our approach may have had exclusionary effects as well – such as missing discussions clarifying normative concepts like responsibility or good care.

Third, our findings might be less reliable due to the interpretative nature of qualitative systematic reviews and the fact that most included literature was analyzed by only one reviewer. This decision was made to manage the unexpectedly high number of publications. To mitigate the risks of solo interpretation, the first 25 publications, which represented a variety of disciplines and formats, were analyzed by two to three reviewers. This subset helped establish a robust coding frame, which was then applied to the remaining publications. Additionally, regular meetings were held between the two reviewers and the project lead to address any challenges, uncertainties, or emerging codes.

Finally, due to the lack of an appropriate methodology, we have decided to not conduct a quality appraisal as part of our review. Methodological standards for appraising the quality of normative analyses remain contested and under development. Readers are therefore encouraged to engage critically with the arguments and interpretations presented, acknowledging the interpretive and context-dependent nature of ethical reasoning.

## Conclusion

With this work, we provide a comprehensive overview of the ethical hazards and opportunities that should be considered in practical evaluations of the use of social robots. Our findings demonstrate that the research discourse provides a vast array of potential ethical aspects. Its structure implies, however, that decision makers need to be aware of its complexity and depth, as well as its empirical and conceptual weaknesses and the various affected interests and needs involved to be able to make ethically informed decisions.

## Supplementary Information

Below is the link to the electronic supplementary material.

Online Resource 1 (PDF 404 kb)

Online Resource 2 (PDF 171 kb)

Online Resource 3 (PDF 159 kb)

Online Resource 4 (PDF 88 kb)

## Footnotes

1 E.g., Giraff, a telepresence robot able to connect users with their loved ones or care providers virtually (Ienca et al. 2016).

2 E.g., Pepper, a humanoid robot able to talk and used, for example, as a fitness instructor or storyteller in care facilities (Ghafurian et al. 2022; Pandey and Gelin 2018).

3 The term “elderly care” has become criticized for being ageist and we have therefore avoided it throughout the manuscript except the method section: As the term was and still is commonly used in this context, it was helpful to employ it in our search strategy which we wanted to make transparent.

4 For a more detailed description of the inclusion/exclusion criteria and the underlying rationale the protocol can be consulted.
