# Supplementary material for: Ethical aspects of the use of social robots in caring for older people – a systematic qualitative review
Source: Med Health Care Philos. 2026 Feb 5;29(1):209–24. doi: 10.1007/s11019-025-10313-3 (PMC12960314; doi:10.1007/s11019-025-10313-3)
Supplement: Supplementary file 1 — Online Resource 1 (PDF 404 kb) [file 11019_2025_10313_MOESM1_ESM.pdf]

# Online Resource 1 to: Ethical Aspects of the Use of Social Robots in Elderly Care

## A Systematic Qualitative Review

Marianne Leineweber[1], Clara Victoria Keusgen[1], Marc Bubeck[1], Robert Ranisch[1*], Joschka Haltaufderheide[1†], Corinna Klingler[1†]

[1]Juniorprofessorship for Medical Ethics with a focus on Digitization, Faculty for Health Sciences Brandenburg, University of Potsdam, Am Mühlenberg 9, Potsdam, 14476, Brandenburg, Germany.

*Corresponding author(s). E-mail(s): ranisch@uni-potsdam.de;
†Joschka Haltaufderheide and Corinna Klingler contributed equally as last authors.

# Categories with example quotes

- Ethical opportunities for care recipients
  - Improving/ensuring well-being/health → "Research shows that when older adults form relationships with robots, they generally report better health and well-being (Broekens et al. 2009)."(Jecker 2021, p. S36)
    - Supporting therapeutic/re-habilitative procedures → "Yet, social robots cannot only entertain and engender social interaction, they may also be used to initiate therapeutic processes that enhance self-understanding." (Misselhorn et al. 2013, p. 128)
      - (Re-)activation of (lost) capacities → "Social assistive-robot-based systems with the abilities to perform activities, play cognitive games, and socialize, can be used to stimulate the physical, cognitive, and social conditions of older adults and restrain deterioration of their cognitive state [20]."(Mishra et al. 2021, p. 492)
      - Remote doctor's appointment as facilitation → "Nowadays a large number of companies are developing robots to monitor the health and safety of old citizens. Some robots like RP-7 are teleoperated so the elderly can interact with their family doctors instantly." (Noori et al. 2019 Robot-Care for the Older People: Ethically Justified or Not?, S. 44)
    - Reducing risk of mistreat-ment by care providers → "The primary aim of robots is to make senior citizens independent, which sometimes they dont [sic] feel comfortable with caregivers or some care-givers dont [sic] treat/care them according to the human rights act."(Noori et al. 2019 Robot-Care for the Older People: Ethically Justified or Not?, p. 44)
      - Reducing risk of abuse/neglect (e.g. stemming from caregiver fatigue) → "Psychologically, assuming that robotics technology has been programmed to act ethically, employing robotics and autonomous systems to deal with some of the caregiving tasks for the older people could potentially reduce the likelihood of psychological and emotional abuse and neglect which often stem from caregiver fatigue (O'Brolchain, 2017)." (Tan et al. 2021 Tensions and antagonistic interactions of risks and ethics of using robotics and autonomous systems in long-term care, p. 2)
      - Controlling care personnel and reducing problematic practices → "Employing the robot to "police" care may discourage poor care practices, with benefits to older people." (Draper and Sorell 2017 Ethical values and social care robots for older people: an international qualitative study, p. 63)
    - Addressing physical well-being and safety of care recipients → "Robots are an opportunity for the health and social care sector. They increase individual autonomy, relieve staff of repetitive tasks and contribute to the safety of residents, for example, by detecting falls." (Translation by the authors) (Früh and Gasser 2018 Erfahrungen aus dem Einsatz von Pflegerobotern für Menschen im Alter, p. 45; in: Bendel 2018 Pflegeroboter)
      - *Cont. on next page*
    - *Cont. on next page*
  - *Cont. on next page*

Ethical opportunities for care recipients (continued)

- Improving/ensuring well-being/health (continued)
  - Addressing physical well-being and safety of care recipients (continued)
    - (Reducing risk to catch infectious diseases (e. g. from care providers) → "First, sociable robots can be sanitized and offer a safe means of interacting with older people during a pandemic (Armitage and Nellums 2020)." (Jecker 2021 You've got a friend in me: sociable robots for older adults in an age of global pandemics, p. S36)
    - Motivate or execute behavior to reduce (health) risks → "A robot could register potential dangers; for example, turn off a forgotten hotplate; prevent an elderly person from climbing onto a wobbly chair to reach an object; but also simply take high-calorie food away from a person who should be watching their weight for health reasons or prevent them from pouring themselves a third glass of wine." (Translation by the authors) (Bioethikkommission beim Bundeskanzleramt Österreich 2018 Roboter in der Betreuung alter Menschen (Stellungnahme), p. 368)
  - Monitoring health and alarming others in crisis situations → "In both cases, a robot is used in the household - in the case of SRS the Care-O-Bot, in the case of Robo M.D. the Nao (Aldebaran), which detects the senior's state of health and passes it on to a medical center or reports critical situations to relatives, neighbors, family doctors, emergency doctors and/or relatives." (Translation by the authors) (Kapitel Meyer 2011 Akzeptanz ausgewählter Anwendungsszenarien, p. 89; in: Meyer 2011 Mein Freund der Roboter - Servicerobotik für ältere Menschen - eine Antwort auf den demographischen Wandel?)
    - As instructor in fitness sessions → "Based on the recommendation of her physiotherapist, the robot demonstrates some soft and slow stretching motions that Mrs. Smith can replicate." (Gelin 2017 The Domestic Robot: Ethical and Technical Concerns, p. 208; in: Ferreira et al. 2017 A World with Robots - Intelligent Systems, Control and Automation: Science and Engineering)
    - Motivating movement/exercise/free-time activities → "Her Care-O-bot® knows that she should be encouraged to move about, and suggests several times a day that she walks with it to look out of the window at either the garden or the street below." (Draper and Sorell 2017 Ethical values and social care robots for older people: an international qualitative study, p. 53)
    - Sex robots allow staying sexually active → "[. . . ] finally, after responding to objections, I conclude that sex robots are a reasonable way to support later-life sexuality for persons with disabilities." (Jecker 2021 Nothing to be ashamed of - sex robots for older adults with disabilities, p. 26)
    - *Cont. on next page*
  - *Cont. on next page*
- *Cont. on next page*

Ethical opportunities for care recipients (continued)

- Improving/ensuring well-being/health (continued)
  - Addressing physical well-being and safety of care recipients (continued)
    - Positive physiological reactions (e. g. of organs) → "We consider that the interaction with Paro and with other people (through Paro) physiologically improved the reactions of the residents' vital organs." (Wada und Shibata 2007 Living With Seal Robots – Its Sociopsychological and Physiological Influences on the Elderly at a Care House, p. 978)
  - Addressing emotional well-being of care recipients → "Khosla and Chu proposed the design and implementation of a human-like assistive robot Matilda to improve the emotional wellbeing of the elderly in residential care facilities." (Wang et al. 2019 Affective Interaction Technology of Companion Robots for the Elderly: A Review, p. 79)
    - Reducing boredom → "Positive expectations of the caregivers were that the robotic cushion ["Qoobo"] would be more practical than the already used SAR, that it could improve mood, would be a pastime and boredom would be reduced." (Lehmann et al. 2021 Using a Socially Assistive Robot in a Nursing Home: Caregivers' Expectations and Concerns, p. 148)
    - Reducing worry/fear → "The main reported benefits of Paro at home were in reducing anxiety and enhancing mood. Caregivers reported that touching Paro's soft texture was soothing for some care recipients and that it could serve as a distraction from distressing events." (Liang et al. 2017 A Pilot Randomized Trial of a Companion Robot for People With Dementia Living in the Community, p. 875)
    - Reducing feelings of loneliness/enhancing feelings of attachment → "Of particular interest here is the case in which a 76-year-old male resident in a nursing home who is recovering from depression is given a My Real Baby to interact with for 4 months. As a natural animal lover who considers all the neighborhood animals his friends (and believes that he communicates with them), he responds warmly to the introduction of My Real Baby. He explains to the researchers that he not only enjoys the robots presence as it alleviates feelings of loneliness, but that he also talks to the robot as if it were his former wife; its presence and responsiveness gives him some sort of permission to vent his private feelings of attachment, regret, and confusion." (Misselhorn et al. 2013 Ethical Considerations Regarding the Use of Social Robots in the Fourth Age, p. 128)
    - Reducing stress → "An interesting possibility that could be explored in future research is that some of the benefits that result from playing with, or "nurturing" robot pets are a consequence of increased levels of oxytocin, which as discussed earlier, appears to reduce stress." (Sharkey and Sharkey 2012 Granny and the robots: ethical issues in robot care for the elderly, p. 34)
    - *Cont. on next page*
  - *Cont. on next page*
- *Cont. on next page*

- Ethical opportunities for care recipients (continued)
  - Improving/ensuring well-being/health (continued)
    - Addressing emotional well-being of care recipients (continued)
      - Enhancing the mood and other positive neuropsychological effects → "In nursing homes in Japan, "Qoobo" was found to have positive effects on psychological well-being, such as patient satisfaction, happiness, and mood changes [34]." (Lehmann et al. 2021 Using a Socially Assistive Robot in a Nursing Home: Caregivers' Expectations and Concerns, p. 150)
      - Reassurance/confirmation of worth by social robot → "The residents' reassurance of worth could be strengthened by both direct and indirect social robots. Direct social robots could address people using their favorite 'titles' (whether from their previous profession or private life) and by their name (reassurance of worth) and ask questions about daily life in the old times or recent history, thus eliciting positive reminiscence." (Pirhonen et al. 2020 Can robots tackle late-life loneliness? Scanning of future opportunities and challenges in assisted living facilities, p. 8)
      - Sex robots can reduce shame/negative feelings (e. g. around sexuality) → "Sex robots could be used to practice sexual dysfunction without fear or shame [. . . ]." (Translation by the authors) (Döring 2018 Sollten Pflegeroboter auch sexuelle Assistenzfunktionen bieten?, p. 260; in: Bendel 2018 Pflegeroboter)
      - Social robots experienced as less pressurizing than care providers → "The situation was also unpleasant for the person in need of care, as they felt forced to eat. The calm and consistently monotonous nature of Care-O-bot's communication not only relieved the care staff of the repetitive and mentally stressful request to eat, but also made the patient feel less coerced." (Translation by the authors) (Scorna 2015 Servicerobotik in der Altenpflege – Eine empirische Untersuchung des Einsatzes der Serviceroboter in der stationären Altenpflege am Beispiel von PARO und Care-O-bot, p. 91; in: Weber et al. 2015 Technisierung des Alltags – Beitrag für ein gutes Leben?)
  - Restoring privacy → "While they repeatedly acknowledge the importance of respecting older persons' privacy, the AEG [Alzheimer Europe's Guidelines and Position on the Ethical Use of Assistive Technologies for/by People with Dementia] also mention a different view of the relationship between SARs and privacy, pointing out how relying on robot assistance may actually enhance privacy and not interfere with it." (Battistuzzi et al. 2018 Embedding Ethics in the Design of Culturally Competent Socially Assistive Robots, p. 1999)
    - Intimate situations have no longer to be shared with a person → "In the area of intimate care or in other very personal areas, support from machines is less invasive of privacy for some people than support from humans." (Translation by the authors) (Bauberger 2020 Medizin und Pflege, p. 71; in: Bauberger 2020 Welche KI? Künstliche Intelligenz demokratisch gestalten)
    - *Cont. on next page*
  - *Cont. on next page*

Ethical opportunities for care recipients (continued)

- Restoring privacy (continued)
  - Care providers might be nosey/-judgy, social robots are not → "There is a shared understanding of the potential normative implications of a carer's seeing an unexpected person sharing the householder's bed, overhearing a phone call to the betting office or alcohol retailer, or reading aloud a letter from a solicitor about changes to a will. If confronted by a wife or child asking questions about these events, a human servant/carer/companion has to make a normative judgement about the relative importance of infidelity, gambling, alcohol use and disinheriting a family member compared with some prior agreement to maintain confidentiality. For the robot there is no such tension. This could be regarded by some older people as a potential advantage of having robot as a carer. The robot is not nosey—it has no personal interest in finding certain things out, items of information are merely data." (Draper and Sorell 2017 Ethical values and social care robots for older people: an international qualitative study, p. 64)
- Promoting positive perception/preventing stigmatization/discrimination of care recipient → "Studies indicate that in some situations it may be less stigmatising for the older user to receive robotic care or help instead of human [34]." (Frennert and Östlund 2014 Review: Seven Matters of Concern of Social Robots and Older People, pp. 301, 302)
  - Sex robots produce counter (non-ageist) narratives about sexuality → "One way this can occur is by generating counternarratives that include sex robots. Robots designed to integrate with the life narratives of older users may bear little resemblance to contemporary sex robots that dominate film and television shows, such as the young gynoids, Ave and Kyoko, of the 2014 film Ex Machina or those featured in the 1976 television series The Bionic Women and the Austin Powers film series. These images tilt towards users who are young, able-bodied, heterosexual and male. By contrast, sex robots designed for older people might resemble a deceased spouse in appearance or manner. For an older user, a desirable sex robot might evoke film stars of a bygone era, like Sidney Portier or Bette Davis." (Jecker 2020 Nothing to be ashamed of: sex robots for older adults with disabilities, p. 29)
- Providing support in daily living (esp. where capacities reduced) → "The older person becomes something that needs to be governed and the robot assists the person in her everyday life [31]. Examples of robots that fit into this paradigm are Fraunhofer's Care-O-Bot [...]" (Frennert and Östlund 2014 Review: Seven Matters of Concern of Social Robots and Older People, p. 302)
  - By acting as memory aid → "For example, Fraunhofer IPA's Care-o-bot (now at its 4th generation, Care-O-bot 4), has been successfully tested to assist the specific memory deficits of older adults with dementia [...]." (Ienca et al. 2016 Social and Assistive Robotics in Dementia Care: Ethical Recommendations for Research and Practice, p. 566)
    - Reminding of important appointments/activities (e. g. taking medication) → "Cognitive assistance care robots may meet the need for senior citizens to live independently at home for a longer time. A robot can assist someone to remember appointments, to take medication or to eat on time [...]." (Van Est et al. 2016 Robotisation as Rationalisation - In Search for a Human Robot Future, p. 45)
    - *Cont. on next page*
  - *Cont. on next page*
- *Cont. on next page*

Ethical opportunities for care recipients (continued)

- Providing support in daily living (esp. where capacities reduced) (continued)
  - By acting as memory aid (continued)
    - Reminding how things get done → "Cognitive assistance care robots may meet the need for senior citizens to live independently at home for a longer time. A robot can assist someone to remember appointments, to take medication or to eat on time [...]." (Van Est et al. 2016 Robotisation as Rationalisation - In Search for a Human Robot Future, p. 45)
    - Reminding of further relevant aspects of daily living → "Small social robots that help you learn a foreign language, remind you of medication, save birthdays or shopping lists are unusual but relatively harmless in the eyes of the test persons." (Translation by the authors) (Meyer 2011 Akzeptanzbedingungen für Roboter-Assistenten, p. 106; in: Meyer 2011 Mein Freund der Roboter - Servicerobotik für ältere Menschen - eine Antwort auf den demographischen Wandel?)
  - By carrying out certain tasks for/with the care recipient → "It would be ideal if the user and the robot could perform tasks together. When performing a task together, the robot would only need to perform that part of the task the user cannot perform. For example, when the user is still able to make a cup of coffee but unable to carry the cup of coffee to the living room, the robot should do that latter part of the task for the user." (Bedaf et al. 2019 What are the preferred characteristics of a service robot for the elderly? A multi-country focus group study with older adults and caregivers, p. 8)
    - Supporting eating/feeding → "Other 'class 2 medical robots' are assistive robots (typically mobile robots and mobile manipulators) that help impaired people to everyday functions, such as eating, drinking, washing, etc. or for hospital operations. Examples of such robots are: the 'Care-0-Bot 3' robot [...]." (Tzafestas 2016 Socialised Roboethics, p. 110; in: Tzafestas 2016 Roboethics - A Navigating Overview)
    - Supporting having sex → "I am simply arguing that sexbots are a plausible solution that could help residents who still have sexual drives but are currently unable to find a suitable outlet for them." (Lancaster 2023 Granny and the Sexbots: An ethical appraisal of the use of sexbots in residential care institutions for elderly people, p. 200; in: Loh and Loh 2023 Social Robotics and the Good Life - The Normative Side of Forming Emotional Bonds With Robots)
    - Supporting with getting up and moving around → "However, general support in moving around the residence was viewed more positively. A robot which was remotely operated and monitored by care professionals could be used in the homes of older people as an assistant for moving, walking and getting up." (Parviainen et al. 2019 Social Robots and Human Touch in Care: The Perceived Usefulness of Robot Assistance among Healthcare Professionals, p. 10)
    - *Cont. on next page*
  - *Cont. on next page*
- *Cont. on next page*

- Ethical opportunities for care recipients (continued)
  - Providing support in daily living (esp. where capacities reduced) (continued)
    - By carrying out certain tasks for/with the care recipient (continued)
      - Supporting with doing household chores → "It has been demonstrated that most older people could accept robots as functional helpers in the home doing household chores [. . . ]." (Frennert and Östlund 2014 Review: Seven Matters of Concern of Social Robots and Older People, p. 304)
      - Accompanying to/support with appointments and shopping → "The idea of using service robots as shopping guides is aimed at the problems associated with shopping. The basic idea is that the service robot waits in the entrance area of a shopping center, asks the customer what they want and then guides them to the goods they are looking for. If the barcode of the product is held under the built-in scanner, it can also answer questions about the prices of the desired items or the ingredients of the food. If desired, the shopping assistant can guide the customer to the checkout." (Translation by the authors) (Kapitel Meyer 2011 Akzeptanz ausgewählter Anwendungsszenarien, p. 72; in: Meyer 2011 Mein Freund der Roboter - Servicerobotik für ältere Menschen - eine Antwort auf den demographischen Wandel?)
      - Getting relevant things for care recipients → "The third generation of the robot, Care-O-bot III, was already able to move independently and collision-free between people and provide them with household items or drinks of their choice, among other things." (Translation by the authors) (Kreis 2018 Umsorgen, überwachen, unterhalten - sind Pflegeroboter ethisch vertretbar? p. 218; in: Bendel 2018 Pflegeroboter)
      - Providing relevant public information (e. g. weather) → "Lio and P-Care can answer questions about the weather or the latest news and thus serve as conversation partners for the residents." (Translation by the authors) (Früh and Gasser 2018 Erfahrungen aus dem Einsatz von Pflegerobotern für Menschen im Alter, p. 39; in: Bendel 2018 Pflegeroboter)
      - Carrying out further tasks (e. g. guarding the home against intruders) → "Aido, a robot from the company InGen Dynamics (InGen Dynamics n.d.), can move around the house and is primarily intended for interaction with people (Aido User Manual n.d.). On the one hand, it has various entertainment functions, but can also be used to monitoring the house or as a personal assistant." (Translation by the authors) (Bleuler and Caroni 2021 Roboter in der Pflege - Welche Aufgaben können Roboter heute schon übernehmen?, pp. 445, 446; in: Bendel 2021 Soziale Roboter)
  - Sustaining independent and established lifestyle → "On the other hand, robots that remind people to take their medication or monitor them, for example, are designed precisely for cognitively impaired people so that they can live more independently despite their limitations." (Translation by the authors) (Bleuler and Caroni 2021 Roboter in der Pflege - Welche Aufgaben können Roboter heute schon übernehmen?, p. 444; in: Bendel 2021 Soziale Roboter)
    - *Cont. on next page*
  - *Cont. on next page*

Ethical opportunities for care recipients (continued)

- Sustaining independent and established lifestyle ) (continued)
  - Allow sustaining established routines/relations of daily living → "They are potentially important not only in relieving loneliness but in helping their users maintain a normal routine in the face of frailty [. . . ]." (Draper and Sorell 2017 Ethical values and social care robots for older people: an international qualitative study, p. 50)
  - Remaining in own home for longer → "To avoid moving to a nursing home or other care facility, Margaret has purchased a home-care robot that can: remind her to take her medications; fetch items for her if she is too tired or is already in bed; help with simple cleaning tasks; and, can facilitate her staying in contact with her family, friends and healthcare provider via video chat."(van Wynsberghe 2015 Designing Care Robots with Care, p. 9; in: van Wynsberghe 2015 Healthcare Robots - Ethics, Design and Implementation)
  - Reducing dependencies on other people → "The option to rely on assistive technology systems instead of being dependent on human caregivers can thus prima facie be understood as a valuable option of gaining a degree of independence."(Wiertz 2020 Trusting robots? – On the concept of trust and on forms of human vulnerability, p. 53; in: Haltaufderheide et al. 2020 Aging between Participation and Simulation - Ethical Dimensions of Social Assistive Technologies)
  - Social robots are flexible and can help at any time (e. g. at night) → "The robots can also be called around the clock to provide assistance to people in the middle of the night or early in the morning." (Translation by the authors)(Früh and Gasser 2018 Erfahrungen aus dem Einsatz von Pflegerobotern für Menschen im Alter, p. 45; in: Bendel 2018 Pflegeroboter)
- Furthering social connection → "Social networks change over the lifespan, with the average size of private networks already decreasing from a maximum in the fourth decade of life [23]. In order to possibly compensate for the impending loss of resources, there could be an increasing number of technical possibilities offered by social robotics." (Translation by the authors) (Zöllick et al. 2022 Potenziale und Herausforderungen von sozialen Robotern für Beziehungen älterer Menschen: eine Bestandsaufnahme mittels 'rapid review'", p. 299)
  - Social robots offer opportunities for social interaction with them → "The performance of social or socio-emotional robots initially consists less in performing certain activities than in entering into social interaction with people." (Translation by the authors) (Manzeschke 2019 Roboter in der Pflege - Von Menschen, Maschinen und anderen hilfreichen Wesen, p. 4)
    - *Cont. on next page*
  - *Cont. on next page*
- *Cont. on next page*

- Ethical opportunities for care recipients (continued)
  - Furthering social connection (continued)
    - Social robots offer opportunities for social interaction with them (continued)
      - Social robots as companions → "During a pandemic emergency in particular, the alternative to robot companionship for many older people is social isolation and loneliness. Without support, older adults are left to languish. Under these conditions, sociable robots do not rob older adults of human companionship but afford companionship where it is lacking."(Jecker 2021 You've got a friend in me: sociable robots for older adults in an age of global pandemics, p. S39)
      - Social robots as entertainers → "Various functions are used to entertain and activate residents. The assistance robot cheers them up with jokes and short stories." (Translation by the authors)(Wirth et al. 2020 Erfahrungen aus dem Einsatz von Assistenzrobotern für Menschen im Alter, p. 261; in: Buxbaum 2020 Mensch-Roboter-Kollaboration)
      - Sex robots as (safe) sexual contacts → "[. . . ] the advantage of sex robots is that they can meet sexual needs without violating the rights of any other person."(Jecker 2021 Sex robots for older adults with disabilities: reply to critics, p. 1)
    - Social robots facilitate interaction with other human beings → "Nevertheless, the developments in robotics - if viewed within these limits - give rise to justified hope for an improvement in the care situation. Such systems can help to maintain communication and the ability of people to meet in situations where other options are not available [...]." (Translation of the authors)(Gräb-Schmidt and Stritzelberger 2018 Ethische Herausforderungen durch autonome Systeme und Robotik im Bereich Pflege, p. 368)
      - By translating if care recipient and other actors don't speak same language → "Due to progressive dementia, Adam, who is in need of care, speaks almost exclusively his native language and does not even realize that he is doing so. This means that the care staff can no longer understand the person in need of care. The robot recognizes such situations through speech recognition, which is supported by artificial intelligence. It then translates into the other language in such situations. In this way, the person in need of care knows that the meal is ready and can let the carer know when he is thirsty. It also gives the senior the opportunity to continue speaking his native language." (Translation by the authors)(Carros et al. 2022 Roboter als intelligente Assistenten in Betreuung und Pflege – Grenzen und Perspektiven im Praxiseinsatz, p. 808; in: Pfannstiel 2022 Künstliche Intelligenz im Gesundheitswesen)
        - *Cont. on next page*
      - *Cont. on next page*
  - *Cont. on next page*

Ethical opportunities for care recipients (continued)

- Furthering social connection (continued)
  - Social robots facilitate interaction with other human beings (continued)
    - Care providers have more time for social interaction → "Due to the assumption of various smaller tasks, the interviewees assume that robots will relieve the nursing staff in their everyday work and thus give them more freedom. This will benefit nursing home residents, who will be able to have other activities and conversations with nursing and care staff or benefit from less stressed staff (Lipp & Massen, 2019)." (Translation by the authors) (Carros et al. 2022 Roboter als intelligente Assistenten in Betreuung und Pflege – Grenzen und Perspektiven im Praxiseinsatz, pp. 811, 812; in: Pfannstiel 2022 Künstliche Intelligenz im Gesundheitswesen)
    - Providing something to talk about (communication facilitator) → "On the one hand, social robots can promote communication with them by initiating conversations. On the other hand, when used in a therapeutic group setting, they promote interpersonal communication about them, which in turn can strengthen social integration [20]."(Zöllick et al. 2022 Potenziale und Herausforderungen von sozialen Robotern für Beziehungen älterer Menschen: eine Bestandsaufnahme mittels "rapid review", pp. 300, 302)
    - Providing information on chances for interaction (e. g. events) → "Frank's daughter has suggested that the Care-O-bot should be used to encourage Frank join a virtual fishing forum on the internet. She is worried that he only has one friend who is older and poorly and may die leaving Frank with no one else to talk to about fishing." (Draper and Sorell 2017 Ethical values and social care robots for older people: an international qualitative study, p. 53)
    - Attracting visitors (particularly grandchildren) "A robot that facilitates conversation may function as an attractor for visitors." (Sharkey and Sharkey 2012 Granny and the robots: ethical issues in robot care for the elderly, p. 35)
    - Promoting positive/friendly communication → "For instance, when Wada and Shibata (2006) videoed interactions between a Paro robot seal and a group of elderly care home residents, they found evidence that the social interactions between the residents themselves increased, at the same time that physiological indicators showed reduced stress levels. It seems that Paro even encouraged positive communication, and resulted in a reduction of the "backbiting" that had previously characterised some of their interactions." (Sharkey and Sharkey 2012 Granny and the robots: ethical issues in robot care for the elderly, p. 35)
    - Allowing easy exchange via virtual interfaces → "Telepresence is a potential technology to alleviate loneliness by allowing friends and family members to be virtually present for the elderly person (e.g. [4]). [. . . ] For elderly persons, robotic telepresence provides benefits compared to non-mobile video connection [7]: because of the remote control of the robot, the elderly user can interact with it in a natural manner, with little additional learning."(Niemelä et al. 2021 Towards Ethical Guidelines of Using Telepresence Robots in Residential Care, p. 431)

*Cont. on next page*

Ethical opportunities for care recipients (continued)

- Supports tailoring care approaches to needs/taste → "Second, sociable robots that leverage recent advances in artificial intelligence (AI) are up to the challenge. They display increasingly sophisticated emotional intelligence; interact in ways that seem lifelike, such as recognizing voices, faces and emotions; interpret speech and gestures; respond appropriately to complex verbal and nonverbal cues; make eye contact; speak conversationally; and adapt to people's needs by learning from feedback, rewards, and criticisms."(Jecker 2021 You've got a friend in me: sociable robots for older adults in an age of global pandemics, p. S36)
  - Social robots will learn to read care recipient and react adequately → "Future robot fitness coaches will be able to correctly interpret the mood of their 'pupil' in order to find the right approach to motivate them. To do this, the fitness robot must be able to recognize people, recognize naturally spoken language and process gestures. This development could also be harnessed for older people and used in rehabilitation or prevention." (Translation by the authors)(Meyer 2011 Akzeptanz ausgewählter Anwendungsszenarien, p. 87; in: Meyer 2011 Mein Freund der Roboter - Servicerobotik für ältere Menschen - eine Antwort auf den demographischen Wandel?)
  - Simplified by broadening scope of action (more approaches to choose from) → "It is important however to observe the choice of the care recipient. Some people might prefer a human caregiver, while others may prefer the support of robots, depending on which one gives them a greater sense of self-worth." (Van Est et al. 2016 Robotisation as Rationalisation - In Search for a Human Robot Future, p. 45)
- Support/care provided (more) efficiently → "In view of the expected increase in the number of very old people living alone and those remaining alone in (rural) regions, telematics-supported systems are seen as promising technologies for improving care provision. The article shows the potential benefits that the implementation of telepresence robotics can have for both relatives and people in need of help in the case of dementia." (Translation by the authors)(Bleses and Dammert 2020 Neue Technologien aus Sicht der Pflegewissenschaft, p. 56; in: Hanika 2020 Künstliche Intelligenz, Robotik und autonome Systeme in der Gesundheitsversorgung)
  - Social robots can save costs (e. g. compared to smart homes) → "Gecko Systems claim that the CareBot provides a more cost-effective version of a "smart home", because the robot can follow the occupant from room to room, and reduce the need for sensors and wires throughout the house."(Sharkey and Sharkey 2012 Granny and the robots: ethical issues in robot care for the elderly, p. 31)
  - Pet robots easier to maintain than real animals → "In contrast to real animals, Paro has several advantages: for example, the seal cannot bite patients who grip it hard, does not trigger allergic reactions due to its antiseptic fur, does not cause dirt, for example through excrement, and does not need food. Paro is intended to offer patients who have problems caring for themselves an easy-care alternative to real animals (Calo et al. 2011: 21)." (Translation by the authors)(Lange and Bauer 2021 - Eine Robbe für Oma - Die zukünftige Dauerausstellung Robotik im Deutschen Museum, p. 231; in: Inthorn und Seising 2021 Digitale Patientenversorgung - Zur Computerisierung von Diagnostik, Therapie und Pflege)

Ethical hazards for care recipients (Main category)

- Reducing/not adequately securing well-being/health → "Sullins suggests that robotic companions can only satisfice, but not truly satisfy, our physical and emotional needs."(Frennert and Östlund 2014 Review: Seven Matters of Concern of Social Robots and Older People, p. 304)
  - Facilitating harmful behavior of care recipient → "Louis has discovered that he can use the interface on the Care-O-bot to visit online gambling sites and enjoys playing poker in the evening."(Draper and Sorell 2017 Ethical values and social care robots for older people: an international qualitative study, p. 53)
    - Social robot assists in dangerous actions/addictive behavior → "Frank, an elderly gentleman suffering from mild dementia, is given a robot by his son to help him with his everyday life. He only reluctantly accepts this housemate until he discovers that this robot can help him to resume his dormant activity as a jewel thief in an enormously effective way." (Translation by the authors) (Manzeschke 2019 Roboter in der Pflege - Von Menschen, Maschinen und anderen hilfreichen Wesen, p. 7)
    - Becoming lazy/moving less as social robot does it all → "Marie is reluctant to get up from her chair because she is afraid of falling and walking is uncomfortable. She also uses the Care-O-bot® to get drinks for her from the kitchen, even though the nurse has suggested that she should go to the kitchen with the Care-O-bot® but let it carry the drinks back to her chair for her."(Draper and Sorell 2017 Ethical values and social care robots for older people: an international qualitative study, p. 53)
  - Reduction in the quality of care provided → "The introduction of robotic care determined by limited stakeholders leaves customised care in danger and instead could potentially turn the quality of elderly care into 'elderly management'." (Poulsen and Burmeister 2019 Overcoming carer shortages with care robots: Dynamic value trade-offs in run-time, p. 1)
    - Introduction of social robots leads to disinvestment in care providers → "However, should such applications be intended as a way to radically reduce or worse eliminate professional human care, with the primary aim of abiding budgetary constraints grounded on the observed increase in age-related welfare costs, that would also contrast the anthropocentric perspective on the use of technology, and violate the dignity of the user, thereby being inadmissible in light of our extant legal system."(Bertolini and Arian 2020 Do robots care? - Towards an anthropocentric framework in the caring of frail individuals through assistive technology, p. 49; in: Haltaufderheide et al. 2020 Aging between Participation and Simulation - Ethical Dimensions of Social Assistive Technologies)
    - (Informal) caregivers withdraw care/are less engaged because of social robot → "The fear that robots will cause ICs to withdraw their care is one that should be taken seriously. The withdrawal of care by ICs could have the effect of shifting the burden onto FCs, or of reducing the overall degree of care the older person receives, or both. This displacement may foster resentment amongst informal carers, which may impact negatively on the older person's care." (Jenkins and Draper 2015 Care, Monitoring, and Companionship: Views on Care Robots from Older People and Their Carer, p. 679)
    - *Cont. on next page*
  - *Cont. on next page*
- *Cont. on next page*

Ethical hazards for care recipients (continued)

- Reducing/not adequately securing well-being/health (continued)
  - Reduction in the quality of care provided (continued)
    - Improper delegation of tasks to social robot → "'The lack of human resources must not lead to technology being seen as the solution. Technology is only as good as those who deploy and use it. Anyone who uses cuddly robots to sedate dementia patients left alone has not understood their profession' (geriatric nurse, female, aged 53)." (Translation by the authors)(Zöllick et al. 2020 Technik und Pflege – eine ambivalente Beziehung, p. 51)
    - Social robots are not fit to meet care tasks they are designed for → "Others don't see technology as being able to provide social and emotional support: 'No robot can understand the subtle nuances of words, tone of voice and dialect, the connotations in communication. They are mixed with gestures, facial expressions, environment, situation and culture and are not always easy for humans to understand. A robot can't do that. You can't touch with plastic'. (Geriatric nurse, female, 53 years old)." (Translation by the authors)(Zöllick et al. 2020 Technik und Pflege – eine ambivalente Beziehung, p. 51)
    - Social robots can't replace a "trustful care relationship" → "Care robots thus cannot be considered to replace caregivers in their function as a person trusted."(Wiertz 2020 Trusting robots? – On the concept of trust and on forms of human vulnerability, p. 65; in: Haltaufderheide et al. 2020 Aging between Participation and Simulation - Ethical Dimensions of Social Assistive Technologies)
    - Social robots don't have the same wealth of experience as nursing staff → "All these developments are associated with benefits such as physical and cognitive relief for care staff, but also risks such as the loss of experience-saturated judgment and relationship work based on this, among other things." (Translation by the authors)(Remmers 2019 Pflege und Technik. Stand der Diskussion und zentrale ethische Fragen, p. 413)
    - Reduced sensitivity/empathy of care providers due to social robots → "Artificial systems therefore have a particularly negative impact on the development of the virtues of care staff and their relationship with those in need of care. For example, the sensitivity, empathy, commitment and personal development of care staff suffer from the use of artificial systems." (Translation by the authors)(Misselhorn 2018 Pflegesysteme, p. 153; in: Misselhorn 2018 Grundfragen der Maschinenethik)
    - Being a testbed for social robots → "A study commissioned by the European Commission (Butter et el., 2008) on the use of robotics in healthcare discusses the following ethical problems, which are also relevant to the care of the elderly: [...] the use of ill and vulnerable people for experimental purposes; [...]." (Translation by the authors)(Bioethikkommission beim Bundeskanzleramt Österreich 2018 Roboter in der Betreuung alter Menschen (Stellungnahme), pp. 363, 364)
  - *Cont. on next page*
- *Cont. on next page*

- Ethical hazards for care recipients (continued)
  - Reducing/not adequately securing well-being/health (continued)
    - Adverse effect on emotional/psychological well-being → "In some cases, interventions with PARO caused negative emotional responses, including anger, wandering, fearfulness, and agitation [17, 20, 35]." (Hung et al. 2019 The benefits of and barriers to using a social robot PARO in care settings: a scoping review, p. 6)
      - Feeling uncomfortable with social robot → "Companion robots are designed to counter social isolation and loneliness. Most people, however, feel uncomfortable with such robots [...]."(Kemenade et al. 2019 Do You Care for Robots That Care? Exploring the Opinions of Vocational Care Students on the Use of Healthcare Robots, p. 2)
      - Enhancing feelings of isolation/loneliness → "In this regards, it is plausible to assume that humans will not only stay lonely when someone places a robot in their home, but that this allows them realizing even more that in fact no-body cares about them (which is the reason for being lonely in the first place). Thus, supposedly being cared about by a robot might serve as the ultimate proof for really being lonely."(von Maur 2023 Alice Does not Care – Or: Wy it Matters That Robots "Don't Give a Damn", p. 224; in: Loh und Loh 2023 Social Robotics and the Good Life - The Normative Side of Forming Emotional Bonds With Robots)
      - User interface is experienced as overwhelming/hard to control → "In addition to their curiosity about the system, some of the people with dementia had reservations about using a telepresence robot. For them, the use of this technology - often in contrast to their relatives - did not always seem comprehensible, even when the device was demonstrated to them 'in use' in their own home. In some cases, people with dementia specifically stated that they were 'overwhelmed' or 'unable to cope' with operating the telepresence robot." (Translation by the authors)(Bleses and Dammert 2020 Neue Technologien aus Sicht der Pflegewissenschaft, p. 69; in: Hanika 2020 Künstliche Intelligenz, Robotik und autonome Systeme in der Gesundheitsversorgung)
      - Feeling of loss/jealousy when robot is away, broken or shared → "The robot, however, may have to be shared with others – other residents in a care home, for instance – or break down, [...] leading at times to feelings of jealousy, disillusionment or disappointment."(Battistuzzi et al. 2018 Embedding Ethics in the Design of Culturally Competent Socially Assistive Robots, p. 1998)
      - Being disappointed/frustrated because expectation of robot not met → "We observed that Henry, when he was first deployed at the care site was often expected to be able to engage in natural conversation and to understand human language. As this was not the case, people were quickly frustrated by failing to communicate with the robot."(Körtner 2016 Ethical challenges in the use of social service robots for elderly people, p. 304)
      - Increased stress level (e. g. due to malfunction/reduced contact) → "The robot's absence may produce distress and/or loss of therapeutic benefits. This consequence can occur especially in users who cannot understand the reason of the robot removal." (Tzafestas 2016 Socialised Roboethics, p. 119; in: Tzafestas 2016 Roboethics - A Navigating Overview)
      - *Cont. on next page*
    - *Cont. on next page*
  - *Cont. on next page*

Ethical hazards for care recipients (continued)

- Reducing/not adequately securing well-being/health (continued)
  - Adverse effect on emotional/psychological well-being
    - Suffering from conflicts with care providers about usage of social robot → "We will show that assisting older people and their carers may often not amount to the same thing, because there can be serious tensions between older people and their carers that can be exacerbated by the introduction of a robot." (Jenkins and Draper 2015 Care, Monitoring, and Companionship: Views on Care Robots from Older People and Their Carer, p. 673)
    - Feeling humiliated/disrespected by implied images of oneself → "In the next example from the beginning of the film, it becomes clear how much Frank initially resists the imposed robot: Hunter, the son, gets the robot out of the trunk of his car. [...] Frank: 'Are you kidding me? I'm not that bad off! I don't need to be spoon-fed by a goddamn robot!' Hunter: 'Dad, Dad, no one says that either! He's new... He's more like a, eh, something like a butler!'" (Translation by the authors)(Frebel 2015 Roboter gegen das Vergessen? - Technische Assistenz bei Altersdemenz im Spielfilm aus medizinethischer Sicht, p. 112)
    - Being ashamed/scared of how others view them when needing social robot → "Some participants described interacting with robots as potentially shameful or embarrassing, signifying to others that they may be experiencing cognitive decline. This represented a threat to a person's self-esteem and sense of identity and even a loss of control over one's own life (e.g., in the case of the individual fearing she would be removed from her own home)." (Coghlan et al. 2021 Dignity, Autonomy, and Style of Company: Dimensions Older Adults Consider for Robot Companions, p. 16)
    - Further negative feelings → "Some may be confused by the SAR, or have difficulty understanding how it works, what to do to make it work or what triggers its actions, which could lead to self-blame and lowered self-efficacy." (Battistuzzi et al. 2021 Socially Assistive Robots, Older Adults and Research Ethics: The Case for Case-Based Ethics Training, p. 650)
  - Adverse effects on safety/physical well-being → "Safe and responsible introduction of social robots to this target group is essential, as a potential lack of knowledge of and experience with new technologies may lead to situations that potentially affect psychological and/or physical safety (Borenstein et al., 2017)". (Van Maris et al. 2020 Designing Ethical Social Robots – A Longitudinal Field Study With Older Adults, p. 2)
    - Harms caused by limited control/overtrust in social robot → "This then can be a problem of truthfulness, but also a source of practical misunderstandings and the dangers that arise from them: if users are not able to tell what exactly the machine is capable of, they might be led to assume that the machine has understood a command that, in reality, it hasn't; for example the command to remind the patient to take medication. If the patient relies on the machine to perform the task, a dangerous situation can arise, in which the machine's failure to clearly advertise its capabilities and limitations can directly lead to harm." (Matthias 2015 Robot Lies in Health Care: When Is Deception Morally Permissible?, pp. 170, 171)
    - *Cont. on next page*
  - *Cont. on next page*
- *Cont. on next page*

Ethical hazards for care recipients (continued)

- Reducing/not adequately securing well-being/health (continued)
  - Adverse effects on safety/physical well-being
    - Harms caused by malfunction → "In an aged care setting where robots interact in close, direct contact with the elderly, any malfunctioning can result in a disastrous outcome."(Poulsen et al. 2020 Cybersecurity, value sensing robots for LGBTIQ+ elderly, and the need for revised codes of conduct, p. 2)
    - Harms caused by social robot allowed to learn/make own decisions → "Suppose the care facility uses a brand-new robotic system that has been designed by the manufacturer to be as safe as possible, tested many times and also equipped with self-learning functions that should enable the system to adapt to the special needs and sensitivities of each individual patient as optimally as possible. [...] Hacker H manages to hack the hospital network and manipulate the system's learning process in such a way that the machine learns to injure patients." (Translation by the authors)(Hilgendorf 2018 Recht und Ethik in der Pflegerobotik – ein Überblick, p. 380)
    - Harms caused by accidents where social robots and humans live together → "Of course it is important to make sure the robot does not become an additional fall risk for the users."(Wiczorek et al. 2020 Domestic Robots for Older Adults: Design Approaches and Recommendations, p. 207; in: Woodcock et al. 2020 Design of Assistive Technology for Ageing Populations)
    - Harms caused by hygiene problems with social robot → "Studies highlighted that it can be difficult to keep PARO's fur clean [11, 38], and that the fur covering is not designed to be regularly removed or machine washed, which may post a concern especially to individuals who are immunocompromised [3, 22]." (Hung et al. 2019 The benefits of and barriers to using a social robot PARO in a care setting: a scoping review, p. 6)
- Infringing the right to privacy → "Health robots and monitoring can entail a deterioration of an elderly person's quality of life and a disproportionate invasion of their privacy." (The Swedish National Council on Medical Ethics 2015 Robots and Surveillance in Health Care of the Elderly – Ethical Aspects, p. 447)
  - Limited capacity of social robot to understand what should (not) be shared → "When discussing authority with respect to SAR, privacy is of utmost importance. A robot might not have sufficient capabilities to distinguish between privileged information and information that can be distributed. A robot may also lack the ability to distinguish between individuals who have the authority to receive information about the user and those who do not."(Feil-Seifer and Mataric 2011 Socially Assistive Robotics - Ethical Issues Related to Technology, p. 29)
    - *Cont. on next page*
  - *Cont. on next page*
- *Cont. on next page*

- Ethical hazards for care recipients (continued)
  - Infringing the right to privacy (continued)
    - Limited capacity of social robot to understand what should (not) be shared (continued)
      - Sharing personal information aloud in public places → "As the user may prefer not to share the content of these messages with other people in the room, carrying out this type of tasks [reading out messages aloud, addition by the authors] should be explicitly required or authorized by users."(Battistuzzi et al. 2018 Embedding Ethics in the Design of Culturally Competent Socially Assistive Robots, p. 2001)
    - Limited possibilities to control data recording/sharing → "Our second concern is that the elderly care receivers may lose control of their data monitored by the robot." (Li et al. 2020 The Complexity of Autonomy: A Consideration of the Impacts of Care Robots on the Autonomy of Elderly Care Receivers, p. 322; in: Nørskov et al. 2020 Frontiers in Artificial Intelligence and Applications)
      - Social robot records/operates on/shares distorted data → "Such a robotic system interacts with the user to learn the user's preferences, with the aim of providing a better service, and the user or any authorised users should be able to interact with the robotic system locally or remotely. In the process, this poses several unanswered questions pertaining to users' privacy and data security: What if the input data is manipulated? What if the stored data is either visible to, or corrupted or tampered with by an unauthorised user? What if the sensor data is fraudulent?" (Marchang and Di Nuovo 2022 Assistive Multimodal Robotic System (AMRSys): Security and Privacy Issues, Challenges, and Possible Solutions, p. 2)
    - Data collection or virtual connection in moments of intimacy → "An operator could drive a robot to peer round an elder's apartment before they were dressed or when they are taking a bath. An autonomous robot could record in the same circumstances."(Sharkey and Sharkey 2014 The Rights and Wrongs of Robot Care, p. 271; in: Lin et al. 2012 Robot Ethics: The Ethical and Social Implications of Robotics)
    - Feeling of being constantly controlled/followed by social robot → "The other concern relative to the robot's proactivity is the discomfort caused by the feeling of being permanently observed by a machine." (Gelin 2017 The Domestic Robot: Ethical and Technical Concerns, p. 210; in: Ferreira et al. 2017 A World with Robots - Intelligent Systems, Control and Automation: Science and Engineering)
    - Not being aware that data is collected → "A person with Alzheimer's may soon forget that a robot is present and might perform acts or say things believing he is in the privacy of his own home, or thinking that he is alone with his robot friend."(Sharkey and Sharkey 2014 The Rights and Wrongs of Robot Care, p. 271; in: Lin et al. 2012 Robot Ethics: The Ethical and Social Implications of Robotics)
    - *Cont. on next page*
  - *Cont. on next page*

- Ethical hazards for care recipients (continued)
  - Infringing the right to privacy (continued)
    - Data security/improper access to data → "Furthermore, intimacy and privacy and the related informational autonomy are important issues. Robots usually have cameras and sensors of all kinds, especially when they are mobile, and can thus, in principle, photograph and monitor patients in need of care. Personal data in this area is very sensitive and must be protected. Self-learning systems pose further problems. The AI-enhanced robot could create detailed profiles that would be interesting for nefarious groups, for example for coercion and extortion." (Bendel 2020 Care Robots with Sexual Assistance Functions, p. 5)
  - Promoting discrimination/negative images of care recipient → "Past research indicates that having a robot that looks like a toy such as My Real Baby (a robotic doll that becomes happy if you bounce it but gets distraught if you bounce it too much) may be stigmatising, patronising and infantilizing." (Frennert and Östlund 2014 Review: Seven Matters of Concern of Social Robots and Older People, p. 306)
    - Social robot might discriminate certain groups (e. g. hearing impaired) → "Another conceptual study examining various legal-ethical issues of mobile servant robots raised the possibility of robots discriminating against older people, especially those with speech and hearing impairments who could not actively summon the robots to assist them (Fosch-Villaronga and Virk, 2017)." (Tan et al. 2021 Tensions and antagonistic interactions of risks and ethics of using robotics and autonomous systems in long-term care, p. 10)
    - Furthering image of care recipient as dependent → "Ethical issues included the [...] patients' stigmatisation as being frail and dependent when using robotic devices,25, 28, 32 [...] ." (Servaty et al. 2020 Implementation of robotic devices in nursing care. Barriers and facilitators: an integrative review, p. 9)
    - Furthering objectification of care recipients → "Regarding social robots in care, the objectification of older people (Decker, 2008; Sparrow & Sparrow, 2006) might occur when residents are provided with robots due to the needs of the care organization or other people instead of the older people themselves." (Pirhonen et al. 2020 Can robots tackle late-life loneliness? Scanning of future opportunities and challenges in assisted living facilities, p. 9)
    - Further infantilization and deficit-orientation → "Similarly, interaction with toy robots will have the effects of infantilizing old people." (Noori et al. 2019 Robot-Care for the Older People: Ethically Justified or Not?, p. 46)
  - Increasing dependency in everyday life → "Finally, scholars point out that dependence and lack of competencies can be consequences of robots that provide too much help or assistance." (Salvini 2015 On Ethical, Legal and Social Issues of Care Robots, p. 440; in: Mohammed 2015 Intelligent Assistive Robots - Recent Advances in Assistive Robotics for Everyday Activities)
    - *Cont. on next page*
  - *Cont. on next page*

Ethical hazards for care recipients (continued)

- Increasing dependency in everyday life (continued)
  - Accelerate admission to care homes → "A tangible fear was that allowing others, including robots, to take over certain tasks and activities could result in a deterioration of mental function and the erosion of independence, potentially representing a step on the road to residential aged care."(Coghlan et al. 2021 Dignity, Autonomy, and Style of Company: Dimensions Older Adults Consider for Robot Companions, p. 16)
  - Becoming dependent on technology → "After receiving excessive assistance by delegating tasks to robots, the care receivers may become so dependent on robots concerning daily tasks they could have done by themselves that this gradually leads to deterioration of bodily functions [. . . ]."(Li et al. 2020 The Complexity of Autonomy: A Consideration of the Impacts of Care Robots on the Autonomy of Elderly Care Receivers, p. 323; in: Nørskov et al. 2020 Frontiers in Artificial Intelligence and Applications)
    - Emotional dependency → "Attachment to and dependency on the robot: Robot users may get emotionally attached to their robots. While such attachment is desirable for increasing robot acceptance, it also has the potential to be emotionally harmful [29, 33]."(Casey et al. 2016 What People with Dementia Want: Designing MARIO an Acceptable Robot Companion, p. 321)
    - Dependent on maintenance and support → "We are vulnerable whenever our assistive device does not fulfill its function and in regard to this vulnerability we are dependent on the people responsible for the functioning and maintenance of our assistive device."(Wiertz 2020 Trusting robots? – On the concept of trust and on forms of human vulnerability, p. 64; in: Haltaufderheide et al. 2020 Aging between Participation and Simulation - Ethical Dimensions of Social Assistive Technologies)
  - Causing loss of further capacities (as not practiced anymore) → "Some of the elders inquired have expressed their concern that an excessively prestative robot could lead seniors to rely on robots for everything, even for the tasks they could perform well causing them to lose more quickly their remaining physical abilities [. . . ]."(Gelin 2017 The Domestic Robot: Ethical and Technical Concerns, pp. 211, 212; aus Ferreira et al. 2017 A World with Robots - Intelligent Systems, Control and Automation: Science and Engineering)
- Creating barriers for autonomous and informed decision-making → "The use of robots in care facilities must also be viewed critically, as many residents are no longer in a position to give their voluntary and informed consent to such measures, for example because they suffer from mental impairments." (Translation by the authors)(Misselhorn 2018 Pflegesysteme, p. 153; in: Misselhorn 2018 Grundfragen der Maschinenethik)
  - Social robot is designed in a way that limits choices/control → "A focus group study [20] in Belgium shows that elderly care receivers worry if they may partly lose their autonomy when robots are used in care. They insist that they should be able to turn off the robots when robots infringe on their autonomy."(Li et al. 2020 The Complexity of Autonomy: A Consideration of the Impacts of Care Robots on the Autonomy of Elderly Care Receivers, p. 318; in: Nørskov et al. 2020 Frontiers in Artificial Intelligence and Applications)
  - *Cont. on next page*
- *Cont. on next page*

Ethical hazards for care recipients (continued)

- Creating barriers for autonomous and informed decision-making (continued)
  - Exposure to manipulation or feeling pressured to act by social robot → "That is, the social nature of the interaction depends crucially on the user being tricked or fooled into ascribing emotional states to the robot seal which do not pertain to it. There is an emotional manipulation of the user by the designer of the robot.11 This is an offense against the person's rational nature, since the workings of this mechanism depend on the suspension of rational reflection and control." (Misselhorn et al. 2013 Ethical Considerations Regarding the Use of Social Robots in the Fourth Age, p. 129)
  - Improper delegation of decision-making authority to social robot → "Such loss of freedom is related to the issue of autonomy raised in the preceding section. If robots are to be used to help elderly people to live safely in their own homes for longer, there is likely to be a need for them to function as autonomous supervisors. A simple extension would be to allow home customisation so that the robot can recognise danger signs, such as the cooker being left on, or the bath overflowing. A robot could make a first pass at warning a senior to stop doing or engaging in a potentially dangerous activity. But there are ethical problems here. If a robot can predict a dangerous situation, it could be programmed to autonomously take steps to prevent it, e.g. turning the cooker off, or even restraining the elderly person (gently) from carrying out a potentially dangerous action such as climbing up on a chair to get something from a cupboard. However, restraining a person to avoid harm could be a slippery slope towards authoritarian robotics." (Sharkey and Sharkey 2012 Granny and the robots: ethical issues in robot care for the elderly, p. 33)
  - Care recipients might be pressured into using social robot → "Similar external pressures should also be prevented at the level of domestic or residential use of robots. In particular, scenarios where family members or other informal caregivers force a patient with capacity to consent to have a service robot in the house—e.g. because they want to reduce their time-investment and caregiving-workload should be prevented." (Ienca et al. 2016 Social and Assistive Robotics in Dementia Care: Ethical Recommendations for Research and Practice, p. 568)
  - Care recipients might not be adequately informed about social robots → "Finally, the authority that explains the robot and conveys the allegedly neutral information assumes a position of power. This creates a danger of manipulation of the users. The person who explains the robot can decide to share the information they feel is important about the robot and withhold other information." (Weßel et al. 2021 Gender Stereotyping of Robotic Systems in Elderly Care An Exploratory Analysis of Ethical Problems and Possible Solutions, p. 7)
    - Social robots add an extra layer of complexity → "PARO was found to not have the option to easily turn off because its hidden switch between the split tail fins; older people with dementia did not know how to turn the robot on or off [43]." (Hung et al. 2019 The benefits of and barriers to using a social robot PARO in a care setting: a scoping review, p. 7)
    - KI-informed care approaches stay opaque → "…the technological complexity of several IATs makes it harder for patients and/or their proxy decision makers (e.g. caregivers) to adequately understand what they are consenting to." (Wangmo et al. 2019 Ethical concerns with the use of intelligent assistive technology: findings from a qualitative study with professional stakeholders, p. 8)
  - *Cont. on next page*
- *Cont. on next page*

Ethical hazards for care recipients (continued)

- Creating barriers for autonomous and informed decision-making (continued)
  - Design (e. g. anthropomorphic/female) leads to over-trust → "For instance, deception could lead to over-trust the robot –thus endangering the user's safety or reasonable decision-making processes–, [. . . ]."(Boada et al. 2021 The ethical issues of social assistive robotics: A critical literature review, p. 6)
- Increasing social isolation → "Another argument put forward against the use of Paro is that, contrary to its intended use, Paro could cause or promote isolation if it were to replace, rather than supplement, human contact." (Translation by the authors)(Lange and Bauer 2021 - Eine Robbe für Oma - Die zukünftige Dauerausstellung Robotik im Deutschen Museum, p. 232; in: Inthorn und Seising 2021 Digitale Patientenversorgung - Zur Computerisierung von Diagnostik, Therapie und Pflege)
  - Social robot fosters social seclusion of care recipient (via dependent relation) → "While many studies support the idea of a preference for human-versus-robotic interaction, De Graaf57 expressed the concern that the human–robot relationship may replace their human counter-part in social relations and predicted that if people share feelings with robotic others, they may become accustomed to the reduced emotional range that these machines can offer."(Diaz-Orueta et al. 2020 Shaping technologies for older adults with dementia: Reflection on ethics and preferences, p. 3222)
  - Reduction of physical visits → "There is evidence that robots can reduce visits by care staff at home (Broadbent 2017)." (Translation by the authors)(Bleuler and Caroni 2021 Roboter in der Pflege - Welche Aufgaben können Roboter heute schon übernehmen?, p. 445; in: Bendel 2021 Soziale Roboter)
    - Uncomfortable feelings around social robot discourage visits → "N=15 would not like being received by a service robot as a relative." (Translation by the authors)(Meyer 2011 Akzeptanz ausgewählter Anwendungsszenarien, p. 96; in: Meyer 2011 Mein Freund der Roboter - Servicerobotik für ältere Menschen - eine Antwort auf den demographischen Wandel?)
    - Don't worry about Granny, she's got the social robot to talk to → "The robot might indeed be a good excuse to cancel some of Anna's visits, as she feels less guilty because her grandmother has a 'companionship'." (Felber et al. 2022 The concept of social dignity as a yardstick to delimit ethical use of robotic assistance in the care of older persons, p. 105)
    - Less control visits necessary and therefore visitors → "If care robots can be used to monitor the activities of daily living of the older people by their dependents who live apart, less social visits could be an unintended consequence in the process of leveraging the technology."(Tan et al. 2021 Tensions and antagonistic interactions of risks and ethics of using robotics and autonomous systems in long-term care, p. 11)
    - *Cont. on next page*
  - *Cont. on next page*
- *Cont. on next page*

Ethical hazards for care recipients (continued)

- Increasing social isolation (continued)
  - Reduction of physical visits (continued)
    - Physical visits are substituted by virtual visits → "For example, maybe elderly care receivers will not see their family and friends as frequently when they can virtually meet via the robot." (Li et al. 2020 The Complexity of Autonomy: A Consideration of the Impacts of Care Robots on the Autonomy of Elderly Care Receivers, p. 322; in: Nørskov et al. 2020 Frontiers in Artificial Intelligence and Applications)
  - Reduction of human attention/contact resp. "real"/emotional component of care in care relationships → "The concern that humanity could be reduced in care and nursing is nevertheless not entirely unjustified. On the one hand, working with the robotic seal Paro actually shows that people with dementia no longer need much additional care during the time with Paro on their lap because they are busy with the seal, and on the other hand, carers - especially in hospitals - are usually tied to a tight schedule that leaves little room for activity and conversation (Newerla 2017)." (Translation by the authors)(Schmidhuber 2022 Werden Roboter Menschen in der Pflege ersetzen? Ethische Überlegungen, pp. 169, 170; in: Stronegger und Platzer 2022 Technisierung der Pflege: 4. Goldegger Dialogforum Mensch und Endlichkeit)
    - (Sex)robots can't give the same love like humans → "Jecker's illusions about sex robots originate from a failure to recognise that what 'sex' robots facilitate is masturbation, not intercourse. Sex robots are sex toys, not partners."(Sparrow 2021 Sex robot fantasies, p. 33)
  - Promotes distance from non-robot-using humans (and vice versa) → "Of course at the same time, the robot may encourage people to disengage from the non-robotic environment and to take distance from others who are not involved in the robotic adventure. There is likely to be a distance between those who interact with the robot and talk about among themselves, and those who are excluded from the interaction and robot-mediated and robot-focused conversation."(Coeckelbergh 2016 Care robots and the future of ICT-mediated elderly care: a response to doom scenarios, p. 459)
- Care not tailored to varying needs → "One participant (male) would not want to use the robot at all. This participant stated that, although he liked interacting with the robot, he found that it was not sufficient for his needs as he preferred a robot that was capable of physical assistance."(Van Maris et al. 2020 Designing Ethical Social Robots – A Longitudinal Field Study With Older Adults, p. 10)
  - Developed/designed based on stereotypes/images that ... → "Sex robots and love dolls can certainly be used in old people's and nursing homes. However, there are several points against this: [. . . ] They partly correspond to stereotypes that are perceived differently and sometimes rejected."(Bendel 2020 Care Robots with Sexual Assistance Functions, p. 4)
    - *Cont. on next page*
  - *Cont. on next page*
- *Cont. on next page*

- Ethical hazards for care recipients (continued)
  - Care not tailored to varying needs (continued)
    - Developed/designed based on stereotypes/images that ... (continued)
      - Not adequately consider diversity of lifestyles → "Moreover, there is often an imbalance between perceptions of older peoples' technology needs and knowledge about their actual needs. The supposed user employs the technology according to the manufacturer's idea of how the item should be used. However, this is distinct from the real user, who is actually using the technology, and may for instance change the purpose of the technology (Dekker 2015). If diversity in users is incorporated at all, it is most often based on basic social distinctions such as age and gender differences (Flandorfer 2012)."(Hoppe et al. 2020 Assistive robots in care: Expectations and perceptions of older people, p. 144; in: Haltaufderheide et al. 2020 Aging between Participation and Simulation - Ethical Dimensions of Social Assistive Technologies)
      - Not adequately address needs of targeted population → "Sexbots in their current form are generally designed for able-bodied heterosexual men, and are therefore not well-suited to providing sexual pleasure for frail, elderly people – most of whom are women – with limited dexterity." (Lancaster 2023 Granny and the Sexbots: An ethical appraisal of the use of sexbots in residential care institutions for elderly people, p. 203; in: Loh und Loh 2023 Social Robotics and the Good Life - The Normative Side of Forming Emotional Bonds With Robots)
    - Care not provided culturally-sensitive → "All forms of care—including SAR use—need to be sensitive to personal and cultural differences in experiences (Coeckelbergh, 2010; Körtner, 2016; Parks, 2010; Sorell & Draper, 2014). This sensitivity must be expanded to include not only differences in care experiences of today's older adults, but also those of tomorrow (Misselhorn et al., 2013; Sorell & Draper, 2014)." (Vandemeulebroucke et al. 2021 Socially Assistive Robots in Aged Care: Ethical Orientations Beyond the Care-Romantic and Technology-Deterministic Gaze, p. 17)
    - Social robot cannot tailor support to individually varying needs → "The now famous robotic seal 'Paro' is also a joyful change for some people with dementia because it is soft, can sit on the lap and reacts to being touched with noises and a glance. However, people who don't like animals often have little use for this form of activity." (Translation by the authors)(Schmidhuber and Stöger 2021 Ethisches und Rechtliches zur Zukunft der Robotik in der Pflege. Grundfragen für österreichische und deutsche Debatten, p. 95)
      - Care approach based on learning model that restricts options → "Although the system has some learning capacity, Anderson and Anderson's approach is therefore blind to cases in which the specific needs and moral values of individual care recipients become relevant." (Translation by the authors) (Misselhorn 2019 Moralische Maschinen in der Pflege? Grundlagen und eine Roadmap für ein moralisch lernfähiges Altenpflegesystem, p. 59; in: Woopen und Jannes 2019 Roboter in der Gesellschaft – Technische Möglichkeiten und menschliche Verantwortung)
      - *Cont. on next page*
    - *Cont. on next page*
  - *Cont. on next page*

Ethical hazards for care recipients (continued)

- Care not tailored to varying needs (continued)
  - Social robot cannot tailor support to individually varying needs (continued)
    - Rule-based/reproducible action clashes with tailored approaches → “It is particularly feared that technical systems that function with situation-invariant regularity will contradict the individualization and contextualization of care activities that are essential for good care.” (Translation by the authors)(Deutscher Ethikrat 2020 Robotik für gute Pflege (Stellungnahme), p. 43)
  - Care providers are less attuned to the individual needs of care recipients because of social robot → “Critics fear, perhaps justifiably, that caregivers might become less attuned to the specific needs of care recipients because a technological crutch is available.”(Borenstein and Pearson 2012 Robot Caregivers: Ethical Issues across the Human Lifespan, p. 260; in: Lin et al. 2012 Robot Ethics: The Ethical and Social Implications of Robotics)
- Deception about relationships/personal bonds that do not exist → ”However, it was also demonstrated that some users formed attachments and emotional bonds with robots they were interacting with. These attachments led to misconceptions about the robots’ emotional capabilities. For example, one user felt that the robot would miss him when he was gone, something that the robot was not capable of doing.” (Matari? and Scassellati 2016 Socially Assistive Robotics, p. 1987; in: Siciliano und Khatib 2016 Springer Handbook of Robotics)
  - Impoverishment of one’s life by illusioned relations → “For instance, linking dignity to the capabilities approach, one perspective in this vein argues to assess SAR depending on whether a robot’s use expands or restricts the access to the basic set of capabilities to live a worthy human life. Also, dignity is under attack when these unauthentic intersubjective interactions alienate users from real life, thereby impoverishing their world.”(Boada et al. 2021 The ethical issues of social assistive robotics: A critical literature review, p. 7)
- Loss of trust in care providers (including social robot carer) → “Hijacking a companion robot could lead unintended users to vocalize offensive, anti-LGBTIQ+ language, which could seriously endanger the trust the user put into the system.”(Poulsen et al. 2020 Cybersecurity, value sensing robots for LGBTIQ+ elderly, and the need for revised codes of conduct, p. 4)
  - Loss of trust due to inadequate design of social robot → “(Some people may) ‘Lose trust in the system if the robot goes behind their back ... blackmailing the patient. Depends whether the person really had taken the medication (human intervention that the robot was not aware of) [. . . ].’ (Robotics academic)”(Poulsen and Burmeister 2019 Overcoming carer shortages with care robots: Dynamic value trade-offs in run-time, pp. 9, 10)
  - *Cont. on next page*
- *Cont. on next page*

Ethical hazards for care recipients (continued)

- Loss of trust in care providers (including social robot carer) (continued)
  - Loss of trust in care provider through reduced physical interaction → "Conventional physical interaction, which often conveys calm and builds trust, could be omitted when using technology." (Translation by the authors)(Deutscher Ethikrat 2020 Robotik für gute Pflege (Stellungnahme), p. 26)
- Beneficial social robots not/no longer put to use → "Some studies described situations where PARO appeared ineffective for some individuals or lost effect over time [34, 41]." (Hung et al. 2019 The benefits of and barriers to using a social robot PARO in care setting: a scoping review, p. 7)
  - Reception of social robot might vary over time (reduction in interest) → "It was noted that when using PARO, staff should uphold a person-centred approach, as just because the resident liked PARO 1 day does not mean that he or she will enjoy it the next [26]." (Hung et al. 2019 The benefits of and barriers to using a social robot PARO in a care setting: a scoping review, p. 7)
  - Lack of acceptance prevents usage → "In order to be accepted, social robots need to be perceived by users as useful and relevant to their current unmet needs [15–19]. A mismatch between needs and the solutions offered by robots are a barrier to acceptance and robot adoption."(Casey et al. 2016 What People with Dementia Want: Designing MARIO an Acceptable Robot Companion, p. 319)
  - Costs of social robots prevent usage → "[. . . ] each unit of the Justocat costs about US $1350, an AIBO dog costs US $3000, and a Paro costs approximately US $6000. Cost and affordability can therefore influence equal access to such innovations by older adults and people with dementia [25]." (Koh et al. 2021 Impacts of Low-cost Robotic Pets for Older Adults and People with Dementia: Scoping Review, p. 2)
    - Environment of care recipient needs to be adapted to social robot (cost-intensive) → "Due to its size, the robot pictured in the scenario would not be able to operate in the homes of many older adults as they tend to have lots of furniture, knickknacks and rugs in their homes. The professional caregivers indicated it would also be challenging for the robot to operate in the houses of older adults on account of doorsteps, doors and small spaces (especially the kitchen, which is often small). Therefore, it was felt, the homes of older adults should be adapted to the robot. Some professional caregivers thought this would become an issue, as they thought many older adults would be unwilling to do so (thought the elderly participants stated they would not mind adapting their homes to the robot). Professional caregivers also wanted the robot to have its own place in the home and not to follow the user around everywhere. The robot's place, they opined, should be chosen cooperatively with the user."(Bedaf et al. 2019 What are the preferred characteristics of a service robot for the elderly? A multi-country focus group study with older adults and caregivers, p. 9)
  - *Cont. on next page*
- *Cont. on next page*

- Ethical hazards for care recipients (continued)
  - Beneficial social robots not/no longer put to use (continued)
    - Further points that prevent use → "And which technical systems can be used also depends on their living conditions. Humanoid robots and other (self-)driving assistance systems need room to move." (Translation by the authors)(Hübner and Müller 2020 Roboter in der Pflege, p. 6)
  - Lack of protection due to lack of guidelines/legal regulations → "The lack of agreed standardized procedures and guidelines for therapeutic robots impedes the establishment of a safeguard baseline to be respected by anyone working in this area, and this leaves users unprotected."(Fosch-Villaronga and Albo-Canals 2019 "I'll take care of you," said the robot, p. 81)
    - Problems following from unresolved liability issues → "'What happens if the robot makes a mistake, e.?g. delivers the wrong medicine? Who is liable? Actually, it is obviously the caregivers in the case that they provide the wrong medicine. But if the robot gives a wrong drug, who is liable then?' (GER 2018)."(Hoppe et al. 2020 Assistive robots in care: Expectations and perceptions of older people, p. 148; in: Haltaufderheide et al. 2020 Aging between Participation and Simulation - Ethical Dimensions of Social Assistive Technologies)
      - Costs of false alarms have to be shouldered by care recipients → "For instance, there are no clear rules and guidelines to date in specifying which party should be held responsible should robots inflict harm on the older people (Chou et al., 2018). For example, in case of a false alarm, the care robot could call for an ambulance, but the cost to the hospital or insurer will most likely be borne by the person being cared for."(Tan et al. 2021 Tensions and antagonistic interactions of risks and ethics of using robotics and autonomous systems in long-term care, p. 8)
      - No compensation for harm realized by social robot → "Recently, ethical advisory bodies have increasingly emphasized that the use of robotics (as well as AI) must not lead to (cared for) people having to bear the damage caused to them by a (partially) autonomous system (Bioethikkommission, 2020)." (Translation by the authors)(Schmidhuber 2022 Werden Roboter Menschen in der Pflege ersetzen? Ethische Überlegungen, p. 171; in: Stronegger und Platzer 2022 Technisierung der Pflege: 4. Goldegger Dialogforum Mensch und Endlichkeit)

Ethical opportunities for care providers/facilities (including caring relatives) (Main category)

- Positively impacting care provider's health and well-being → "Care robots are receiving growing attention in elder care since their use has proven to have a positive impact, not only on older adults but also for those who work with them, including professional caregivers and relatives [19]."(Johansson-Pajala et al. 2020 Care Robot Orientation: What, Who and How? Potential Users' Perceptions, p. 1104)
  - Reduced risk of infection in care homes → "Lio differs from other service robots in that it has an arm that enables it to manipulate objects. It can open and close doors, but also pick up objects from the floor or take them out of a cupboard and hand them over. These functions are particularly useful in times of a pandemic, as they make it possible to limit contact between wards or between nursing staff and patients, thereby reducing the spread of pathogens." (Translation by the authors) (Bleuler and Caroni 2021 Roboter in der Pflege - Welche Aufgaben können Roboter heute schon übernehmen?, p. 451; in: Bendel 2021 Soziale Roboter)
  - Reduced risk of workload negatively affecting health → "It is well known that professional carers in geriatric care are under more psychological and physical strain than carers in other areas. High time pressure and stress, together with the physical consequences of work (especially back pain), lead to high staff turnover. Robotic systems can be used here in various ways to provide support and relieve the burden on caregivers and/or informal or formal caregivers." (Translation by the authors)(Wahl et al. 2021 Robotik und Altenpflege: Freund oder Feind?, p. 63)
  - Feeling of "being there" for care recipient despite distance (e. g. relatives) → "According to current heuristic assumptions, caregiving relatives seem to experience a sense of presence through the use of a telepresence robot." (Translation by the authors)(Bleses and Dammert 2020 Neue Technologien aus Sicht der Pflegewissenschaft, p. 71; in: Hanika 2020 Künstliche Intelligenz, Robotik und autonome Systeme in der Gesundheitsversorgung)
  - Increasing the feeling of safety → "Relatives report in interviews that it reassures them when they can see [thanks to the telepresence robot] that the elderly person is eating something and something 'right' or is wearing the emergency call button, for example." (Translation by the authors)(Bleses and Dammert 2020 Neue Technologien aus Sicht der Pflegewissenschaft, p. 72; in: Hanika 2020 Künstliche Intelligenz, Robotik und autonome Systeme in der Gesundheitsversorgung)
  - Reducing stress → "While Paro was originally developed for use by elderly residents of nursing homes, investigation has shown that it also has a positive effect on the carers who showed lower levels of stress and took fewer sick leaves when they worked in environments where Paro is used."(Lau et al. 2009 R&D in healthcare robotics, p. 29; in: Lau et al. 2009 Beyond the Surface – An Exploration in Healthcare Robotics in Japan)
  - *Cont. on next page*
- *Cont. on next page*

Ethical opportunities for care providers/facilities (including caring relatives) (continued)

- Positively impacting care provider's health and well-being (continued)
  - Less inhibitions in dealing with cognitive impaired people → "On the one hand, the robotic seal increased the willingness of the person in need of care to cooperate with the geriatric nurse, as communication between them was improved, and on the other hand, career changers in particular were able to overcome their own inhibitions in dealing with dementia patients more quickly by working with PARO." (Translation by the authors)(Scorna 2015 Servicerobotik in der Altenpflege – Eine empirische Untersuchung des Einsatzes der Serviceroboter in der stationären Altenpflege am Beispiel von PARO und Care-O-bot, p. 92; in: Weber et al. 2015 Technisierung des Alltags – Beitrag für ein gutes Leben?)
- Improving working conditions for care providers → "Another of the main arguments used to support the development of care robots is the need to improve the quality of care. [. . . ] Improving the quality of care it means providing better working conditions for care-givers [sic] [. . . ]."(Salvini 2015 On Ethical, Legal and Social Issues of Care Robots, p. 432; in: Mohammed 2015 Intelligent Assistive Robots - Recent Advances in Assistive Robotics for Everyday Activities)
  - Reducing workload → "For example, a robot that does something that a human caregiver would otherwise do (e.g., providing encouragement for performing exercises) might have as much impact on the human caregiver as on the patient, through the reduction of tasks related to a patient or through the reduction of workplace monotony. Specifically, many SAR systems are being designed to reduce the burden and burnout of family members and other caregivers."(Feil-Seifer and Matarić 2011 Socially Assistive Robotics - Ethical Issues Related to Technology, p. 27)
    - Freeing up time for social interaction with care recipients → "Lio relieves the nursing staff and saves time by taking over repetitive tasks. These are errands or long walks in the corridor to remind residents of activities and appointments. This frees up more time for the specialized staff to invest in interpersonal relationships." (Translation by the authors) (Wirth et al. 2020 Erfahrungen aus dem Einsatz von Assistenzrobotern für Menschen im Alter, p. 261; in: Buxbaum 2020 Mensch-Roboter-Kollaboration)
    - (Informal) caregivers have more personal time → "The AT also provided carers with additional personal time which was highly valued and, in many instances, helped create the balance between their own personal space and independence with that of staying connected with the person with dementia." (Sriram et al. 2019 Informal carers experience of assistive technology use in dementia care at home: a systematic review, p. 13)
  - Providing tools to ease fulfilling of complex tasks → "In these therapies, called robotherapies, the interactions and communications between a subject and an artificial complex interactive system are used for evaluation, diagnosis, prognosis or treatment [2, 36, 37]."(Fosch-Villaronga and Albo-Canals 2019 "I'll take care of you," said the robot, p. 80)
    - *Cont. on next page*
  - *Cont. on next page*
- *Cont. on next page*

Ethical opportunities for care providers/facilities (including caring relatives) (continued)

- Positively impacting care provider's health and well-being (continued)
  - Providing tools to ease fulfilling of complex tasks (continued)
    - Providing support for personnel in crisis situations → "P56 (operator of a care center) sees it similarly and considers robots to be useful support in emergencies: '... also to develop emergency skills. This means that if called upon in an emergency, such a system can help a very excited, perhaps still young and fresh employee to deal with a crisis situation, an emergency situation, in a professional manner, so to speak.'" (Translation by the authors)(Carros et al. 2022 Roboter als intelligente Assistenten in Betreuung und Pflege – Grenzen und Perspektiven im Praxiseinsatz, p. 805; in: Pfannstiel 2022 Künstliche Intelligenz im Gesundheitswesen)
    - Supporting in diagnosis (e. g. testing sight and hearing) → "Only a few respondents were more specific. For example, P22 (management consultant) noted that robotics could be used very well in the field of AI-supported (early) diagnostics: 'Or in the broadest sense also a bit of early diagnostics, so if the hearing deteriorates or the eyes deteriorate or something like that, that then either supporting or analyzing tasks are set in the interaction, where it can also go into the care diagnosis or medical diagnosis in the broadest sense.'" (Tranlsation by the authors) (Carros et al. 2022 Roboter als intelligente Assistenten in Betreuung und Pflege – Grenzen und Perspektiven im Praxiseinsatz, p. 805; in: Pfannstiel 2022 Künstliche Intelligenz im Gesundheitswesen)
    - Providing activities (esp. for cognitive impaired people) → "The expert interviews conducted on PARO showed that all interviewees initially expected the robotic seal to bring joy to the residents, activate them and improve their general well-being. In this context, one expert from the field of geriatric care emphasized that she saw the robotic seal as a special offer for residents who require a lot of care and are very restricted due to their need for care." (Translation by the authors)(Scorna 2015 Servicerobotik in der Altenpflege – Eine empirische Untersuchung des Einsatzes der Serviceroboter in der stationären Altenpflege am Beispiel von PARO und Care-O-bot, p. 89; in: Weber et al. 2015 Technisierung des Alltags – Beitrag für ein gutes Leben?)
    - Supporting therapeutical approaches → "Furthermore, Paro seems to fulfill its intended purpose. On average, the robot has been used regularly in care facilities for over a year and a half for its intended purpose as a therapeutic aid [...], which speaks for its success in the care context." (Translation by the authors)(Baisch et al. 2018 Emotionale Roboter im Pflegekontext - Empirische Analyse des bisherigen Einsatzes und der Wirkungen von Paro und Pleo, p. 22)
  - Better networking/coordination with care recipients and informal caregivers → "On the other hand, they should improve the networking of recipients, informal and professional helpers and thus ensure the coordination of needs-based care work in complex structures (cf. Hülsken-Giesler and Krings 2015; Hülsken-Giesler 2015)." (Translation by the authors) (Hülsken-Giesler and Daxberger 2018 Robotik in der Pflege aus pflegewissenschaftlicher Perspektive, p. 127; in: Bendel 2018 Pflegeroboter)
    - *Cont. on next page*
  - *Cont. on next page*
- *Cont. on next page*

Ethical opportunities for care providers/facilities (including caring relatives) (continued)

- Positively impacting care provider's health and well-being (continued)
  - Better networking/coordination with care recipients and informal caregivers (continued)
    - Virtual visits can save time and energy → "Telepresence robots, such as Giraff and VGo, have proven effective in providing remote monitoring of adults with dementia and enabling long-distance control or interaction between patients and caregivers, often in combination with telephony and long-range remote control." (Ienca et al. 2016 Social and Assistive Robotics in Dementia Care: Ethical Recommendations for Research and Practice, pp. 566, 567)
  - Easing access to relevant patient information (e. g. adherence) → "Two months ago, Ms. Koertig was given a care robot. [...] When the mobile device connects to the care service's sensor, it recognizes that Stefanie Zimmermann is in the house and travels to her independently to support her. The care planning for Ms. Koertig is stored in the robot along the care process. It provides the necessary information and ensures that Stefanie doesn't forget anything. This has worked well the last few times. The first step is to measure her blood sugar level, as Ms. Koertig takes medication that needs to be dosed correctly. The robot shows the values from the previous weeks on a display [...]." (Translation bei the authors)(Seefeldt and Hülsken-Giesler 2020 Pflegeethik und Robotik in der Pflege, p. 271)
  - Reducing difficult behavior of care recipients → "Since social interaction with dementia patients can also be very frustrating for the relatives, some of the burden on the relatives may be alleviated if the interaction with Paro improves the condition of the patient by increasing affective well-being and reducing stress."(Misselhorn et al. 2013 Ethical Considerations Regarding the Use of Social Robots in the Fourth Age, p. 127)
  - Easy maintenance of robot pets over real animals → "Paro is helping in the care of dementia sufferers in a much more controlled way than real live companion animals. Paro will not be hurt if his mistress flies into a rage. Paro is easily washed and will not die."(Bennett 2014 Robot Identity Assurance, p. 11)
- Positively implicates relationships → "The intervention of robot caregivers could improve family unity and other interpersonal relationships because they would not be tainted by our aversion to unpleasant tasks."(Borenstein and Pearson 2012 Robot Caregivers: Ethical Issues across the Human Lifespan, p. 261; in: Lin et al. 2012 Robot Ethics: The Ethical and Social Implications of Robotics)
- Upgrading nursing profession → "Instead of replacing people, Lio and P-Care enable targeted care for older people and act as an extension of care staff. By engaging with technology and using it in residential homes with the help of robotics, care professions are being upgraded and new occupational fields are being created." (Translation by the authors)(Früh and Gasser 2018 Erfahrungen aus dem Einsatz von Pflegerobotern für Menschen im Alter, p. 44; in: Bendel 2018 Pflegeroboter)
- *Cont. on next page*

Ethical opportunities for care providers/facilities (including caring relatives) (continued)

- Easing management for care facilities → "The robot was seen as an enabler to increase collaboration with families in facility care and improve family care, both which are general targets in the Finnish elderly care system nowadays."(Niemelä et al. 2021 Towards Ethical Guidelines of Using Telepresence Robots in Residential Care, p. 436)
  - Social robots as competitive advantage on the market → "Lio therefore brings additional value for both residents and care staff. At the same time, it enables institutions to stand out from others and secure a place for themselves in the competitive market (Früh and Gasser 2018)." (Translation by the authors)(Bleuler and Caroni 2021 Roboter in der Pflege - Welche Aufgaben können Roboter heute schon übernehmen?, p. 451; in: Bendel 2021 Soziale Roboter)
  - Social robots can work all day long (without negative behavior change) → "In a study where a humanoid robot Zora was piloted in elder care services, the care personnel noted—among other things—that unlike a human caregiver, the robot does not get tired, it always responds in a friendly way, and it repeats things over and over if needed."(Pirhonen et al. 2020 Can robots tackle late-life loneliness? Scanning of future opportunities and challenges in assisted living facilities, p. 4)
  - Controlling adherence of care personnel to treatment standards → "Some formal carers raised the issue of the robot being used to 'spy' on them, whilst other formal carers did seem willing to use the robot to check up on, as well as to reinforce, adherence to treatment regimes."(Draper and Sorell 2017 Ethical values and social care robots for older people: an international qualitative study, p. 67)
  - Reducing costs for care facilities → "Care costs can be reduced in the medium term by dividing the support activities into two categories. Demanding management and care functions are carried out by qualified employees, who can then concentrate on meaningful tasks. Repetitive routine and auxiliary functions that do not necessarily require human intervention will be carried out by intelligent personal robots." (Translation by the authors) (Früh and Gasser 2018 Erfahrungen aus dem Einsatz von Pflegerobotern für Menschen im Alter, p. 45; in: Bendel 2018 Pflegeroboter)

Ethical hazards for care providers/facilities (including caring relatives) (Main category)

- Adverse effect on health/well-being of care providers → "However, when introducing robotic systems, it must of course be ensured that they do not lead to further strain on care staff." (Translation by the authors)(Gräb-Schmidt and Stritzelberger 2018 Ethische Herausforderungen durch autonome Systeme und Robotik im Bereich Pflege, pp. 359, 360)
  - Endangering safety of care providers → "In contrast with robots for factories, assistive technologies such as SAR share space with people and therefore they have the potential to accidentally harm humans and damage facilities."(Koimizu 2019 Aged Care with Socially Assistive Robotics under Advance Care Planning, p. 34)
    - Harms caused by malfunction/lack of suitability — "One rehabilitation staff participant reported the clutter in the hallway and resident room confused the sensors of the robot, hindering optimal movement of the robot."(Hung et al. 2022 Technological risks and ethical implications of using robots in long-term care, p. 4)
  - Feeling uncomfortable with social robot/social robot usage of care recipients → "In addition to the geriatric nurses who were immediately enthusiastic, there were always those who would have rejected care with PARO. The reasons for this are often that there is no money for such investments and that people don't believe that PARO has a positive effect on the residents, but rather assume that they want to trick those in need of care with the seal." (Translation by the authors)(Scorna 2015 Servicerobotik in der Altenpflege – Eine empirische Untersuchung des Einsatzes der Serviceroboter in der stationären Altenpflege am Beispiel von PARO und Care-O-bot, p. 91; in: Weber et al. 2015 Technisierung des Alltags – Beitrag für ein gutes Leben?)
- Aggravate work situation for care providers → "The applications of robotics and autonomous systems in long-term care also have both positive and negative employment implications to the incumbent nursing and health workforce in the future (Khaksar et al., 2016; Stahl and Coeckelbergh 2016; Chou et al., 2018; Metzler et al., 2015; Pfadenhauer and Dukat 2015)."(Tan et al. 2021 Tensions and antagonistic interactions of risks and ethics of using robotics and autonomous systems in long-term care, p. 8)
  - Investing in new technologies instead of working conditions → "It is a question of societal priorities and demands – and the narratives and imaginaries making these intelligible in the first place – whether resources are spent for the development of technologies like care robots or for structurally making professional and private human care-giving possible, acknowledged and payed properly."(von Maur 2023 Alice Does not Care – Or: Wy it Matters That Robots "Don't Give a Damn", p. 226; in: Loh und Loh 2023 Social Robotics and the Good Life - The Normative Side of Forming Emotional Bonds With Robots)
  - Job loss following replacement through social robot → "From the caregiver's perspective, there is also the risk that they will lose their job due to the robot [5, 17, 18]." (Translation by the authors)(Radic and Vosen 2020 Ethische, rechtliche und soziale Anforderungen an Assistenzroboter in der Pflege – Sicht des Führungspersonals in Kliniken und Pflegeeinrichtungen, p. 630)
  - *Cont. on next page*
- *Cont. on next page*

Ethical hazards for care providers/facilities (including caring relatives) (continued)

- Aggravate work situation for care providers (continued)
  - Reduction of (the importance of) relationship work → "Nurses from a clinical setting explained they often work together with colleagues and are therefore worried about less teamwork when working with the robot: 'And there is togetherness and teamwork at the bedside when staff help each other to move patients or make the beds, and that would then all be lost' (PN 13, FG 1)." (Nielsen et al. 2022 Implementing ethical aspects in the development of a robotic system for nursing care: a qualitative approach, p. 5)
  - Conflict among care providers how to use the social robot → "At the same time, it is important to keep in mind the interests of the carers. Different carers may have different desires or interests regarding how to care for older people and may disagree about how care should be discharged, including the care roles that a robot should assume (Jenkins and Draper 2015). What is beneficial for one type of carer may be detrimental to another."(Tan et al. 2021 Tensions and antagonistic interactions of risks and ethics of using robotics and autonomous systems in long-term care, p. 9)
  - Loss of trust in care providers by care recipients → "Conversely, however, the presence of a robot to monitor the care delivered by a FC may, if this is known to the older person, undermine the faith that this older person then has in the carer."(Jenkins and Draper 2015 Care, Monitoring, and Companionship: Views on Care Robots from Older People and Their Carer, p. 678)
- Feeling insecure in handling social robots → "Reluctance to embrace robots may be due to a variety of reasons such as fear of being replaced by a robot, fear of change, fear of operating highly advanced technology, [. . . ] (Broadbent et al. 2010; Verrugio & Operto, 2008)." (Huschilt et al. 2012 The Use of Socially Assistive Robots for Dementia Care, p. 17)
  - Lack of competencies for handling social robots → "The staff felt unskilled and that they had no time to learn to use the robot."(Niemelä et al. 2021Towards Ethical Guidelines of Using Telepresence Robots in Residential Care, p. 433)
  - Need to re-negotiate own role in care → "The introduction of SARs challenges current roles and responsibilities in care practice settings, thus threatening the quality of the practice and the essential element of trust that is constitutive of the relationship between caregivers and care recipients. Which tasks can be responsibly delegated to SARs or not in order to legitimately reshape these roles is a question with deep ethical implications at the organizational level."(Boada et al. 2021 The ethical issues of social assistive robotics: A critical literature review, p. 9)
- Complicating management for care facilities → "Purchasing health robots for use at a facility can entail large financial costs, resulting in the discontinuation of other activities."(The Swedish National Council on Medical Ethics 2015 Robots and Surveillance in Health Care of the Elderly – Ethical Aspects, p. 447))
  - *Cont. on next page*
- *Cont. on next page*

Ethical hazards for care providers/facilities (including caring relatives) (continued)

- Complicating management for care facilities (continued)
  - Mismanagement due to complex changes/unclear consequence
    → "'We started using these systems in order to have a unique selling point. Back then, hardly anyone did that. But it wasn't economically viable at the time', says Adler. Her care service was one of the first in the region to use autonomous systems. 'We had too many false alarms. That's expensive.' Autonomous systems in care initially worked with fixed values. For example, the robot requested a blood pressure measurement and transmitted the data directly to the nursing service. 'But not everyone's blood pressure is the same', says Jana Fiedler." (Translation by the authors)(Seefeldt and Hülsken-Giesler 2020 Pflegeethik und Robotik in der Pflege, p. 272)
    - Possible loss of nursing staff skills due to social robots
      → "Moreover, integration of robots in the healthcare services may also contribute to a deskilling of the personnel [8], i.e., if some of the tasks are carried out by the robot instead of the human caregiver, the human caregiver will not get the necessary hands-on practice in dealing with those types of tasks."(Saplacan et al. 2021 On Ethical Challenges Raised by Care Robots: A Review of the Existing Regulatory-, Theoretical-, and Research Gaps, p. 223)
    - Investment might turn into waste of resource
      → "A Reuters article (21st September 2007) reported that director of a Japanese care home had said that residents 'liked the ifbot for about a month before they lost interest'. The robot had spent most of the past 2 years languishing alone in a corner, and the director of the facility commented, 'stuffed animals are more popular'." (Sharkey and Sharkey 2012 Granny and the robots: ethical issues in robot care for the elderly, pp. 34, 35)
  - Social robots turn out strategic disadvantage on the market
    → "Robots that support care are viewed very critically by senior citizens. Nursing homes with robots do not necessarily enhance the status of an inpatient facility [...]." (Translation by the authors) (Kapitel Meyer 2011 Akzeptanz ausgewählter Anwendungsszenarien, p. 103; in: Meyer 2011 Mein Freund der Roboter - Servicerobotik für ältere Menschen - eine Antwort auf den demographischen Wandel?)
    - Social robots perceived as indicator for low quality of care
      → "The importance of broadening the perspective in this way is shown by the assessment of care facilities in which robots are used. For the interviewees, this would not be a factor that enhances this facility, in contrast to modern furnishings, a cozy atmosphere or appealing lighting. They would assume that the facility does not have enough nursing staff." (Translation by the authors)(Kapitel Meyer 2011 Akzeptanz ausgewählter Anwendungsszenarien, p. 101; in: Meyer 2011 Mein Freund der Roboter - Servicerobotik für ältere Menschen - eine Antwort auf den demographischen Wandel?)
    - *Cont. on next page*
  - *Cont. on next page*
- *Cont. on next page*

Ethical hazards for care providers/facilities (including caring relatives) (continued)

- Complicating management for care facilities (continued)
  - Social robots turn out strategic disadvantage on the market (continued)
    - Negative publicity for care institutions when risks realize → "The data captured by robotics and autonomous systems deployed in long-term care settings cover a significant amount of an old person's life at the end of the developmental curve. This information is highly sensitive and intimate especially when it comes to data collected towards the end-of-life of the care recipients. If these data are breached for unsolicited purposes that are detrimental and malicious, it will result in strong distrust from the care recipients and their families, as well as negative publicities for the care institutions (Ienca et al., 2016)." (Tan et al. 2021 Tensions and antagonistic interactions of risks and ethics of using robotics and autonomous systems in long-term care, p. 8)
  - Problems with ongoing maintenance and support → "Currently, universal access by fair opportunity to assistive technologies is an ideal but not a reality. A few studies also highlighted ongoing maintenance, cleaning and repair can be an added cost [17, 36]." (Hung et al 2019 The benefits of and barriers to using a social robot PARO in a care setting: a scoping review, p. 6)
  - High training effort → "One researcher participant said, 'I have seen the hurdle of training. It is a lot of work to motivate people learn about what the robots do and how to use them. Some LTC homes just not have the capacity to adopt new practice and support staff training.'"(Hung et al. 2022 Technological risks and ethical implications of using robots in long-term care, p. 4)
  - Loss of skilled personnel due to replacement → → "By substituting robots for human health care professionals, we could lose many dynamic, highly skilled knowledgeable, and compassionate professionals - both regulated and unregulated - who are experts in the care for people with dementia (Butter et al., 2008; Veruggio & Operto, 2008)." (Huschilt and Clune 2012 The Use of Socially Assistive Robots for Dementia Care, p. 18)
- Lack of protection due to lack of guidelines/legal regulations → "Legal concerns exist about the use of SARs for dementia care: Robots are not people and members of a regulatory body. Currently, robots and the performance of robotic tasks are not regulated, nor do they require a registration or license. There is no legally defined scope of practice for robots. Therefore, SARs are not sanctioned to perform interventions that are authorized for licensed nurses. Questions from this premise emerge: What happens if a regulated health care professional delegates a task to the SAR? Who should be held accountable for a robot's error – the robot, the delegating professional, the designer, the programmer, the manufacturer, or the patient? (Feil-Seifer & Mataric, 2011; Veruggio & Operto, 2008). All of these issues must be considered if SARs are to enter mainstream dementia care approaches." (Huschilt et al. 2012 The Use of Socially Assistive Robots for Dementia care, p. 17)

Ethical opportunities for society/the (healthcare) system (Main category)

- Supporting state in ensuring comprehensive nursing care for all → “For the majority of older people and their families there is no reason why buying something that helps with care should not be a normal consumer purchase. While it has not yet been possible for telecare equipment to break into a mass consumer market, similar functionality but packaged in a small robot may have the potential to make it to supermarket shelves.” (Blackman 2013 Care robots for the supermarket shelf: a product gap in assistive technologies, p. 767)
  - Efficient care provision → “They also address the need to offer and provide acceptable care as economically as possible.”(Draper and Sorell 2017 Ethical values and social care robots for older people: an international qualitative study, p. 50)
    - Reducing costs of care overall → “Finally, last but not least, among the strongest motivations for introducing care robots is to reduce the cost of healthcare [. . . ].”(Salvini 2015 On Ethical, Legal and Social Issues of Care Robots, p. 432; in: Mohammed 2015 Intelligent Assistive Robots - Recent Advances in Assistive Robotics for Everyday Activities)
  - Furthering equal access to/provision of care → “SAR has implications for social equality, since depending on how it is developed and implemented, it may either contribute to increase or lessen the equality of care both in terms of access and quality of treatment.”(Boada et al. 2021 The ethical issues of social assistive robotics: A critical literature review, p. 9)
    - Social robots treat all patients equally → “Some participants maintained that robots could be more caring than humans and noted that ‘robot care’ would sometimes be preferential. The reasons given for such were that robots treat all patients equally.” (Nyholm 2021 Users ambivalent sense of security with humanoid robots in healthcare, p. 224)
    - Ensuring that care is available in rural areas → “In particular, technology-supported case and care management and telematics-supported systems in general (tele-nursing) are likely to gain in importance in future in view of the expected increase in the number of very old people living alone and also very old people remaining alone in (rural) regions, and will become an important field of activity for outpatient care services, especially in structurally weak regions.” (Translation by the authors)(Bleses and Dammert 2020 Neue Technologien aus Sicht der Pflegewissenschaft, p. 60; in: Hanika 2020 Künstliche Intelligenz, Robotik und autonome Systeme in der Gesundheitsversorgung)
    - *Cont. on next page*
- *Cont. on next page*

Ethical opportunities for society/the (healthcare) system (continued)

- Supporting state in ensuring comprehensive nursing care for all (continued)
  - Cushioning the nursing shortage/demographic change → "One of the areas of society in which such issues are currently becoming particularly virulent is care for the elderly, which is facing enormous challenges as a result of demographic change: The number of people in need of care has been rising for years, while it is becoming increasingly difficult to recruit nursing staff in sufficient numbers. A massive care gap is already emerging today, which is making the call for technical and, in particular, robotic support louder - innovative assistance technologies for care have been promoted by politicians for years [...]." (Translation by the authors)(Kehl 2018 Entgrenzungen Zwischen Mensch Und Maschine, Oder: Können Roboter Gute Pflege Leisten?, p. 3)
- Reducing discrimination/stigmatization of certain groups → "According to its proponents, this approach [approach of neutralization] could help to circumvent morally contested issues like the reproduction of gender stereotypes in care 'because you can probably only get into trouble with that [i.e. gender attributions]' (I16, 41). One marketing expert argued that a neutral approach was more inclusive and facilitated the establishment of a personal relationship: 'At the moment everything is gender neutral. Pepper addresses people mostly with 'you'. That certainly is a door opener because you do not address a person as 'mister' or 'miss' but directly create a personal connection' (I4, 27)."(Weßel et al. 2022 Gender and Age Stereotypes in Robotics for Eldercare: Ethical Implications of Stakeholder Perspectives from Technology Development, Industry, and Nursing, p. 8)
  - Decreasing the taboo around sexuality in old age/disability → "Our aim for doing this was to help recognize that though their common denominator is sex, disability is only partly a framing parameter. It is therefore a case where all these sex gadgets, sex toys and other sex innovations are actually bridging the gaps in thinking on this subject, rather than letting the gaps grow bigger, which is a rather positive development in our view."(Koumpis and Gees 2020 Sex with Robots: A not-so-niche market for disabled and older persons, p. 229)
- Promoting desired behavior of citizens → "As we have pointed out, the use of robots may actually even have the potential to alleviate or circumvent some of the pertinent pitfalls and problems, e.g., the sociopsychological mechanisms of stereotype threat that can impede the performance of male nurses in a traditionally female profession."(Weßel et al. 2021 Gender Stereotyping of Robotic Systems in Eldercare: An Exploratory Analysis of Ethical Problems and Possible Solutions, p. 11)
  - Developing moral skills of citizens → "On the one hand, because of their 'interpersonal' particular kind of interaction with humans, social robots may influence and shape human moral character, by potentially cultivating both vices and virtues."(Boada et al. 2021 The ethical issues of social assistive robotics: A critical literature review, p. 7)
  - *Cont. on next page*
- *Cont. on next page*

- Ethical opportunities for society/the (healthcare) system (continued)
  - Promoting desired behavior of citizens (continued)
    - Nudging users to desirable behavior via social robot design → “Beyond, there are also discussions as to whether social robots should be designed with ‘opt-in’, ‘opt-out’ or a ‘no way out’ design features to nudge users towards espousing personal interest or collective interests of the society (Borenstein et al., 2016).”(Tan et al. 2021 Tensions and antagonistic interactions of risks and ethics of using robotics and autonomous systems in long-term care, p. 12)
  - Important field for economic development (location advantage) → “Various focus technologies are commonly discussed under the heading of Care 4.0, from electronic documentation to AAL systems (Ambient Assisted Living), telecare and telepresence medicine through to robotics. The latter is at the center of this statement because it is considered to be particularly promising for the future in terms of overcoming growing care challenges, but also as a driver of economic development.22” (Translation by the authors)(Deutscher Ethikrat 2020 Robotik für gute Pflege (Stellungnahme), p. 14)

Ethical hazards for society/the (healthcare) system (Main Category)

- Moving forward without an adequate knowledge/ discussion base →

  > "The fact that such dystopias are nevertheless so prominent in ethical and public discourse indicates, on the one hand, that automation efforts in the care sector raise normative questions of a completely new dimension. On the other hand, it also highlights the central deficits of the corresponding debates. As these mainly revolve around speculative scenarios, there is hardly any differentiated discussion of the potential and limits of robotics in care." (Translation by the authors)(Kehl 2018 Entgrenzungen Zwischen Mensch Und Maschine, Oder: Können Roboter Gute Pflege Leisten?, p. 5)

- Using a misguided approach to regulation/design of social robots →

  > "In terms of the decisions regarding the use of robots in care for PwD, the decision-makers (state-funding bodies, owners of private care or nursing homes) are not those who initially bear the costs (those in care). They are, in effect, testing robotic care for older people in 'living labs' – care homes. By the time those decision-makers will need that sort of care, many of the problems that initially are presented will have disappeared." (O'Brolcháin 2017 Robots and people with dementia: Unintended consequences and moral hazard, p. 968)

  - Prioritizing system/economic advantages over care recipients needs →

    > "Against this backdrop, fears cannot be dismissed that an accelerated use of robots could lead to care work being increasingly subjected to a mechanistic understanding, i.e. narrowed down to purpose-related aspects and the sentient aspects being marginalized accordingly - especially if this can lead to economic savings. This applies all the more as a one-sided understanding of nursing care as a rational problem-solving activity is part of overarching and long-standing standardization and economization efforts [...] which are increasingly subjecting not only nursing care but the healthcare system as a whole to the logic of business management." (Translation by the authors)(Kehl 2018 Entgrenzungen Zwischen Mensch Und Maschine, Oder: Können Roboter Gute Pflege Leisten?, p. 6)

  - Working with underdeveloped ethical concepts →

    > "For instance, (human) freedom is currently understood as what is philosophically known as 'negative liberty' [79]. The ethical implications of SAR regarding this issue, though, could and should be broadened up by approaching the matter from a deeper understanding of this notion in its dimension of 'positive liberty' [79]. That is, closer to freedom as autonomy –in which freedom has to do with self-realization, with taking over the own life–. It could also be interesting to think from the perspective of Pettit's 'republican conception of freedom as non-domination' [84]. Delving into the meaning of this notion would definitely allow for a richer normative-oriented reflection on SAR that takes into account the interdependence existing between freedom and the sociopolitical structuring of human life, thus approaching SAR's power of domination both at an interpersonal and structural level." (Boada et al. 2021 The ethical issues of social assistive robotics: A critical literature review, p. 10)

  - *Cont. on next page*

- *Cont. on next page*

Ethical hazards for society/the (healthcare) system (continued)

- Using a misguided approach to regulation/design of social robots (continued)
  - Ignoring relevant aspects of the care situation → "Most of the socially assistive robots have so far been tested only in laboratory settings. Consequently, there is a scarcity of knowledge about their use in real care situations from the point of view of older care-dependent people, especially regarding their use by persons with dementia, and particularly considering the application in domestic environments. Concretely, the testing of robots in laboratory settings cannot capture the complexity and high variability of everyday real-life situations in nursing care (Bioethics Commission, 2018)."(Paletta et al. 2019 AMIGO–A Socially Assistive Robot for Coaching Multimodal Training of Persons with Dementia, p. 267; in: Korn 2019 Social Robots: Technological, Societal and Ethical Aspects of Human-Robot Interaction)
  - Premature regulation not yet knowing relevant implications → "The source of normative uncertainties and therefore the subject of ethical evaluation is generally not the technology itself, but its role in specific socio-technical contexts of action (Grunwald 2013a, p. 4). This essentially includes the intended and unintended consequences that are associated with the use of technology and whose moral implications need to be clarified ethically. In fields such as care robotics, which are still at a very early stage of development and whose potential applications are therefore still unclear, this consequentialist (consequence-oriented) approach is confronted with fundamental problems - after all, the consequences of technology are still largely in the dark, which means that their ethical assessment is also largely in the dark (which, however, is basically a problem of insufficient prognostic knowledge and not a problem of normative evaluation per se)." (Translation by the authors)(Kehl 2018 Wege zu verantwortungsvoller Forschung und Entwicklung im Bereich der Pfegerobotik: Die ambivalente Rolle der Ethik, p. 148; in: Bendel 2018 Pflegeroboter)
  - Unclear or lacking regulation providing limited orientation → "From a regulatory perspective, robots for dementia are often in a gray zone between the regulation of medical applications and that of general ICT applications. A striking example is Paro, who is classified as Class 2 medical device by the U.S. FDA regulation but not in the EU."(Ienca et al. 2016 Social and Assistive Robotics in Dementia Care: Ethical Recommendations for Research and Practice, p. 571)
  - Further problematic attitudes/approaches
    - Moral decisions are too complex for adequate social robot programming → "In my opinion, there is little to suggest that Bentham or Kant could return in the form of care robots in the near future. The technical difficulties of implementing a moral-philosophical approach in the form of algorithms are far too great." (Translation by the authors)(Hilgendorf 2018 Recht und Ethik in der Pflegerobotik – ein Überblick, p. 383)
    - *Cont. on next page*
  - *Cont. on next page*
- *Cont. on next page*

Ethical hazards for society/the (healthcare) system (continued)

Using a misguided approach to regulation/design of social robots (continued)

Further problematic attitudes/approaches (continued)

Guidelines without processes to hold people accountable →

"To indeed make roboticists 'accountable for the social, environmental and human health impacts that robotics may impose on the present and future generations,' as the EP mentions, we encourage central institutions to develop these codes and establish consequences for violations, concerning fines and sanctions. The law could make them binding. Another way to make them binding would be to include them into a private contract, between a robot producer and a user – in this case a healthcare institution [131]." (Fosch-Villaronga and Albo-Canals 2019 "I'll take care of you," said the robot, p. 89)

Approaches that are not ELSA-informed →

"Coding literacy may be necessary for STEM curricula and may provide students employability in the future [113]. Still, this may entail a greater disconnection from what constitutes to be human in the future. As Bauman highlights, the more we are in the virtual type of proximity, the less time we spend in learning and acquiring the skills needed in the non-virtual type of reality [107]. Not including ethics, philosophy, and history in school curricula may probably prevent future generations from not only solving ethical questions arisen from technology (problem-solving) but what it is more worrying, from identifying such problems and issues in the very first place (problem-finding)." (Fosch-Villaronga and Albo-Canals 2019 "I'll take care of you," said the robot, p. 85)

Focusing on physical instead of cognitive/emotional harm →

"While this standard is unique in addressing service robot related physical safety issues [53], robots used in therapeutic settings interact with the user mostly socially [54, 55]. Social robots express and perceive emotions, communicate in high-level dialogue, learn/recognize models of other agents, establish and maintain social relationships, use natural cues such as gaze or gestures, exhibit distinctive personality and character, and might learn or even develop social competencies [56]. Those social robots that assist users through social interaction have been called socially assistive robots (SAR) [57]. Socially-interactive robots raise the question of whether available service robot safety standards suffice to mitigate hazards that mostly relate to cognitive aspects. Since the European Parliament (EP) on its latest resolution concerning the legal aspects of robot technology mentions, 'human dignity and autonomy – both physical and psychological – is always to be respected,' in this subsection we address some points that challenge this cognitive side." (Fosch-Villaronga and Albo-Canals 2019 "I'll take care of you," said the robot, p. 82)

*Cont. on next page*

*Cont. on next page*

*Cont. on next page*

Ethical hazards for society/the (healthcare) system (continued)

- Using a misguided approach to regulation/design of social robots (continued)
  - Further problematic attitudes/approaches (continued)
    - Ignoring consequences of social robots in the long run → "There are few, if any, mechanisms that protect users from broader and long-term consequences of a particular technology. Indeed, designers may correctly specify an objective function, their system may perform a task in the environment, but they may ignore the fact that their technology may have broader consequences in the long run."(Fosch-Villaronga and Albo Canals 2019 "I'll take care of you," said the robot - Reflecting upon the legal and ethical aspects of the use and development of social robots for therapy, p. 84)
    - Focusing too much on implications for the individual → "The individual-centered perspective comes with a disproportionately fewer attention to SAR implications from the perspective of the (care) practice in which its artifacts are used, as well as from the macro sociopolitical level of justice. Descriptively, several reasons could be found behind this tendency.11 However, from a normative-oriented point of view, what matters the most is that this is an important deficiency of the current ethical approach to SAR, which shows a continuation of the individualist assumptions and the 'neglect of the political' underlying the mainstream philosophy of technology and ethics of technology [82]."(Boada et al. 2021 The ethical issues of social assistive robotics: A critical literature review, p. 10)
    - Seeing digital sexuality as pathological instead of atypical → "Those who favour the advent of sex with robots are often perceived as being sexually deviant, even perverted."(Koumpis and Gees 2020 Sex with Robots: A not-so-niche market for disabled and older persons, p. 228)
    - Understanding vulnerability solely as annoyance/capacity problem → "The idea that individual nursing activities could be performed by technology or robots harbors the risk of falling back into a deficit-oriented view of patients." (Translation by the authors)(Manzeschke and Petersen 2020 Digitalisierung und Roboterisierung in der Pflege – Ethisch-anthropologische Überlegungen, pp. 169, 170)
    - Raising false hopes without really assessing technical feasibility → "Visions of technical futures are painted and unfolded in a quasi-deterministic inevitability, the relevance of which appears at least questionable on closer inspection. Real challenges, which primarily relate to the shaping of technological development, are lost from view. Not least, this raises false expectations among the public and paints a picture of care robotics and its capabilities that does not correspond to reality." (Translation by the authors)(Kehl 2018 Wege zu verantwortungsvoller Forschung und Entwicklung im Bereich der Pfegerobotik: Die ambivalente Rolle der Ethik, p. 155; in: Bendel 2018 Pflegeroboter)
    - *Cont. on next page*
  - *Cont. on next page*
- *Cont. on next page*

Ethical hazards for society/the (healthcare) system (continued)

- Beneficial social robots not used to due diverse barriers → "Finally, in addition to the lack of functionality of the device and a lack of knowledge on the part of employees, structural barriers to the use of the device are also recognized. The key issues here are cost coverage and access to the internet. In addition to the lack of cost efficiency and doubts about effectiveness, this aspect is considered to be the reason for the generally still low spread of technical assistance systems (Weinberger & Decker, 2015)." (Translation by the authors)(Geier et al. 2020 Wie Pflegekräfte im ambulanten Bereich den Einsatz von Telepräsenzsystemen einschätzen – eine qualitative Studie, p. 49)
  - Lack of acceptance/fear of beneficial social robots → "They state that one major problem that impedes the implementation of robots in elderly care is the population's fear towards robots in care in general and older people's fear in particular (see also Nomura, Kanda and Suzuki 2006)."(Hoppe et al. 2020 Assistive robots in care: Expectations and perceptions of older people, p. 140; in: Haltaufderheide et al. 2020 Aging between Participation and Simulation - Ethical Dimensions of Social Assistive Technologies)
    - Inadequate design for target groups leads to skepticism → "As unmet users' expectations are a major indicator of suboptimal adoption, the lack of user-oriented approaches in product design risks to generate a vicious circle where unmet user needs cause lower-than-expected uptake which, in turn, perpetuates unmet user needs."(Ienca et al. 2016 Social and Assistive Robotics in Dementia Care: Ethical Recommendations for Research and Practice, p. 567)
    - Lack of knowledge about social robots leads to skepticism → "However, it has been found that public knowledge about robots seems vague, as the public tends to lack information on how care robots can be used in elder care [35]. Previous studies report that when participants did not have experiences with the robot in question, more negative attitudes were prevalent [56, 57]."(Johansson-Pajala et al. 2020 Care Robot Orientation: What, Who and How? Potential Users' Perceptions, p. 1105)
  - High costs prevent usage/development of social robots → "Despite many years of development efforts and countless prototypes, only a handful of systems have made it into practical use so far - all of them special applications (in addition to the Paro seal, these include some eating aids as well as a few exoskeletons and telepresence robots), which have no gripper arms and only limited autonomy and therefore deviate significantly from the model of the multifunctional everyday assistant. This is not only due to the high technical hurdles that need to be overcome. There is also a difficult market environment: as the care sector is under high economic pressure and only a few service providers have sufficient investment potential, it is unclear whether the high development costs can be amortized." (Translation by the authors) (Kehl 2018 Entgrenzungen Zwischen Mensch Und Maschine, Oder: Können Roboter Gute Pflege Leisten?, pp. 4, 5)
  - Further barriers → "The robotic device not being adapted to the intended environment (eg, it was too large, or movement pattern did not fit in the setting) and the technology failing in certain locations were seen as barriers as well." (Servaty et al. 2020 Implementation of robotic devices in nursing care. Barriers and facilitators: an integrative review, p. 9)
- *Cont. on next page*

Ethical hazards for society/the (healthcare) system (continued)

- Problematic change in (under)standing of care → "One problem that is currently arising for nursing as a profession, but which could possibly change our society as a whole with regard to our understanding of help and care, is the question of whether and how our human understanding of help and care will be reshaped if robots take over a not inconsiderable part of these activities and a 'feeling' for the interpersonal dimension of help is lost because the functional element is so strongly in the foreground." (Translation by the authors)(Manzeschke 2019 Roboter in der Pflege - Von Menschen, Maschinen und anderen hilfreichen Wesen, p. 5)
  - Reduction in significance of care → "[. . . ] the central ethical question is how to reshape the traditional roles and functions of the professionals in the practice, i.e., which are the tasks that should be delegated to robots and why. Human substitution by robots could not only impact the practice quality, but also the meaning of care."(Boada et al. 2021 The ethical issues of social assistive robotics: A critical literature review, p. 8)
  - Dehumanization/mechanization of care → "However, there are also fundamental objections that are generally directed against the use of robots in care and to which the systems presented here in more detail are also exposed. One particularly daunting idea is that robots could one day completely replace human interaction in care. The elderly would then live isolated from the rest of the world in care facilities where they are fed, washed and monitored by robots that are also responsible for entertainment and attention." (Translation by the authors)(Misselhorn 2018 Pflegesysteme, p. 152; in: Misselhorn 2018 Grundfragen der Maschinenethik)
    - Reductionist view of care as input-output process → "Reducing the complex process of caring into single sequences of input and output, does harm to this phenomenon and carries the risk to change it ultimately. The normalization of implementing care robots to fight loneliness bears the danger of a structural and even institutionalized (acceptance of) objectification and that we may not even expect essential features of care from humans anymore, if we get more and more used to encounters where our opposite does not care about us. This is to be phrased as a hypothetical state of affairs, as a potential outcome of the implementation and acceptance – the normalization – of care robots in societies."(von Maur 2023 Alice Does not Care – Or: Wy it Matters That Robots "Don't Give a Damn", p. 226; in: Loh und Loh 2023 Social Robotics and the Good Life - The Normative Side of Forming Emotional Bonds With Robots)
- Problematic change in understanding of being human → "It is argued that the robot degrades the person in need of help to a problem that can be solved by technology." (Translation by the authors)(Radic and Vosen 2020 Ethische, rechtliche und soziale Anforderungen an Assistenzroboter in der Pflege – Sicht des Führungspersonals in Kliniken und Pflegeeinrichtungen, p. 630)
  - Humanizing social robots and objectifying humans → "But if we – we, as the society being responsible for the narratives and imaginaries which make certain solutions to societal problems visible while obscuring others – do not see the robot as an object, but rather endorse the illusion that a robot cares about us, we humanize it and objectify ourselves: We objectify ourselves by humanizing an object.17"(Von Maur 2023 Alice Does not Care – Or: Wy it Matters That Robots "Don't Give a Damn", p. 225; in: Loh und Loh 2023 Social Robotics and the Good Life - The Normative Side of Forming Emotional Bonds With Robots)
- *Cont. on next page*

Ethical hazards for society/the (healthcare) system (continued)

- Damaging interpersonal and societal connectedness → "The use of social robotics in care for PwD will diminish the social relationship between healthy independent adults and PwD, by moving the latter further out of the mainstream of society and rendering them socially invisible "(O'Brolchain 2017 Robots and people with dementia: Unintended consequences and moral hazard, p. 969)
  - Normalization of robotic care changes expectation in care/interaction → "One structural effect of normalizing the implementation of care robots could be that humans will not only stay lonely while interacting with robots, but that they will give up expectations of real care and true relationships in the first place."(von Maur 2023 Alice Does not Care – Or: Wy it Matters That Robots "Don't Give a Damn", p. 210; in: Loh und Loh 2023 Social Robotics and the Good Life - The Normative Side of Forming Emotional Bonds With Robots)
    - Fostering an instrumentalist view of relationships → "Second, because the unauthentic intersubjectivity involved in HRI may affect the proper development or exercising of human moral faculties (empathy, care . . . ), as well as foster an instrumentalist view of relationships, in which the 'other' is not a 'you' but rather an object. This endangers relations and forms of life that are intrinsically valuable and define us."(Boada et al. 2021 The ethical issues of social assistive robotics: A critical literature review, p. 7)
  - No exchange/understanding between generations → "The implication to society as a whole could be dire in the long-term as this would mean that the society, especially the younger generation, will be less cognizant about the challenges one faces at old age, including having less exposure to the vulnerability and infirmity that may inflict the older generation. The changing nature of social relationships might also erode opportunities to extend virtues such as care and empathy to functionally dependant [sic] older people (O'Brolchain 2017)."(Tan et al. 2021 Tensions and antagonistic interactions of risks and ethics of using robotics and autonomous systems in long-term care, p. 10)
- Moral de-skilling → "SAR may disrupt human moral practices that are constitutive of our societies and culture and, in turn, endanger both the internal goods of these practices, and certain human moral capacities that can only be developed and exercised through these forms of activity. SAR may erode care as a central practice of human moral life because it may reduce our engagement in such activity, thus lessening the cultivation of its associated moral skills and leading to a moral and professional deskilling. This has implications for the organizational sphere of human life, given that it challenges the core values of the exercised practice of care, which calls into question those of the whole institutional context within which is held."(Boada et al. 2021 The ethical issues of social assistive robotics: A critical literature review, p. 8)
  - *Cont. on next page*
- *Cont. on next page*

Ethical hazards for society/the (healthcare) system (continued)

- Moral de-skilling (continued)
  - Reducing opportunities to develop moral skills → "On the other hand, the adoption of SARs in care, by outsourcing practices central to human existence to non-human actors, could blind us from the awareness of the constitutive vulnerability and (inter)dependance of human life, thus threatening the cultivation of virtues essential to a flourishing society. More specifically, the new technological practices could reduce the opportunities for cultivating moral skills regarding human caregiving."(Boada et al. 2021 The ethical issues of social assistive robotics: A critical literature review, p. 7)
    - By social robot never challenging personal biases and prejudices → "Eventually, the constant repetition of such everyday life effects of stereotyping in robotic systems for eldercare might even influence the users' personality itself by generating, promoting, or reinforcing biased perspectives and prejudiced attitudes. In the end, this could undermine their fundamental capability or willingness to critically reflect and overcome gender stereotypes and consequently cultivate a sexist and narrow-minded character." (Weßel et al. 2021 Gender Stereotyping of Robotic Systems in Elderly Care An Exploratory Analysis of Ethical Problems and Possible Solutions, p. 5)
    - Constrained communication/dialogue system of social robots reduces capacities for moral reasoning → "A partly adjacent concern related to human moral deskilling is the influence that language-capable robots, because of their acting as social moral agents (and given their constrained dialogue systems), may have on human moral reasoning."(Boada et al. 2021 The ethical issues of social assistive robotics: A critical literature review, p. 8)
  - Improperly extending treatment of social robot to humans → "Human improper behavior and interaction with robots could foster a human's moral corruption. Even without explicit or intentional abusive behavior, the very same simulation of unconditional recognition carried out by robots could lead to a moral deskilling (for instance, it could normalize the experience of exercising control and power over what is seen as an autonomous agent with recognitive abilities)."(Boada et al. 2021 The ethical issues of social assistive robotics: A critical literature review, p. 7)
    - Sex robots further improper treatment of women (or men) → "Professor Kathleen Richardson has published on this topic (see, for example, [14] and also her Campaign Against Sex Robots [13]) and offers several good reasons why sex robots are not a good idea at all. Among her arguments are the facts that 'sex robots further sexually objectify women and children'. One might of course think that sex robots similarly objectify men as well, but her argument is, of course, quite correct and valid."(Koumpis and Gees 2020 Sex with Robots: A not-so-niche market for disabled and older persons, p. 231)

- *Cont. on next page*

Ethical hazards for society/the (healthcare) system (continued)

- Fueling discrimination/stigmatization of certain groups → "These considerations already point to the fundamental concern that the very idea of stereotyping in and of itself contravenes basic egalitarian principles of justice in modern moral thought that call for equal respect and mutual recognition of all individuals. Thus, one could argue that stereotyping of care and social robots implies the affirmation and reinforcement of traditional gender stereotypes and therefore amounts to a technological promotion of existing societal bias, injustice, and discrimination."(Weßel et al. 2021 Gender Stereotyping of Robotic Systems in Elderly Care An Exploratory Analysis of Ethical Problems and Possible Solutions, p. 6)
  - Social robot programming reinforces (gender) participation gaps/inequality → "As far as the implementation of stereotyping strategies in the design or programming of eldercare robots is concerned, the question is how gender stereotypes affect the possibilities of participation and inclusion of older men and women in different areas of social life. Thus, it would appear rather objectionable if certain assistive functions were neglected or not promoted because they were connected to corresponding social practices that are considered as not gender-relevant or gender-inappropriate, e.g., needlework for older men or technical activities for older women. In particular, it would be highly problematic if stereotyping strategies in care robots for older people would simply adopt and reproduce norms that lead to a gender participation gap [56] and thus either systematically inhibit or impede the social and political participation of older women or consequently favor or even enforce the social or political participation of older men. Especially options for political participation should not be coupled to gender assignments but rather should be left to the individual citizen's own preferences and self-determined decisions."(Weßel et al. 2021 Gender Stereotyping of Robotic Systems in Elderly Care An Exploratory Analysis of Ethical Problems and Possible Solutions, p. 6)
  - Social robot design reinforces ageist stereotypes/deficit-orientation → "Neven analysed the interaction between robots, older test users and robot designers and found that the designers were influenced by stereotypes of older people as lonely, fragile, in need of care and company [53]."(Frennert and Östlund 2014 Review: Seven Matters of Concern of Social Robots and Older People, p. 303)
  - Social robot appearance reinforces racist/sexist/ethnic stereotypes → "Jecker notes that one criticism of using sex robots is that it may endorse an extreme form of objectification—sex robots are primarily designed to look like young attractive women, which may bolster society's propensity to objectify women for the purpose of serving heterosexual men." (Bianchi 2021 Considering sex robots for older adults with cognitive impairments, p. 37)
- Unfair distribution of benefits and burdens of social robot introduction → "Indeed, one's economic, social, physical, psychological, cultural-religious, educational, etc. capabilities and functionings should not lead to unfair advantages or disadvantages regarding the possibilities of SAR use (d'Avack et al., 2017; Kornfield-Matte, 2017; Palmerini et al., 2014)." (Vandemeulebroucke et al. 2021 Socially Assistive Robots in Aged Care: Ethical Orientations Beyond the Care-Romantic and Technology-Deterministic Gaze, p. 17)
  - *Cont. on next page*
- *Cont. on next page*

Ethical hazards for society/the (healthcare) system (continued)

- Unfair distribution of benefits and burdens of social robot introduction (continued)
  - Burdens are not shouldered by those reaping the benefits → "The question of whether only the affluent older people will be able to reap the benefits of robotics and autonomous systems and whether the less privileged older people will be disproportionately subjected to the unintended consequences of robotics and autonomous systems has been raised (Laitinen et al., 2016)."(Tan et al. 2021 Tensions and antagonistic interactions of risks and ethics of using robotics and autonomous systems in long-term care, p. 10)
  - Only rich can afford expensive human care (robot care is cheaper) → "Risk potentials correspond primarily with the fear that 'people in need of care will receive less human attention through the use of care robots' (y=0.48***), and secondly with the expectation that 'the use of robots will ultimately lead to only the wealthy being cared for by humans' (y=0.28***)." (Translation by the authors)(Zwick and Hampel 2019 Cui bono? Zum Für und Wider von Robotik in der Pflege – Ergebnisse einer Repräsentativbefragung, p. 54)
  - Unequal access to effective robot technology → "It is found that one of the noticeable issues in robot ethics is having equal access to assistive medical robots." (Mansouri et al. 2017 Ethical framework of assistive devices: review and reflection, p. 6)
    - Due to lack of customization options → "[...] the proportion of devices selectively designed to support specific stages of dementia is currently low. This lack of specificity could represent a significant obstacle toward the massive adoption of IATs for dementia and could add an additional reason to the limited uptake of IATs. In fact, patients suffering from different stages of AD may present specific needs and limitations that may be qualitatively and/or quantitatively different than those of people with other age-related cognitive disturbances or disabilities [50] . Also, people with mild AD often present different needs and limitations than people with moderate or advanced dementia and vice versa [51]." (Ienca 2016 Intelligent Assistive Technology for Alzheimer's Disease and Other Dementias: A Systematic Review, p. 1336)
    - Due to high costs and limited public finances → "Distributive justice is complicated by the fact that the more complex the assistive technology, the higher the risk that financial factors limit access for many individuals suffering from dementia and other disabilities (Alzheimer Europe 2010b). In view of these financial aspects, it will be necessary to avoid a digital divide and to find ways so that existing socioeconomic inequalities will not be increased."(Hildt 2019 Shaping the Development and Use of Intelligent Assistive Technologies in Dementia – Some Thoughts, pp. 133, 134; in: Jotterand et al. 2019 Intelligent Assistive Technologies for Dementia: Clinical, Ethical, Social and Regulatory Implications)
    - *Cont. on next page*
  - *Cont. on next page*
- *Cont. on next page*

Ethical hazards for society/the (healthcare) system (continued)

- Unfair distribution of benefits and burdens of social robot introduction (continued)
  - Unequal access to effective robot technology (continued)
    - Due to limited information among certain groups → "Clinicians and other health professionals are often unaware of new IATs and their applicability to dementia care, as little cooperation has occurred between technology development and medical implementation." (Ienca 2016 Intelligent Assistive Technology for Alzheimer's Disease and Other Dementias: A Systematic Review, p. 1302)
    - Social robots meet only needs of those groups participating in design → "This becomes even more important as many robots tested or actually used in the care sector were not originally and specifically designed for care purposes but, for example, as industrial robots like Kuka's LBR robotic arms, or as companion robots in general like SoftBank's Pepper."(Weßel et al. 2021 Gender Stereotyping of Robotic Systems in Elderly Care An Exploratory Analysis of Ethical Problems and Possible Solutions, p. 3)
    - Social robots meet only needs subset of population trained on → "According to the informants, it is vital that the potential users of the technology, such as older persons, families, and care professionals, participate in the development process, but this seem to be lacking for several reasons. Manufacturers and companies develop products without consulting the users, or they involve the users too late in the process, only when it is time to test the product." (Johansson-Pajala and Gustafsson 2022 Significant challenges when introducing care roots in Swedish elder care, p. 171)
    - Sex robots not produced for elderly due to ageism → "Jecker is right to call out sexual ageism. Older people often do have sexual needs, and this should not be stigmatised or ignored. But we worry that a focus on sex robots may inadvertently strengthen the very ageism that Jecker decries." (Earp and Grunt-Mejer 2021 Robots and sexual ethics, p. 2)
- Erosion of responsibility/gaps in the responsibility structure → "The erosion of responsibility in the development, approval and use of robotic technologies should be counteracted by establishing transparent responsibility structures." (Translation by the authors)(Deutscher Ethikrat 2020 Robotik für gute Pflege (Stellungnahme), p. 50)
  - *Cont. on next page*
- *Cont. on next page*

Ethical hazards for society/the (healthcare) system (continued)

- Erosion of responsibility/gaps in the responsibility structure (continued)
  - Underdefined liability where social robots make mistakes → As to current issues concerning liability, scholars point out that in case of damages caused by an autonomous robot it might be more difficult to identify a responsible due to the increased complexity of the causal chain 130]. As a matter of fact, to turn a robot into a market product involves many actors: manufacturer, importers, wholesalers, retailers, repairers, installers, inspectors, and the users. Moreover, as pointed out by Lehman-Wilzig, the manufacturer has now doubled into the hardware and the software manufacturers. Therefore, it could be more difficult to attribute or share the responsibility for a defect or a fault and in many cases there might be 'no one at fault!' [30]. The issue of the indeterminacy of liability is related to robot autonomy, but also to share control robot [. . . ]."(Salvini 2015 On Ethical, Legal and Social Issues of Care Robots, p. 441; in: Mohammed 2015 Intelligent Assistive Robots - Recent Advances in Assistive Robotics for Everyday Activities)
- Inefficient use of social robots → "Although IATs open up promising prospects for the future of dementia care, their adoption is still lower than expected [8]. This has been attributed to suboptimal information transfer and dissemination across technology development und medical implementation [9] as well as to the lack of solid and highly generalizable clinical validation of many IATs [10]." (Ienca 2016 Intelligent Assistive Technology for Alzheimer's Disease and Other Dementias: A Systematic Review, p. 1302)
  - Difficulty in continuing the use of social robot after projects end → "Implementation of technology was perceived as a time-consuming and complex process. One concern was the 'project problem', meaning that, when a project ends, it all ends, rather than the project being firmly anchored in the organisation, so that the technology can be implemented after the project ends. Another challenge is how to launch the products on the market. Often, for a single product and small businesses, there is a shortage of capital, financing, and investment, and the unattainable market even seems to have reduced the inflow of innovation."(Johansson-Pajala and Gustafsson 2022 Significant challenges when introducing care robots in Swedish elder care, p. 171)
  - Positive benefits of social robot use cannot sustain long-term → "However, reported positive effects could only be shown for a short time [20] and undesirable effects as well as obstacles in the application of SAR are supposed."(Lehmann et al. 2021 Using a Socially Assistive Robot in a Nursing Home, p. 149)
    - *Cont. on next page*
  - *Cont. on next page*
- *Cont. on next page*

Ethical hazards for society/the (healthcare) system (continued)

- Inefficient use of social robots (continued)
  - High costs of social robots and their implementation → "One major factor likely to influence the actual take-up of robots in elder care is cost. The high costs of current robots are likely to limit their adoption." (Sharkey and Sharkey 2012 The eldercare factory, p. 283)
    - Difficult to develop robots in a divided/small market → "This caring matrix points out four different target-groups for effective digital assisting tools in, as each sector of the matrix has other claims for assistance. This is one reason why it is difficult to develop effective 'CareSAR'-tools, as the market is divided in small sectors." (Hasenauer et al. 2022 Living Labs in Social Service Institutions: An Effective Method to Improve the Ethical, Reliable Use of Digital Assistive Robots to Support Social Services, p. 2)
- Ecologically and socially not sustainable — "Seemingly, it is the local distribution of benefits and costs that is under ethical reflection (with the exception of the question of ecological sustainability, which is presented as both a local and global matter of concern)." (Boada et al. 2021 The ethical issues of social assistive robotics: A critical literature review, p. 9)
  - Using of minerals that fuel conflict → "SAR use in aged-care settings forces societal stakeholders to tackle the problem of what has been called 'conflict minerals'. The sources of production materials and minerals to produce SARs need to be questioned." (Vandemeulebroucke et al. 2021 Socially Assistive Robots in Aged Care: Ethical Orientations Beyond the Care-Romantic and Technology-Deterministic Gaze, p. 17)
  - Working conditions in production not safe/healthy → "SAR use in aged-care settings forces societal stakeholders to tackle the problem of what has been called 'conflict minerals'. The sources of production materials and minerals to produce SARs need to be questioned. Efforts need to be made to improve working and social conditions for workers that mine, collect, and process minerals and materials in unhealthy and unsafe circumstances." (Vandemeulebroucke et al. 2021 Socially Assistive Robots in Aged Care: Ethical Orientations Beyond the Care-Romantic and Technology-Deterministic Gaze, p. 17)
  - Social robot production/usage/-dumping too resource intensive → "However, the supply of raw materials for robots, the energy consumption they require, and the dumping waste that these new care technologies generate are important ethical challenges of SAR." (Boada et al. 2021 The ethical issues of social assistive robotics: A critical literature review, p. 9)
- Social robots get too much power and endanger humans/human society → "In this mode of rule, which proceeds by transforming its commands into programs and its agents into automatons (or in the case of the care robots: which delegates its socio-political tasks, made necessary by demographic change, to automatons), power, which was already far away in the past, now becomes completely intangible." (Translation by the authors)(Andreas 2018 Autonomous Lethality. Lebenskritische Entscheidungen in der Roboterethik, p. 151; in: Andreas et al. 2018 Unterwachen und Schlafen. Anthrophile Medien nach dem Interface)

Unsettled questions (Main category)

- Do the benefits of social robots outweigh the costs? → "The principle of justice governs the fair distribution of scarce resources. This can be a very difficult topic when discussing experimental treatments such as SAR. The authors know of no SAR systems that are currently used outside the research setting, so discussion of the actual cases in the field is premature. However, we can presume that for the foreseeable future, robots will be somewhat expensive. Thus, a question that should be asked is: Do the benefits of SAR outweigh the costs? Like other proposed therapies, quality of life surveys or other methods for assessing medical economy can be used to assess relative benefits, and costs can be weighed against improvements observed [1], [24]. There does not seem to be a significant difference between calculating the costs and benefits of robots compared to other assistive devices." (Feil-Seifer and Mataric 2011 Socially Assistive Robotics - Ethical Issues Related to Technology, p. 30)

- Who should receive/how to allocate scarce (robotic) resources? → "Carebots are likely to be a relatively expensive resource, and decisions will have to be made to who gets what and why."(Gallagher et al. 2016 Robots in elder care: Some ethical questions, p. 370)

- Whose interests regarding social robot usage should be prioritized? → "One ethical question is only mentioned here in passing, but it is anything but trivial to answer: Should a robot be unconditionally aligned with the interests or preferences of the person in need of support? And if not, with what other interests and according to what criteria?" (Translation by the authors)(Manzeschke 2019 Roboter in der Pflege - Von Menschen, Maschinen und anderen hilfreichen Wesen, p. 7)

  - Should care benefit be realized if staff is negatively affected? → "One could argue that the robot should primarily help to care for the cared-for persons so that their wishes should have priority. However, these wishes might conflict with the aim of the care robot so that the care task cannot be executed as intended. The respective wishes might also be discriminating against other members of the care setting and thus collide with the principle of justice." (Weßel et al. 2021 Gender Stereotyping of Robotic Systems in Elderly Care An Exploratory Analysis of Ethical Problems and Possible Solutions, p. 10.)

  - To whom among care home residents should social robots be customized? → "With regard to autonomy, queering only seems to offer a solution where the robot is merely used by one person, or all users have common preferences about the design. Otherwise, conflicts can arise from the individualization of the design. This raises the question of whose preferences should be prioritized."(Weßel et al. 2021 Gender Stereotyping of Robotic Systems in Elderly Care An Exploratory Analysis of Ethical Problems and Possible Solutions, p. 10.)

- *Cont. on next page*

Unsettled questions (continued)

Design by stereotype to foster wellbeing/compliance while reproducing harmful effects/reducing acceptance of diverse ways of life?

→ "[. . . ] we then map possible moral problems and conflicts such as the tension between a potentially beneficial impact of stereotyping on user compliance and wellbeing on the one hand, and its implications for modern pluralistic values such as diversity and mutual recognition on the other." (Weßel et al. 2021 Gender Stereotyping of Robotic Systems in Eldercare: An Exploratory Analysis of Ethical Problems and Possible Solutions, p. 2)

What privacy risks are acceptable for security gains?

→ "It would therefore be much more interesting to find out how to deal with the various ambivalences that can arise when using autonomous assistance systems - important questions that arise particularly with dementia patients concern, for example, the possible deceptive nature of social therapy robots or the conflict between the protection of privacy on the one hand and safety-related welfare requirements on the other (for example in the course of technical monitoring of cognitively impaired people) (Remmers 2016)." (Translation by the authors)(Kehl 2018 Wege zu verantwortungsvoller Forschung und Entwicklung im Bereich der Pfegerobotik: Die ambivalente Rolle der Ethik, p. 149; in: Bendel 2018 Pflegeroboter)

Is deception about relationships acceptable to realize benefits?

→ "In fact, it cannot be ruled out that people with dementia in particular, who suffer from an advanced loss of mental capacity, are deceived about the nature of robots. In principle - as Nozick's thought experiment shows - it can also be assumed that people would prefer their existing reality to a perfect fictional world. However, stepping out of everyday reality for a short time in order to be fooled can be appealing. We often enjoy the variety of fictional parallel worlds with virtual reality glasses or in 4D cinemas and then return to our normal lives. Similarly, interacting with a robot does not mean permanently leaving the real world in favor of a preferred illusory existence. Taking into account the strong intuition that most people do not want to remain in a fictitious, albeit perfect, world, the exchange between humans and machines must also be limited to regulated and frequented time windows. Under these conditions, robots are very well able to maintain the interest of the person in need of care and improve the quality of life of those affected, contrary to what critics such as Sparrow/Sparrow say." (Translation by the authors)(Kreis 2018 Umsorgen, überwachen, unterhalten - sind Pflegeroboter ethisch vertretbar?, p. 221; in: Bendel 2018 Pflegeroboter)

*Cont. on next page*

Unsettled questions (continued)

- Is deception about relationships acceptable to realize benefits? (continued)
  - Do the benefits of anthropomorphization outweigh negative effects? → "Yet, the anthropomorphization of the robot does not necessarily always have to create harm but might also increase wellbeing. In fact, this possibility is explicitly used in technology development to support a robot's performance in its setting. For example, Matilda is designed as child-like in order not to appear threatening to older people with cognitive impairments [62]. In consequence, explaining that such a robot is only a machine could have detrimental effects on its utilization and functioning and the wellbeing of the users. Furthermore, it is possible that users have already formed an emotional attachment to a robot before it is explained to them, or an emotional attachment is crucial for the users to feel comfortable and to interact with the robot in an effective way. If the robot is 'de-humanized' by an explanation, this might ultimately affect these users' wellbeing because they do not want to use 'just' a machine but need an emotional bond to increase their wellbeing." (Weßel et al. 2021 Gender Stereotyping of Robotic Systems in Eldercare: An Exploratory Analysis of Ethical Problems and Possible Solutions, p. 8.)
- Should control of/access to social robots be restricted to reduce risk of harm? → "We also suggest that the use of the robot should reflect the autonomy of patients where possible. Our participants, however, were open to both formal and ICs limiting autonomy to ensure the safety of older people, especially where protecting safety might secure autonomy in the longer-term." (Jenkins and Draper 2015 Care, Monitoring, and Companionship: Views on Care Robots from Older People and Their Carer, p. 682)
  - Should customization be possible risking abuse by other actors? → "What if several people can instruct the robot, say, me, my children living remotely and my doctor. Who can instruct the robot to do what actions? If the instructions conflict, who's instructions take precedence?"(Bennett 2014 Robot Identity Assurance, p. 11)
- Who should/how to decide when people are cognitively impaired? → "Let us suppose that a person, throughout her entire life-time, has established a strong intellectual self-identity. She has seen people in nursing homes play with Paro and concluded that 'I am not the kind of person who is ever going to play with such a thing!' She informs her family about this decision and puts it – together with other things – down in her advance healthcare directive. She then develops late-stage dementia and moves into a residence. One day she sees a group of people interact with Paro and desperately wants to join in. Is it right to involve her in the group, or should we prevent her from playing with Paro?"(Misselhorn et al. 2013 Ethical Considerations Regarding the Use of Social Robots in the Fourth Age, pp. 130, 131)
- *Cont. on next page*

Unsettled questions (continued)

- Who bears responsibility for the social robot and its actions? → "Another justice-related issue when discussing robotics in socially assistive settings is the notion of responsibility: Who is responsible when things go wrong?" (Feil-Seifer und Mataric 2011 Socially Assistive Robotics - Ethical Issues Related to Technology, p. 30)

- (When) should social robots be allowed to act without consent to protect? → "The actions of robots can be advisable concerning consequentialism or utilitarian approaches. For instance, if an older person wants to walk on the high-traffic road and a robot can stop him, then what should the robot do? Herein, the problem would be based on the robot's classification algorithm about the dangerous activity. In another scenario, Should the robot stop a person from eating much food? Would it be fine to restrict an individual's liberty?" (Noori et al. 2019 Robot-Care for the Older People: Ethically Justified or Not?, p. 45)
  - Should social robots motivate/remind while possibly perceived as controlling? → "The use of care assistant robots also raises questions. How pushy may a robot become, for example, in reminding someone to take medication?" (Van Est et al. 2016 Robotisation as Rationalisation - In Search for a Human Robot Future, p. 45)

- Should autonomy be bestowed onto social robots (at all/to what extent)? → "In general, the use of social robots in therapy and care raises a number of ethical questions: How autonomous should a therapy robot be? How much control should the caregivers and therapists have?" (Translation by the authors)(Lange and Bauer 2021 - Eine Robbe für Oma - Die zukünftige Dauerausstellung Robotik im Deutschen Museum, S. 236; in: Inthorn und Seising 2021 Digitale Patientenversorgung - Zur Computerisierung von Diagnostik, Therapie und Pflege)
  - Should social robots have moral capabilities (and which ones)? → "Should a robot assistant have a moral reasoning system that tells it what is 'good care'?" (Kemenade et al. 2019 Do You Care for Robots That Care? Exploring the Opinions of Vocational Care Students on the Use of Healthcare Robots, p. 8)

- What normative status should we assign social robots? → "Robots, as they are currently being developed for care, are complex technical counterparts that enter into social interaction with humans, although it is not yet clear what social and consequently what normative status we should assign to these manifestations." (Translation by the authors) (Manzeschke 2019 Roboter in der Pflege - Von Menschen, Maschinen und anderen hilfreichen Wesen, p. 1)

- *Cont. on next page*

Unsettled questions (continued)

How do we understand the concept of care?

"Lifting patients out of their beds can be described as both goal-oriented and practice-oriented. From the goal-oriented perspective, it is simply a matter of changing the patient's position. In the practice-oriented sense, however, lifting is also about touch and empathic interaction between the caregiver and the patient. Depending on the perspective from which an action is viewed, the use of a care robot or assistance system in this case may or may not appear morally problematic: if the aim is simply to achieve the goal, it is fine to use the support of a machine. If the focus is on touch and empathic interaction, then this is questionable. There is no right or wrong perspective, but it depends largely on what meaning an action has for the person in need of care." (Translation by the authors)(Misselhorn 2018 Pflegesysteme, p. 154; in: Misselhorn 2018 Grundfragen der Maschinenethik)

Do social robots per se fail to care?

"For him, deep care means something like deep human emotions when looking at the person in need of care. This criterion is somewhat unclear, but according to my explanation it could be understood as follows: in the sense of deep care, the caregiver not only cares for the patient, but is concerned about the patient. He is existentially involved and offers this existential relationship to the patient. Robots are only suitable for superficial care. Of course, human nurses do not always provide deep care either. They sometimes carry out work like robots. But patients perceive them as human beings and they are always the objects of a human relationship for patients." (Translation by the authors)(Kovács 2021 Anthropologische und ethische Aspekte des Einsatzes von Robotern im Gesundheitssektor, p. 32; in: Inthorn und Seising 2021 Digitale Patientenversorgung – Zur Computerisierung von Diagnostik, Therapie und Pflege)
