# Supplementary material for: Ethical aspects of the use of social robots in caring for older people – a systematic qualitative review
Source: Med Health Care Philos. 2026 Feb 5;29(1):209–24. doi: 10.1007/s11019-025-10313-3 (PMC12960314; doi:10.1007/s11019-025-10313-3)
Supplement: Supplementary file 2 — Online Resource 2 (PDF 171 kb) [file 11019_2025_10313_MOESM2_ESM.pdf]

Online Resource 2 to: Ethical Aspects of the Use of Social Robots in Elderly Care

A Systematic Qualitative Review

Marianne Leineweber[1], Clara Victoria Keusgen[1], Marc Bubeck[1], Robert Ranisch[1*], Joschka Haltaufderheide[1†], Corinna Klingler[1†]

[1]Juniorprofessorship for Medical Ethics with a focus on Digitization, Faculty for Health Sciences Brandenburg, University of Potsdam, Am Mühlenberg 9, Potsdam, 14476, Brandenburg, Germany.

*Corresponding author(s). E-mail(s): ranisch@uni-potsdam.de;
†Joschka Haltaufderheide and Corinna Klingler contributed equally as last authors.

References of included fulltexts

[1] Akalin N, Kristoffersson A, Loutfi A (2019) Evaluating the Sense of Safety and Security in Human–Robot Interaction with Older People. In: Korn O (ed) Social Robots: Technological, Societal and Ethical Aspects of Human-Robot Interaction. Human-Computer Interaction Series, Springer, Cham, p 237–264

[2] Aldinhas Ferreira MI, Sequeira JS (2017) Robots in Ageing Societies. In: Aldinhas Ferreira MI, Silva Sequeira J, Tokhi MO, et al (eds) A World with Robots: International Conference on Robot Ethics: ICRE 2015, vol 84. Springer International Publishing, Cham, p 217–223, doi: 10.1007/978-3-319-46667-5_17

[3] Amirabdollahian F, den Akker Ro, Bedaf S, et al (2013) Assistive technology design and development for acceptable robotics companions for ageing years. Paladyn, Journal of Behavioral Robotics 4(2). doi: 10.2478/pjbr-2013-0007

[4] Amirabdollahian F, op den Akker R, Bedaf S, et al (2013) Accompany: Acceptable robotiCs COMPanions for AgeiNG Years — Multidimensional aspects of human-system interactions. In: 2013 6th International Conference on Human System Interactions (HSI). IEEE, pp 570–577, doi: 10.1109/HSI.2013.6577882

[5] Ammicht Quinn R (2019) Zwischen Fürsorge und Kontrolle. EthikJournal 5:1–20

[6] Anderson M (2020) Ein wertegesteuerter Roboter. Spektrum der Wissenschaft 20 Spezial Biologie Medizin Hirnforschung pp 3–5

[7] Anderson SL, Anderson M (2018) Ethische Roboter für die Altenpflege. In: 3TH1CS. Bundeszentrale für Politische Bildung, 2018, Bonn

[8] Andreas M (2018) Autonomous Lethality. Lebenskritische Entscheidungen in der Roboterethik. meson press, doi: 10.25969/MEDIAREP/1278

[9] Baisch S, Kolling T, Rühl S, et al (2018) Emotionale Roboter im Pflegekontext. Zeitschrift für Gerontologie und Geriatrie 51(1):16–24. doi: 10.1007/s00391-017-1346-8

[10] Battistuzzi L, Papadopoulos C, Hill T, et al (2021) Socially Assistive Robots, Older Adults and Research Ethics: The Case for Case-Based Ethics Training. International Journal of Social Robotics 13(4):647–659. doi: 10.1007/s12369-020-00652-x

[11] Battistuzzi L, Sgorbissa A, Papadopoulos C, et al (2018) Embedding Ethics in the Design of Culturally Competent Socially Assistive Robots. In: 2018 IEEE/RSJ International Conference on Intelligent Robots and Systems (IROS). IEEE, pp 1996–2001, doi: 10.1109/IROS.2018.8594361

[12] Bauberger S (2020) Welche KI? Carl Hanser Verlag GmbH & Co. KG, München, doi: 10.3139/9783446465527

[13] Bedaf S, Marti P, de Witte L (2019) What are the preferred characteristics of a service robot for the elderly? A multi-country focus group study with older adults and caregivers. Assistive Technology 31(3):147–157. doi: 10.1080/10400435.2017.1402390

[14] Bendel O (2018) Roboter im Gesundheitsbereich: Operations-, Therapie-und Pflegeroboter aus ethischer Sicht. In: Bendel O (ed) Pflegeroboter. Springer Fachmedien Wiesbaden, Wiesbaden, p 195–212

[15] Bendel O (2020) Care Robots with Sexual Assistance Functions. In: AAAI 2020 Spring Symposium "Applied AI in Healthcare: Safety, Community, and the Environment"(Stanford University), vol abs/2004.04428. CoRR, doi: 10.48550/arXiv.2004.04428

[16] Bennett B (2019) Technology, ageing and human rights: Challenges for an ageing world. International Journal of Law and Psychiatry 66:101449. doi: 10.1016/j.ijlp.2019.101449

[17] Bennett B, McDonald F, Beattie E, et al (2017) Assistive technologies for people with dementia: ethical considerations. Bulletin of the World Health Organization 95(11):749–755. doi: 10.2471/BLT.16.187484

[18] Bennett L (2014) Robot Identity Assurance. ITNOW 56(3):10–11. doi: 10.1093/itnow/bwu064

[19] Bertolini A, Arian S (2020) Do Robots Care?: Towards an Anthropocentric Framework in the Caring of Frail Individuals through Assistive Technology. In: Haltaufderheide J, Hovemann J, Vollmann J (eds) Aging between Participation and Simulation: Ethical Dimensions of Socially Assistive Technologies in Elderly Care. De Gruyter, p 35–52, doi: 10.1515/9783110677485-003

[20] Bianchi A (2021) Considering sex robots for older adults with cognitive impairments. Journal of Medical Ethics 47(1):37–38. doi: 10.1136/medethics-2020-106927

[21] Bioethikkommission beim Bundeskanzleramt Österreich (2019) Roboter in der Betreuung alter Menschen: Stellungnahme der Bioethikkommission. Jahrbuch für Wissenschaft und Ethik 24(1):355–384. doi: 10.1515/jwiet-2019-0015

[22] Blackman T (2013) Care robots for the supermarket shelf: a product gap in assistive technologies. Ageing and Society 33(5):763–781. doi: 10.1017/S0144686X1200027X

[23] Bleses H, Dammert M (2020) Neue Technologien aus Sicht der Pflegewissenschaft. In: Hanika H (ed) Künstliche Intelligenz, Robotik und autonome Systeme in der Gesundheitsversorgung. Verlag Wissenschaft & Praxis, Sternenfels, p 55–84

[24] Bleuler T, Caroni P (2021) Roboter in der Pflege: Welche Aufgaben können Roboter heute schon übernehmen? In: Bendel O (ed) Soziale Roboter: Technikwissenschaftliche, wirtschaftswissenschaftliche, philosophische, psychologische und soziologische Grundlagen. Springer Gabler, Wiesbaden, p 441–457, doi: 10.1007/978-3-658-31114-8

[25] Boada JP, Maestre BR, Genís CT (2021) The Ethical Issues of Social Assistive Robotics: A Critical Literature Review. Technology in Society 67:1–13. doi: 10.1016/j.techsoc.2021.101726

[26] Bogue R (2013) Robots to aid the disabled and the elderly. Industrial Robot: An International Journal 40(6):519–524. doi: 10.1108/IR-07-2013-372

[27] Boni-Saenz AA (2021) Are sex robots enough? Journal of Medical Ethics 47(1):35. doi: 10.1136/medethics-2020-106928

[28] Borenstein J, Pearson Y (2014) Robot Caregivers: Ethical Issues across the Human Lifespan. In: Lin P, Abney K, Bekey G (eds) Robot Ethics: The Ethical and Social Implications of Robotics. MIT Press, Cambridge, p 251–265

[29] Bradwell HL, Winnington R, Thill S, et al (2020) Ethical perceptions towards real-world use of companion robots with older people and people with dementia: survey opinions among younger adults. BMC Geriatrics 20(1). doi: 10.1186/s12877-020-01641-5

[30] Büro für Technikfolgen-Abschätzung beim Deutschen Bundestag (2018) Robotik in Der Pflege - Gesellschaftliche Herausforderungen. Berlin

[31] Byers P, Matthews S, Kennett J (2021) Truthfulness in Dementia Care. Bioethics 35(9):839–841. doi: 10.1111/bioe.12970

[32] Carnevale A (2017) "I Tech Care": How Healthcare Robotics Can Change the Future of Love, Solidarity, and Responsibility. In: Hakli R, Seibt J (eds) Sociality and Normativity for Robots. Studies in the Philosophy of Sociality, Springer International Publishing, Cham, p 217–232, doi: 10.1007/978-3-319-53133-5_11

[33] Carros F, Eilers H, Langendorf J, et al (2022) Roboter als intelligente Assistenten in Betreuung und Pflege - Grenzen und Perspektiven im Praxiseinsatz. In: Künstliche Intelligenz im Gesundheitswesen. Springer Gabler, 2022, Wiesbaden

[34] Casey D, Felzmann H, Pegman G, et al (2016) What People with Dementia Want: Designing MARIO an Acceptable Robot Companion. In: Miesenberger K, Bühler C, Penaz P (eds) Computers Helping People with Special Needs, Lecture Notes in Computer Science, vol 9758. Springer International Publishing, Cham, p 318–325, doi: 10.1007/978-3-319-41264-1_44

[35] Coeckelbergh M (2015) Care Robots and the Future of ICT-mediated Elderly Care: A Response to Doom Scenarios. AI & SOCIETY 31(4):455–462. doi: 10.1007/s00146-015-0626-3

[36] Coghlan S (2022) Robots and the Possibility of Humanistic Care. International Journal of Social Robotics 14(10):2095–2108. doi: 10.1007/s12369-021-00804-7

[37] Coghlan S, Waycott J, Lazar A, et al (2021) Dignity, Autonomy, and Style of Company. Proceedings of the ACM on Human-Computer Interaction 5(CSCW1):1–25. doi: 10.1145/3449178

[38] Conti D, Di Nuovo S, Di Nuovo A (2021) A Brief Review of Robotics Technologies to Support Social Interventions for Older Users. In: Zimmermann A, Howlett RJ, Jain LC (eds) Human Centred Intelligent Systems, Smart Innovation, Systems and Technologies, vol 189. Springer Singapore, Singapore, p 221–232, doi: 10.1007/978-981-15-5784-2_18

[39] Decker M (2008) Caregiving robots and ethical reflection: the perspective of interdisciplinary technology assessment. AI & SOCIETY 22(3):315–330. doi: 10.1007/s00146-007-0151-0

[40] Depner D, Hülsken-Giesler M (2017) Robotik in der Pflege - Eckpunkte für eine prospektive ethische Bewertung in der Langzeitpflege. Personzentrierte Langzeitpflege doi: 10.14623/zfme.2017.1.51-62

[41] Deutscher Ethikrat (10. März 2020) Robotik für gute Pflege: Stellungnahme. Deutscher Ethikrat, Berlin

[42] Diaz-Orueta U, Hopper L, Konstantinidis E (2020) Shaping technologies for older adults with and without dementia: Reflections on ethics and preferences. Health Informatics Journal 26(4):3215–3230. doi: 10.1177/1460458219899590

[43] Döring N (2018) Sollten Pflegeroboter auch sexuelle Assistenzfunktionen bieten? In: Bendel O (ed) Pflegeroboter. Springer Fachmedien Wiesbaden, Wiesbaden, p 249–267, doi: 10.1007/978-3-658-22698-5_14

[44] Dosso JA, Bandari E, Malhotra A, et al (2022) User perspectives on emotionally aligned social robots for older adults and persons living with dementia. Journal of rehabilitation and assistive technologies engineering 9. doi: 10.1177/20556683221108364

[45] Draper H, Sorell T (2017) Ethical Values and Social Care Robots for Older People: An International Qualitative Study. Ethics and Information Technology 19(1):49–68. doi: 10.1007/s10676-016-9413-1

[46] Earp BD, Grunt-Mejer K (2021) Robots and sexual ethics. Journal of Medical Ethics 47(1):1–2. doi: 10.1136/medethics-2020-107153

[47] van Est R, Royakkers L (2016) Robotisation as Rationalisation – In Search for a Human Robot Future. Amsterdam University Press, doi: 10.25969/MEDIAREP/13393

[48] Espingardeiro A (2014) A Roboethics Framework for the Development and Introduction of Social Assistive Robots in Elderly Care. PhD thesis, University of Salford (United Kingdom)

[49] Feil-Seifer D, Matarić M (2011) Socially Assistive Robotics. IEEE Robotics & Automation Magazine 18(1):24–31. doi: 10.1109/MRA.2010.940150

[50] Felber NA, Pageau F, McLean A, et al (2022) The Concept of Social Dignity as a Yardstick to Delimit Ethical Use of Robotic Assistance in the Care of Older Persons. Medicine, Health Care & Philosophy 25(1):99–110. doi: 10.1007/s11019-021-10054-z

[51] Fiorini L, Rovini E, Russo S, et al (2022) On the Use of Assistive Technology during the COVID-19 Outbreak: Results and Lessons Learned from Pilot Studies. Sensors 22(17):6631. doi: 10.3390/s22176631

[52] Fosch-Villaronga E, Albo-Canals J (2019) "I'll take care of you," said the robot. Paladyn, Journal of Behavioral Robotics 10(1):77–93. doi: 10.1515/pjbr-2019-0006

[53] Fosch-Villaronga E, Poulsen A (2020) Sex care robots. Paladyn, Journal of Behavioral Robotics 11(1):1–18. doi: 10.1515/pjbr-2020-0001

[54] Frebel L (2015) Roboter gegen das Vergessen?: Technische Assistenz bei Altersdemenz Im Speilfilm aus medizinethischer Sicht. In: Weber K, Frommeld D, Manzeschke A, et al (eds) Technisierung Des Alltags: Beitrag Für Ein Gutes Leben? Franz Steiner, Stuttgart, p 99–116

[55] Frennert S, Östlund B (2014) Review: Seven Matters of Concern of Social Robots and Older People. International Journal of Social Robotics 6(2):299–310. doi: 10.1007/s12369-013-0225-8

[56] Früh M, Gasser A (2018) Erfahrungen aus dem Einsatz von Pflegerobotern für Menschen im Alter. In: Bendel O (ed) Pflegeroboter. Springer Fachmedien Wiesbaden, Wiesbaden, p 37–62, doi: 10.1007/978-3-658-22698-5_3

[57] Gallagher A, Nåden D, Karterud D (2016) Robots in elder care. Nursing Ethics 23(4):369–371. doi: 10.1177/0969733016647297

[58] Geier J, Mauch M, Patsch M, et al (2020) Wie Pflegekräfte im ambulanten Bereich den Einsatz von Telepräsenzsystemen einschätzen - Eine qualitative Studie. Pflege 33(1):43–51. doi: 10.1024/1012-5302/a000709

[59] Gelin R (2017) The Domestic Robot: Ethical and Technical Concerns. In: Aldinhas Ferreira MI, Silva Sequeira J, Tokhi MO, et al (eds) A World with Robots: International Conference on Robot Ethics: ICRE 2015. Springer International Publishing, Cham, p 207–216

[60] Giansanti D (2021) The Social Robot in Rehabilitation and Assistance: What Is the Future? Healthcare 9(3):244. doi: 10.3390/healthcare9030244

[61] Gisinger C (2018) Pflegeroboter aus Sicht der Geriatrie. In: Bendel O (ed) Pflegeroboter. Springer Fachmedien Wiesbaden, Wiesbaden, p 113–124, doi: 10.1007/978-3-658-22698-5_6

[62] Glende S, Conrad I, Krezdorn L, et al (2016) Increasing the Acceptance of Assistive Robots for Older People Through Marketing Strategies Based on Stakeholder Needs. International Journal of Social Robotics 8(3):355–369. doi: 10.1007/s12369-015-0328-5

[63] Gochoo M, Alnajjar F, Tan TH, et al (2021) Towards Privacy-Preserved Aging in Place: A Systematic Review. Sensors 21(9):3082. doi: 10.3390/s21093082

[64] Gräb-Schmidt E, Stritzelberger CP (2018) Ethische Herausforderungen durch autonome Systeme und Robotik im Bereich der Pflege. Zeitschrift für medizinische Ethik doi: 10.14623/zfme.2018.4.357-372

[65] Grunwald A, Kehl C (2020) Mit Robotern gegen den Pflegenotstand. Spektrum der Wissenschaft 20 Spezial Biologie Medizin Hirnforschung pp 16–17

[66] Haltaufderheide J, Lucht A, Strünck C, et al (2023) Socially Assistive Devices in Healthcare–a Systematic Review of Empirical Evidence from an Ethical Perspective. Science and Engineering Ethics 29(1). doi: 10.1007/s11948-022-00419-9

[67] Hasenauer R, Ehrenmueller I, Belviso C (2022) Living Labs in Social Service Institutions: An Effective Method to Improve the Ethical, Reliable Use of Digital Assistive Robots to Support Social Services. In: 2022 Portland International Conference on Management of Engineering and Technology (PICMET). IEEE, pp 1–9, doi: 10.23919/PICMET53225.2022.9882746

[68] Heeser AC (2020) Schöne digitale Welt. Pflegezeitschrift 73(9):10–12. doi: 10.1007/s41906-020-0776-x

[69] Hildt E (2019) Shaping the Development and Use of Intelligent Assistive Technologies in Dementia. In: Jotterand F, Ienca M, Wangmo T, et al (eds) Intelligent Assistive Technologies for Dementia. Oxford University PressNew York, p 130–144, doi: 10.1093/med/9780190459802.003.0008

[70] Hilgendorf E (2018) Recht und Ethik in der Pflegerobotik: Ein Überblick. Zeitschrift für medizinische Ethik doi: 10.14623/zfme.2018.4.373-385

[71] Honekamp I, Sauer L, Wache T, et al (2019) Akzeptanz von Pflegerobotern Im Krankenhaus: Eine Quantitative Studie. TATuP - Zeitschrift für Technikfolgenabschätzung in Theorie und Praxis 28(2):58–63. doi: 10.14512/tatup.28.2.s58

[72] Hoppe JA, Johansson-Pajala RM, Gustafsson C, et al (2020) Assistive robots in care: Expectations and perceptions of older people. In: Haltaufderheide J, Hovemann J, Vollmann J (eds) Aging between Participation and Simulation: Ethical Dimensions of Socially Assistive Technologies in Elderly Care. De Gruyter, p 139–156

[73] Hübner G, Müller S (2020) Roboter in der Pflege. Spektrum der Wissenschaft 20 Spezial Biologie Medizin Hirnforschung pp 6–8

[74] Hülsken-Giesler M, Daxberger S (2018) Robotik in der Pflege aus pflegewissenschaftlicher Perspektive. In: Bendel O (ed) Pflegeroboter. Springer Fachmedien Wiesbaden, Wiesbaden, p 125–139, doi: 10.1007/978-3-658-22698-5_7

[75] Hung L, Gregorio M, Mann J, et al (2021) Exploring the perceptions of people with dementia about the social robot PARO in a hospital setting. Dementia 20(2):485–504. doi: 10.1177/1471301219894141

[76] Hung L, Liu C, Woldum E, et al (2019) The Benefits of and Barriers to Using a Social Robot PARO in Care Settings: A Scoping Review. BMC geriatrics 19(1):1–10. doi: 10.1186/s12877-019-1244-6

[77] Hung L, Mann J, Perry J, et al (2022) Technological Risks and Ethical Implications of Using Robots in Long-Term Care. Journal of rehabilitation and assistive technologies engineering 9:1–10. doi: 10.1177/20556683221106917

[78] Huschilt J, Clune L (2012) The Use of Socially Assistive Robots for Dementia Care. Journal of Gerontological Nursing 38(10):15–19. doi: 10.3928/00989134-20120911-02

[79] Ienca M, Fabrice J, Elger B, et al (2017) Intelligent Assistive Technology for Alzheimer's Disease and Other Dementias: A Systematic Review. Journal of Alzheimer's Disease 56(4):1301–1340. doi: 10.3233/JAD-161037

[80] Ienca M, Jotterand F, Vică C, et al (2016) Social and Assistive Robotics in Dementia Care: Ethical Recommendations for Research and Practice. International Journal of Social Robotics 8(4):565–573. doi: 10.1007/s12369-016-0366-7

[81] Ienca M, Villaronga EF (2019) Privacy and Security Issues in Assistive Technologies for Dementia. In: Jotterand F, Ienca M, Wangmo T, et al (eds) Intelligent Assistive Technologies for Dementia. Oxford University PressNew York, p 221–239, doi: 10.1093/med/9780190459802.003.0013

[82] Isabet B, Pino M, Lewis M, et al (2021) Social Telepresence Robots: A Narrative Review of Experiments Involving Older Adults before and during the COVID-19 Pandemic. International Journal of Environmental Research and Public Health 18(7):3597. doi: 10.3390/ijerph18073597
[83] Jecker NS (2021) Nothing to be ashamed of: sex robots for older adults with disabilities. Journal of Medical Ethics 47(1):26–32. doi: 10.1136/medethics-2020-106645
[84] Jecker NS (2021) Sex robots for older adults with disabilities: reply to critics. Journal of Medical Ethics 47(2):113–114. doi: 10.1136/medethics-2020-107148
[85] Jecker N (2021) You've Got a Friend in Me: Sociable Robots for Older Adults in an Age of Global Pandemics. Ethics and Information Technology 23(S1):35–43. doi: 10.1007/s10676-020-09546-y
[86] Jenkins S, Draper H (2015) Care, Monitoring, and Companionship: Views on Care Robots from Older People and Their Carers. International Journal of Social Robotics 7(5):673–683. doi: 10.1007/s12369-015-0322-y
[87] Johansson-Pajala RM, Gustafsson C (2022) Significant challenges when introducing care robots in Swedish elder care. Disability and Rehabilitation: Assistive Technology 17(2):166–176. doi: 10.1080/17483107.2020.1773549
[88] Johansson-Pajala RM, Thommes K, Hoppe JA, et al (2020) Care Robot Orientation: What, Who and How? Potential Users' Perceptions. International Journal of Social Robotics 12(5):1103–1117. doi: 10.1007/s12369-020-00619-y
[89] Johnston C (2022) Ethical Design and Use of Robotic Care of the Elderly. Journal of Bioethical Inquiry 19(1):11–14. doi: 10.1007/s11673-022-10181-z
[90] Kamphof I (2015) In the Company of Robots: Health Care and the Identity of People with Dementia. In: Swinnen A, Schweda M (eds) Popularizing Dementia: Public Expressions and Representations of Forgetfulness. Transcript, Bielefeld, p 359–376, doi: 10.1515/9783839427101-017
[91] Kayser D, Gasser A, Früh M (2022) F&P Robotics, Assistenzroboter Lio. Ein Erfahrungsbericht. In: Stronegger W, Platzer J (eds) Technisierung der Pflege: 4. Goldegger Dialogforum Mensch und Endlichkeit. Bioethik in Wissenschaft und Gesellschaft, Nomos, Baden-Baden, p 67–78
[92] Kehl C (2018) Robotik und assistive Neurotechnologien in der Pflege - gesellschaftliche Herausforderungen. Vertiefung des Projekts Mensch-Maschine-Entgrenzungen: TAB Arbeitsbericht. doi: 10.5445/IR/1000094095
[93] Kehl C (2018) Wege zu verantwortungsvoller Forschung und Entwicklung im Bereich der Pflegerobotik: Die ambivalente Rolle der Ethik. In: Bendel O (ed) Pflegeroboter. Springer Fachmedien Wiesbaden, Wiesbaden, p 141–160, doi: 10.1007/978-3-658-22698-5_1
[94] Kehl C (2018) Entgrenzungen zwischen Mensch und Maschine, Oder: Können Roboter zu guter Pflege beitragen? Aus Politik und Zeitgeschichte 68(6-8):22–28
[95] Keibel A (2020) Warum tut sich die Pflegerobotik so schwer? Spektrum der Wissenschaft 20 Spezial Biologie Medizin Hirnforschung pp 12–15
[96] van Kemenade MAM, Hoorn JF, Konijn EA (2018) Healthcare Students' Ethical Considerations of Care Robots in The Netherlands. Applied Sciences 8(10):1712. doi: 10.3390/app8101712
[97] van Kemenade MAM, Hoorn JF, Konijn EA (2019) Do You Care for Robots That Care? Exploring the Opinions of Vocational Care Students on the Use of Healthcare Robots. Robotics 8(1):22. doi: 10.3390/robotics8010022
[98] Kenigsberg PA, Aquino JP, Bérard A, et al (2019) Assistive Technologies to Address Capabilities of People with Dementia: From Research to Practice. Dementia 18(4):1568–1595. doi: 10.1177/1471301217714093
[99] Khaksar W, Saplacan D, Bygrave LA, et al (2023) Robotics in Elderly Healthcare: A Review of 20 Recent Research Projects. doi: 10.48550/arXiv.2302.04478
[100] Kim JW, Choi YL, Jeong SH, et al (2022) A Care Robot with Ethical Sensing System for Older Adults at Home. Sensors 22(19):7515. doi: 10.3390/s22197515
[101] Klebbe R, Klüber K, Dahms R, et al (2023) Caregivers' Perspectives on Human–Robot Collaboration in Inpatient Elderly Care Settings. Machines 11(1):34. doi: 10.3390/machines11010034

[102] Kodate N, Maeda Y, Hauray B, et al (2022) Hopes and fears regarding care robots: Content analysis of newspapers in East Asia and Western Europe, 2001–2020. Frontiers in Rehabilitation Sciences 3. doi: 10.3389/fresc.2022.1019089

[103] Kodate N, Donnelly S, Suwa S, et al (2022) Home-care robots – Attitudes and perceptions among older people, carers and care professionals in Ireland: A questionnaire study. Health & Social Care in the Community 30(3):1086–1096. doi: 10.1111/hsc.13327

[104] Koeszegi S, Weiss Astrid (2021) Mein neuer Teamkollege ist ein Roboter! Wie soziale Roboter die Zukunft der Arbeit verändern können. In: Altenburger R, Schmidpeter R (eds) CSR und Künstliche Intelligenz. Springer Gabler, Berlin and Heidelberg, p 279–303

[105] Koh WQ, Ang FXH, Casey D (2021) Impacts of Low-cost Robotic Pets for Older Adults and People With Dementia: Scoping Review. JMIR Rehabilitation and Assistive Technologies 8(1):e25340. doi: 10.2196/25340

[106] Kohlen H (2021) Sorge-Ethik als menschliche Praxis im Unterschied zu Technik und Robotik in der Pflege. Imago Hominis 28:129–135

[107] Koimizu J (2019) Aged Care with Socially Assistive Robotics under Advance Care Planning. In: 2019 IEEE International Conference on Advanced Robotics and its Social Impacts (ARSO). IEEE, pp 34–38, doi: 10.1109/ARSO46408.2019.8948742

[108] Körtner T (2016) Ethical challenges in the use of social service robots for elderly people. Zeitschrift für Gerontologie und Geriatrie 49(4):303–307. doi: 10.1007/s00391-016-1066-5

[109] Koumpis A, Gees T (2020) Sex with Robots: A Not-so-Niche Market for Disabled and Older Persons. Paladyn: Journal of Behavioral Robotics 11(1):228–232. doi: 10.1515/pjbr-2020-0009

[110] Kovács L (2021) Anthropologische Und Ethische Aspekte Des Einsatzes von Robotern Im Gesundheitssektor. In: Inthorn J, Seising R (eds) Digitale Patientenversorgung: Zur Computerisierung von Diagnostik, Therapie Und Pflege, Medical Humanities, vol 3. transcript Verlag, Bielefeld, p 19–34

[111] Kreis J (2018) Umsorgen, überwachen, unterhalten - sind Pflegeroboter ethisch vertretbar? In: Bendel O (ed) Pflegeroboter. Springer Fachmedien Wiesbaden, Wiesbaden, p 213–228, doi: 10.1007/978-3-658-22698-5_12

[112] Lancaster K (2023) Granny and the Sexbots: An ethical appraisal of the use of sexbots in residential care institutions for elderly people. In: Loh J, Loh W (eds) Social robotics and the good life. Philosophy, transcript Verlag, Bielefeld, p 181–207

[113] Lange N, Bauer L (2021) Eine Robbe für Oma - Die zukünftige Dauerausstellung Robotik im Deutschen Museum. In: Inthorn J, Seising R (eds) Digitale Patientenversorgung: Zur Computerisierung von Diagnostik, Therapie Und Pflege. Medical Humanities, transcript Verlag, Bielefeld, p 221–240

[114] Lau YY, van't Hof C, van Est R (2009) R&D in healthcare robotics. In: Lau YY, van't Hof C, van Est R (eds) Beyond the surface. Technology assessment, Rathenau Institute, The Hague, p 17–34

[115] Lau YY, van't Hof C, van Est R (2009) Robotics for an aging Japanese society. In: Lau YY, van't Hof C, van Est R (eds) Beyond the surface. Technology assessment, Rathenau Institute, The Hague, p 9–16

[116] Lee H, Chung MA, Kim H, et al (2022) The Effect of Cognitive Function Health Care Using Artificial Intelligence Robots for Older Adults: Systematic Review and Meta-analysis. JMIR Aging 5(2):e38896. doi: 10.2196/38896

[117] Lehmann S, Ruf E, Misoch S (2020) Robot Use for Older Adults – Attitudes, Wishes and Concerns. First Results from Switzerland. In: Stephanidis C, Antona M (eds) HCI International 2020 - Posters, Communications in Computer and Information Science, vol 1226. Springer International Publishing, Cham, p 64–70, doi: 10.1007/978-3-030-50732-9_9

[118] Lehmann S, Ruf E, Misoch S (2021) Using a Socially Assistive Robot in a Nursing Home: Caregivers' Expectations and Concerns. In: Stephanidis C, Antona M, Ntoa S (eds) HCI International 2021 - Posters, Communications in Computer and Information Science, vol 1420. Springer International Publishing, Cham, p 148–155, doi: 10.1007/978-3-030-78642-7_20

[119] Lehoux P, Grimard D (2018) When robots care: Public deliberations on how technology and humans may support independent living for older adults. Social Science & Medicine 211:330–337. doi: 10.1016/j.socscimed.2018.06.038

[120] Li S, van Wynsberghe A, Roeser S (2020) The Complexity of Autonomy: A Consideration of the Impacts of Care Robots on the Autonomy of Elderly Care Receivers. In: Nørskov M, Seibt J, Quick OS (eds) Culturally Sustainable Social Robotics: Proceedings of Robophilosophy 2020. IOS Press, Amsterdam, Berlin, Washington, DC, p 316–325, doi: 10.3233/FAIA200928

[121] Liang A, Piroth I, Robinson H, et al (2017) A Pilot Randomized Trial of a Companion Robot for People With Dementia Living in the Community. Journal of the American Medical Directors Association 18(10):871–878. doi: 10.1016/j.jamda.2017.05.019

[122] Mansouri N, Goher K, Hosseini SE (2017) Ethical framework of assistive devices: review and reflection. Robotics and Biomimetics 4(1). doi: 10.1186/s40638-017-0074-2

[123] Manzeschke A (2019) Roboter in der Pflege: Von Menschen, Maschinen und anderen hilfreichen Wesen. EthikJournal 2019(1):1–11

[124] Manzeschke A (2022) Robotik in der Pflege? Ethische Merkposten für ihren Einsatz. Public Health Forum 30(1):41–43. doi: 10.1515/pubhef-2021-0123

[125] Manzeschke A, Petersen J (2020) Digitalisierung und Roboterisierung in der Pflege: Ethisch-anthropologische Überlegungen. In: Dibelius O, Piechotta-Henze G (eds) Menschenrechts-basierte Pflege. Hogrefe, Bern, p 161–173

[126] Marchang J, Di Nuovo A (2022) Assistive Multimodal Robotic System (AMRSys): Security and Privacy Issues, Challenges, and Possible Solutions. Applied Sciences 12(4):1–29. doi: 10.3390/app12042174

[127] van Maris A, Zook N, Caleb-Solly P, et al (2020) Designing Ethical Social Robots–A Longitudinal Field Study With Older Adults. Frontiers in Robotics and AI 7:1–14. doi: 10.3389/frobt.2020.00001

[128] van Maris A, Zook N, Dogramadzi S, et al (2021) A New Perspective on Robot Ethics through Investigating Human–Robot Interactions with Older Adults. Applied Sciences 11(21):10136. doi: 10.3390/app112110136

[129] Martens R, Hildebrand C (2021) Dementia care, robot pets, and aliefs. Bioethics 35(9):870–876. doi: 10.1111/bioe.12952

[130] Matarić M, Scassellati B (2016) Socially Assistive Robotics. In: Siciliano B, Khatib O (eds) Springer Handbook of Robotics. Springer Handbooks, Springer, doi: 10.1007/978-3-319-32552-1

[131] Matthias A (2015) Robot Lies in Health Care: When Is Deception Morally Permissible? Kennedy Institute of Ethics Journal 25(2):169–162. doi: 10.1353/ken.2015.0007

[132] von Maur I (2023) Alice Does Not Care: Or: Why It Matters That Robots "Don't Give a Damn". In: Loh J, Loh W (eds) Social Robotics and the Good Life: The Normative Side of Forming Emotional Bonds With Robots. transcript Verlag, Bielefeld, p 209–231

[133] Meacham D, Studley M (2017) Could a Robot Care? It's All in the Movement. In: Abney K, Lin P, Jenkins R (eds) Robot ethics 2.0. Oxford University Press, New York, NY, p 97–113

[134] Meiland F, Innes A, Mountain G, et al (2017) Technologies to Support Community-Dwelling Persons With Dementia: A Position Paper on Issues Regarding Development, Usability, Effectiveness and Cost-Effectiveness, Deployment, and Ethics. JMIR Rehabilitation and Assistive Technologies 4(1):e1. doi: 10.2196/rehab.6376

[135] Metzler TA, Barnes SJ (2014) Three dialogues concerning robots in elder care. Nursing Philosophy 15(1):4–13. doi: 10.1111/nup.12027

[136] Metzler TA, Lewis LM, Pope LC (2016) Could robots become authentic companions in nursing care? Nursing Philosophy 17(1):36–48. doi: 10.1111/nup.12101

[137] Meyer S (2011) Akzeptanz ausgewählter Anwendungsszenarien. In: Mein Freund der Roboter: Servicerobotik für ältere Menschen - eine Antwort auf den demografischen Wandel? VDE-Verlag, Berlin, Offenbach, p 59–103

[138] Meyer S (2011) Akzeptanzbedingungen für Roboter-Assistenten. In: Mein Freund der Roboter: Servicerobotik für ältere Menschen - eine Antwort auf den demografischen Wandel? VDE-Verlag, Berlin, Offenbach, p 104–113

[139] Mishra N, Tulsulkar G, Li H, et al (2021) Does Elderly Enjoy Playing Bingo with a Robot? A Case Study with the Humanoid Robot Nadine. In: Magnenat-Thalmann N, Interrante V, Thalmann D, et al (eds) Advances in Computer Graphics, Lecture Notes in Computer Science, vol 13002. Springer International Publishing, Cham, p 491–503, doi: 10.1007/978-3-030-89029-2_38

[140] Misselhorn C (2018) Pflegesysteme. In: Grundfragen Der Maschinenethik. Reclams Universal Bibliothek, Reclam, Ditzingen, p 136–154
[141] Misselhorn C (2019) Moralische Maschinen in der Pflege? Grundlagen und eine Roadmap für ein moralisch lernfähiges Altenpflegesystem. In: Woopen C, Jannes M (eds) Roboter in der Gesellschaft. Springer Berlin Heidelberg, Berlin, Heidelberg, p 53–68, doi: 10.1007/978-3-662-57765-3_4
[142] Misselhorn C (2021) Von empathischen Pflegerobotern und virtuellen Seelenklempnern. In: Misselhorn C (ed) Künstliche Intelligenz und Empathie. /Was bedeutet das alles?], Reclam, Ditzingen, p 61–74
[143] Misselhorn C, Pompe U, Stapleton M (2013) Ethical Considerations Regarding the Use of Social Robots in the Fourth Age. GeroPsych 26(2):121–133. doi: 10.1024/1662-9647/a000088
[144] Moyle W, Jones C, Murfield J, et al (2019) Using a therapeutic companion robot for dementia symptoms in long-term care: reflections from a cluster-RCT. Aging & Mental Health 23(3):329–336. doi: 10.1080/13607863.2017.1421617
[145] Nass E, Schneider M (2022) Maschinen mit Moral für eine gute Pflege der Zukunft? In: Pfannstiel M (ed) Künstliche Intelligenz im Gesundheitswesen: Entwicklungen, Beispiele und Perspektiven. Springer Gabler, Wiesbaden, p 311–323
[146] Navon M (2021) The Virtuous Servant Owner—A Paradigm Whose Time has Come (Again). Frontiers in Robotics and AI 8. doi: 10.3389/frobt.2021.715849
[147] Nestorov N, Stone E, Lehane P, et al (2014) Aspects of Socially Assistive Robots Design for Dementia Care. In: 2014 IEEE 27th International Symposium on Computer-Based Medical Systems. IEEE, pp 396–400, doi: 10.1109/CBMS.2014.16
[148] Nielsen S, Langensiepen S, Madi M, et al (2022) Implementing ethical aspects in the development of a robotic system for nursing care: a qualitative approach. BMC Nursing 21(1). doi: 10.1186/s12912-022-00959-2
[149] Niemelä M, van Aerschot L, Tammela A, et al (2021) Towards Ethical Guidelines of Using Telepresence Robots in Residential Care. International Journal of Social Robotics 13:431–439. doi: 10.1007/s12369-019-00529-8
[150] Noori FM, Uddin Z, Torresen J (2019) Robot-Care for the Older People: Ethically Justified or Not? In: 2019 Joint IEEE 9th International Conference on Development and Learning and Epigenetic Robotics (ICDL-EpiRob). IEEE, pp 43–47, doi: 10.1109/DEVLRN.2019.8850706
[151] Nordgren A (2018) How to respond to resistiveness towards assistive technologies among persons with dementia. Medicine, Health Care and Philosophy 21(3):411–421. doi: 10.1007/s11019-017-9816-8
[152] Nyholm L, Santamäki-Fischer R, Fagerström L (2021) Users' ambivalent sense of security with humanoid robots in healthcare. Informatics for Health and Social Care 46(2):218–226. doi: 10.1080/17538157.2021.1883027
[153] O'Brolcháin F (2019) Robots and people with dementia: Unintended consequences and moral hazard. Nursing Ethics 26(4):962–972. doi: 10.1177/0969733017742960
[154] Paganini C (2022) Mit Kranken, Hochbetagten und Sterbenden kommunizieren. Sollen Pflegeroboter immer die Wahrheit sagen? In: Stronegger W, Platzer J (eds) Technisierung der Pflege: 4. Goldegger Dialogforum Mensch und Endlichkeit. Bioethik in Wissenschaft und Gesellschaft, Nomos, Baden-Baden, p 91–106
[155] Paletta L, Schüssler S, Zuschnegg J, et al (2019) AMIGO–A Socially Assistive Robot for Coaching Multimodal Training of Persons with Dementia. In: Korn O (ed) Social Robots: Technological, Societal and Ethical Aspects of Human-Robot Interaction. Human-Computer Interaction Series, Springer, Cham, p 265–284, doi: 10.1007/978-3-030-17107-0
[156] Parviainen J, Turja T, van Aerschot L (2019) Social Robots and Human Touch in Care: The Perceived Usefulness of Robot Assistance Among Healthcare Professionals. In: Korn O (ed) Social Robots: Technological, Societal and Ethical Aspects of Human-Robot Interaction. Human-Computer Interaction Series, Springer, Cham, p 187–204, doi: 10.1007/978-3-030-17107-0
[157] van Patten R, Keller AV, Maye JE, et al (2020) Home-Based Cognitively Assistive Robots: Maximizing Cognitive Functioning and Maintaining Independence in Older Adults Without Dementia. Clinical interventions in aging 15:1129–1139. doi: 10.2147/CIA.S253236

[158] Pilotto A, Boi R, Petermans J (2018) Technology in geriatrics. Age and Ageing 47(6):771–774. doi: 10.1093/ageing/afy026

[159] Pino M, Boulay M, Jouen F, et al (2015) ”Are we ready for robots that care for us?” Attitudes and opinions of older adults toward socially assistive robots. Frontiers in aging neuroscience 7:141. doi: 10.3389/fnagi.2015.00141

[160] Pirhonen J, Melkas H, Laitinen A, et al (2020) Could robots strengthen the sense of autonomy of older people residing in assisted living facilities?—A future-oriented study. Ethics and Information Technology 22(2):151–162. doi: 10.1007/s10676-019-09524-z

[161] Pirhonen J, Tiilikainen E, Pekkarinen S, et al (2020) Can Robots Tackle Late-Life Loneliness? Scanning of Future Opportunities and Challenges in Assisted Living Facilities. Futures 124:1–12. doi: 10.1016/j.futures.2020.102640

[162] Poulsen A, Burmeister OK (2019) Overcoming carer shortages with care robots: Dynamic value trade-offs in run-time. Australasian Journal of Information Systems 23. doi: 10.3127/ajis.v23i0.1688

[163] Poulsen A, Fosch-Villaronga E, Burmeister OK (2020) Cybersecurity, value sensing robots for LGBTIQ+ elderly, and the need for revised codes of conduct. Australasian Journal of Information Systems 24. doi: 10.3127/ajis.v24i0.2789

[164] Preuß D, Legal F (2017) Living with the animals: animal or robotic companions for the elderly in smart homes? Journal of Medical Ethics 43(6):407–410. doi: 10.1136/medethics-2016-103603

[165] Radic M, Vosen A (2020) Ethische, rechtliche und soziale Anforderungen an Assistenzroboter in der Pflege. Zeitschrift für Gerontologie und Geriatrie 53(7):630–636. doi: 10.1007/s00391-020-01791-6

[166] Rantanen T, Lehto P, Vuorinen P, et al (2018) The adoption of care robots in home care—A survey on the attitudes of Finnish home care personnel. Journal of Clinical Nursing 27(9-10):1846–1859. doi: 10.1111/jocn.14355

[167] Reiß T (2019) Editorial. EthikJournal 5(1):1–5

[168] Remmers H (2018) Pflegeroboter: Analyse und Bewertung aus Sicht pflegerischen Handelns und ethischer Anforderungen. In: Bendel O (ed) Pflegeroboter. Springer Fachmedien Wiesbaden, Wiesbaden, p 161–179, doi: 10.1007/978-3-658-22698-5_9

[169] Remmers H (2019) Pflege und Technik. Stand der Diskussion und zentrale ethische Fragen. Ethik in der Medizin 31(4):407–430. doi: 10.1007/s00481-019-00545-2

[170] Robillard JM, Kabacińska K (2020) Realizing the Potential of Robotics for Aged Care Through Co-Creation. Journal of Alzheimer's Disease 76(2):461–466. doi: 10.3233/JAD-200214

[171] Ruf E, Lehmann S, Misoch S (2021) Ethical Concerns of the General Public regarding the Use of Robots for Older Adults. In: Proceedings of the 7th International Conference on Information and Communication Technologies for Ageing Well and e-Health. SCITEPRESS - Science and Technology Publications, pp 221–227, doi: 10.5220/0010478202210227

[172] Ruf E, Lehmann S, Pauli C, et al (2020) Roboter Zur Unterstützung Im Alter. HMD Praxis der Wirtschaftsinformatik 57(6):1251–1270. doi: 10.1365/s40702-020-00681-0

[173] Sahm S (2019) Digitale Anthropologie: Ethische Probleme der Anwendung künstlicher Intelligenz und Robotik in der Pflege und Medizin. Medizinrecht 37(12):927–933. doi: 10.1007/s00350-019-5395-4

[174] Salvini P (2015) On Ethical, Legal and Social Issues of Care Robots. In: Mohammed S, Moreno J, Kong K, et al (eds) Intelligent Assistive Robots: Recent Advances in Assistive Robotics for Everyday Activities. Springer Tracts in Advanced Robotics, Springer International Publishing, Cham, p 431–445, doi: 10.1007/978-3-319-12922-8

[175] Saplacan D, Khaksar W, Torresen J (2021) On Ethical Challenges Raised by Care Robots: A Review of the Existing Regulatory-, Theoretical-, and Research Gaps. In: 2021 IEEE International Conference on Advanced Robotics and Its Social Impacts (ARSO). IEEE, pp 219–226, doi: 10.1109/ARSO51874.2021.9542844

[176] Schicktanz S, Schweda M (2021) Aging 4.0? Rethinking the ethical framing of technology-assisted eldercare. History and Philosophy of the Life Sciences 43(3). doi: 10.1007/s40656-021-00447-x

[177] Schmidhuber M (2022) Werden Roboter Menschen in der Pflege ersetzen? Ethische Überlegungen. In: Stronegger W, Platzer J (eds) Technisierung der Pflege: 4. Goldegger

Dialogforum Mensch und Endlichkeit. Bioethik in Wissenschaft und Gesellschaft, Nomos, Baden-Baden, p 167–174, doi: 10.5771/9783748928720
[178] Schmidhuber M, Stöger K (2021) Ethisches und Rechtliches zur Zukunft der Robotik in der Pflege: Grundfragen für österreichische und deutsche Debatten. In: Die Zukunft von Medizin und Gesundheitswesen. Königshausen & Neumann, 2021, Würzburg
[179] Schmietow, Bettina (2020) Reconfigurations of autonomy in digital health and the ethics of (socially) assistive technologies. In: Haltaufderheide J, Hovemann J, Vollmann J (eds) Aging between Participation and Simulation: Ethical Dimensions of Socially Assistive Technologies in Elderly Care. De Gruyter, p 171–184
[180] Schwaninger I (2020) Practice-Based Trust Research: Towards Situated Human-Robot Interaction in Older People's Living Spaces. In: Nørskov M, Seibt J, Quick OS (eds) Culturally Sustainable Social Robotics. Frontiers in Artificial Intelligence and Applications, IOS Press, doi: 10.3233/FAIA200975
[181] Scorna U (2015) Servicerobotik in der Altenpflege: Eine empirische Untersuchung des Einsatzes der Serviceroboter in der stationären Altenpflege am Beispiel von PARO und Care-O-bot. In: Technisierung des Alltags. Franz Steiner Verlag, 2015, Stuttgart
[182] Sedenberg E, Chuang J, Mulligan D (2016) Designing Commercial Therapeutic Robots for Privacy Preserving Systems and Ethical Research Practices Within the Home. International Journal of Social Robotics 8(4):575–587. doi: 10.1007/s12369-016-0362-y
[183] Seefeldt D, Hülsken-Giesler M (2020) Pflegeethik und Robotik in der Pflege. In: Monteverde S (ed) Handbuch Pflegeethik. Pflegepraxis, Verlag W. Kohlhammer, Stuttgart, p 271–284
[184] Segers S (2022) Robot Technology for the Elderly and the Value of Veracity: Disruptive Technology or Reinvigorating Entrenched Principles? Science and Engineering Ethics 28(6). doi: 10.1007/s11948-022-00420-2
[185] Servaty R, Kersten A, Brukamp K, et al (2020) Implementation of robotic devices in nursing care. Barriers and facilitators: an integrative review. BMJ Open 10(9):e038650. doi: 10.1136/bmjopen-2020-038650
[186] Sharkey A (2014) Robots and human dignity: a consideration of the effects of robot care on the dignity of older people. Ethics and Information Technology 16(1):63–75. doi: 10.1007/s10676-014-9338-5
[187] Sharkey A, Sharkey N (2012) Granny and the robots: ethical issues in robot care for the elderly. Ethics and Information Technology 14(1):27–40. doi: 10.1007/s10676-010-9234-6
[188] Sharkey A, Sharkey N (2011) Children, the Elderly, and Interactive Robots. IEEE Robotics & Automation Magazine 18(1):32–38. doi: 10.1109/MRA.2010.940151
[189] Sharkey N (2008) The Ethical Frontiers of Robotics. Science 322(5909):1800–1801. doi: 10.1126/science.1164582
[190] Sharkey N, Sharkey A (2014) The Rights and Wrongs of Robot Care. In: Lin P, Abney K, Bekey G (eds) Robot Ethics: The Ethical and Social Implications of Robotics. MIT Press, Cambridge, p 267–282
[191] Sharkey N, Sharkey A (2012) The Eldercare Factory. Gerontology 58(3):282–288. doi: 10.1159/000329483
[192] Sharts-Hopko NC (2014) The Coming Revolution in Personal Care Robotics. Nursing Administration Quarterly 38(1):5–12. doi: 10.1097/NAQ.0000000000000000
[193] Shelton BE, Uz C (2015) Immersive Technology and the Elderly: A Mini-Review. Gerontology 61(2):175–185. doi: 10.1159/000365754
[194] Shim J, Arkin RC (2016) Other-Oriented Robot Deception: How Can a Robot's Deceptive Feedback Help Humans in HRI? In: Agah A, Cabibihan JJ, Howard AM, et al (eds) Social Robotics, Lecture Notes in Computer Science, vol 9979. Springer International Publishing, Cham, p 222–232, doi: 10.1007/978-3-319-47437-3_22
[195] Sorell T, Draper H (2014) Robot carers, ethics, and older people. Ethics and Information Technology 16(3):183–195. doi: 10.1007/s10676-014-9344-7
[196] Sparrow R (2002) The March of the Robot Dogs. Ethics and Information Technology 4(4):305–318. doi: 10.1023/A:1021386708994
[197] Sparrow R (2016) Robots in Aged Care: A Dystopian Future? AI & SOCIETY 31(4):445–454. doi: 10.1007/s00146-015-0625-4

[198] Sparrow R (2021) Sex robot fantasies. Journal of Medical Ethics 47(1):33–34. doi: 10.1136/medethics-2020-106932
[199] Sriram V, Jenkinson C, Peters M (2019) Informal carers' experience of assistive technology use in dementia care at home: a systematic review. BMC Geriatrics 19(1). doi: 10.1186/s12877-019-1169-0
[200] Steinrötter B (2020) Personal Robots in Der Pflege. In: Ebers M, Heinze C, Krügel T, et al (eds) Künstliche Intelligenz Und Robotik: Rechtshandbuch. Verlag C.H.BECK, München, p 789–827
[201] Street J, Barrie H, Eliott J, et al (2022) Older Adults' Perspectives of Smart Technologies to Support Aging at Home: Insights from Five World Café Forums. International Journal of Environmental Research and Public Health 19(13):7817. doi: 10.3390/ijerph19137817
[202] Strünck C, Reuter V, Gerling V, et al (2022) Socially assistive robots on the market. Zeitschrift für Gerontologie und Geriatrie 55(5):376–380. doi: 10.1007/s00391-022-02087-7
[203] Suwa S, Tsujimura M, Ide H, et al (2020) Home-Care Professionals' Ethical Perceptions of the Development and Use of Home-care Robots for Older Adults in Japan. International Journal of Human-Computer Interaction 36(14):1295–1303. doi: 10.1080/10447318.2020.1736809
[204] The Swedish National Council on Medical Ethics (2017) Robots and Surveillance in Health Care of the Elderly – Ethical Aspects. Jahrbuch für Wissenschaft und Ethik 21(1):445–452. doi: 10.1515/jwiet-2017-0125
[205] Tan SY, Taeihagh A, Tripathi A (2021) Tensions and Antagonistic Interactions of Risks and Ethics of Using Robotics and Autonomous Systems in Long-Term Care. Technological Forecasting and Social Change 167:1–15. doi: 10.1016/j.techfore.2021.120686
[206] Tanioka T (2019) Nursing and Rehabilitative Care of the Elderly Using Humanoid Robots. The Journal of Medical Investigation 66(1.2):19–23. doi: 10.2152/jmi.66.19
[207] Teo Y (2021) Recognition, collaboration and community: science fiction representations of robot carers in Robot & Frank , Big Hero 6 and Humans. Medical Humanities 47(1):95–102. doi: 10.1136/medhum-2019-011744
[208] Thalmann NM (2022) Social Robots: Their History and What They Can Do for Us. In: Werthner H, Prem E, Lee E, et al (eds) Perspectives on Digital Humanism. Springer International Publishing, Cham, p 9–17, doi: 10.1007/978-3-030-86144-5_2
[209] Tørresen J (2021) Undertaking Research with Humans within Artificial Intelligence and Robotics: Multimodal Elderly Care Systems. Technology—Architecture + Design 5(2):141–145. doi: 10.1080/24751448.2021.1967052
[210] Tørresen J, Kurazume R, Prestes E (2020) Special Issue on Elderly Care Robotics – Technology and Ethics. Journal of Intelligent & Robotic Systems 98(1):3–4. doi: 10.1007/s10846-020-01148-6
[211] Turja T, Taipale S, Niemelä M, et al (2022) Positive Turn in Elder-Care Workers' Views Toward Telecare Robots. International Journal of Social Robotics 14(4):931–944. doi: 10.1007/s12369-021-00841-2
[212] Tzafestas SG (2016) Roboethics, vol 79. Springer International Publishing, Cham, doi: 10.1007/978-3-319-21714-7
[213] Umbrello S, Capasso M, Balistreri M, et al (2021) Value Sensitive Design to Achieve the UN SDGs with AI: A Case of Elderly Care Robots. Minds and Machines 31(3):395–419. doi: 10.1007/s11023-021-09561-y
[214] Vandemeulebroucke T, de Casterlé BD, Gastmans C (2018) How do older adults experience and perceive socially assistive robots in aged care: a systematic review of qualitative evidence. Aging & Mental Health 22(2):149–167. doi: 10.1080/13607863.2017.1286455
[215] Vandemeulebroucke T, Dierckx de Casterlé B, Gastmans C (2018) The Use of Care Robots in Aged Care: A Systematic Review of Argument-Based Ethics Literature. Archives of Gerontology and Geriatrics 74:15–25. doi: 10.1016/j.archger.2017.08.014
[216] Vandemeulebroucke T, Dierckx de Casterlé B, Gastmans C (2020) Ethics of socially assistive robots in aged-care settings: a socio-historical contextualisation. Journal of Medical Ethics 46(2):128–136. doi: 10.1136/medethics-2019-105615
[217] Vandemeulebroucke T, Dierckx de Casterlé B, Gastmans C (2021) Socially Assistive Robots in Aged Care: Ethical Orientations Beyond the Care-Romantic and Technology-Deterministic Gaze. Science and Engineering Ethics 27(2). doi: 10.1007/s11948-021-00296-8

[218] Vandemeulebroucke T, Dierckx de Casterlé B, Welbergen L, et al (2020) The Ethics of Socially Assistive Robots in Aged Care. A Focus Group Study With Older Adults in Flanders, Belgium. The Journals of Gerontology: Series B 75(9):1996–2007. doi: 10.1093/geronb/gbz070

[219] Vandemeulebroucke T, Dzi K, Gastmans C (2021) Older adults' experiences with and perceptions of the use of socially assistive robots in aged care: A systematic review of quantitative evidence. Archives of Gerontology and Geriatrics 95:104399. doi: 10.1016/j.archger.2021.104399

[220] Vercelli A, Rainero I, Ciferri L, et al (2018) Robots in Elderly Care. DigitCult@Scientific Journal on Digital Cultures 2:37–50

[221] Vollmer Dahlke D, Ory MG (2020) Emerging Issues of Intelligent Assistive Technology Use Among People With Dementia and Their Caregivers: A U.S. Perspective. Frontiers in Public Health 8. doi: 10.3389/fpubh.2020.00191

[222] Wachsmuth I (2018) Robots Like Me: Challenges and Ethical Issues in Aged Care. Frontiers in Psychology 9(432):1–3. doi: 10.3389/fpsyg.2018.00432

[223] Wada K, Shibata T (2007) Living With Seal Robots–Its Sociopsychological and Physiological Influences on the Elderly at a Care House. IEEE Transactions on Robotics 23(5):972–980. doi: 10.1109/TRO.2007.906261

[224] Wagner E, Borycki EM (2022) The Use of Robotics in Dementia Care: An Ethical Perspective. In: Mantas J, Hasman A, Househ MS, et al (eds) Informatics and Technology in Clinical Care and Public Health. Studies in Health Technology and Informatics, IOS Press, doi: 10.3233/SHTI210934

[225] Wahl HW, Mombaur K, Schubert A (2021) Robotik und Altenpflege: Freund oder Feind? Pflegezeitschrift 74(11):62–66. doi: 10.1007/s41906-021-1156-x

[226] Walters ML, Koay KL, Syrdal DS, et al (2013) Companion robots for elderly people: Using theatre to investigate potential users' views. In: 2013 IEEE RO-MAN. IEEE, pp 691–696, doi: 10.1109/ROMAN.2013.6628393

[227] Wang J, Liu T, Liu Z, et al (2019) Affective Interaction Technology of Companion Robots for the Elderly: A Review. In: El Rhalibi A, Pan Z, Jin H, et al (eds) E-Learning and Games, Lecture Notes in Computer Science, vol 11462. Springer International Publishing, Cham, p 79–83, doi: 10.1007/978-3-030-23712-7_11

[228] Wangmo T, Lipps M, Kressig RW, et al (2019) Ethical concerns with the use of intelligent assistive technology: findings from a qualitative study with professional stakeholders. BMC Medical Ethics 20(1). doi: 10.1186/s12910-019-0437-z

[229] Wayne K (2019) How Can Ethics Support Innovative Health Care for an Aging Population? Ethics & Behavior 29(3):227–253. doi: 10.1080/10508422.2018.1526087

[230] Weber K (2015) MEESTAR: ein Modell zur ethischen Evaluierung sozio-technischer Arrangements in der Pflege- und Gesundheitsversorgung. In: Technisierung des Alltags. Franz Steiner Verlag, 2015, Stuttgart

[231] Weber K (2017) Demografie, Technik, Ethik: Methoden der normativen Gestaltung technisch gestützter Pflege. Pflege & Gesellschaft

[232] Weßel M, Ellerich-Groppe N, Koppelin F, et al (2022) Gender and Age Stereotypes in Robotics for Eldercare: Ethical Implications of Stakeholder Perspectives from Technology Development, Industry, and Nursing. Science and engineering ethics 28(4):34. doi: 10.1007/s11948-022-00394-1

[233] Weßel M, Ellerich-Groppe N, Schweda M (2021) Gender Stereotyping of Robotic Systems in Eldercare: An Exploratory Analysis of Ethical Problems and Possible Solutions. International Journal of Social Robotics 15:1963–1976. doi: 10.1007/s12369-021-00854-x

[234] Wiczorek R, Bayles M, Rogers W (2020) Domestic Robots for Older Adults: Design Approaches and Recommendations. In: Woodcock A, Moody L, McDonagh D, et al (eds) Design of assistive technology for ageing populations. Intelligent Systems Reference Library, Springer, Cham, p 203–219

[235] Wiertz S (2020) Trusting Robots?: On the Concept of Trust and on Forms of Human Vulnerability. In: Haltaufderheide J, Hovemann J, Vollmann J (eds) Aging between Participation and Simulation: Ethical Dimensions of Socially Assistive Technologies in Elderly Care. De Gruyter, p 53–68

[236] Wirth L, Siebenmann J, Gasser A (2020) Erfahrungen aus dem Einsatz von Assistenzrobotern für Menschen im Alter. In: Buxbaum HJ (ed) Mensch-Roboter-Kollaboration. Mensch-Roboter-Kollaboration, Springer Gabler, Wiesbaden, p 257–279, doi: 10.1007/978-3-658-28307-0_17

[237] Wu YH, Fassert C, Rigaud AS (2012) Designing robots for the elderly: Appearance issue and beyond. Archives of Gerontology and Geriatrics 54(1):121–126. doi: 10.1016/j.archger.2011.02.003

[238] Wu YH, Wrobel J, Cornuet M, et al (2014) Acceptance of an assistive robot in older adults: a mixed-method study of human–robot interaction over a 1-month period in the Living Lab setting. Clinical Interventions in Aging p 801. doi: 10.2147/CIA.S56435

[239] van Wynsberghe A (2016) Designing Care Robots with Care. In: van Wynsberghe A (ed) Healthcare Robots: Ethics, Design and Implementation. Routledge, p 9–20, doi: 10.4324/9781315586397

[240] van Wynsberghe A (2016) What is a Care Robot? In: van Wynsberghe A (ed) Healthcare Robots: Ethics, Design and Implementation. Routledge, p 61–68

[241] Yasuhara Y, Tanioka R, Tanioka T, et al (2019) Ethico-Legal Issues With Humanoid Caring Robots and Older Adults in Japan. International Journal for Human Caring 23(2):141–148. doi: 10.20467/1091-5710.23.2.141

[242] Yew GCK (2021) Trust in and Ethical Design of Carebots: The Case for Ethics of Care. International Journal of Social Robotics 13(4):629–645. doi: 10.1007/s12369-020-00653-w

[243] Zardiashvili L, Fosch-Villaronga E (2020) “Oh, Dignity too?” Said the Robot: Human Dignity as the Basis for the Governance of Robotics. Minds and Machines 30(1):121–143. doi: 10.1007/s11023-019-09514-6

[244] Zhang Z, Zhang C, Li X (2022) The Ethical Governance for the Vulnerability of Care Robots: Interactive-Distance-Oriented Flexible Design. Sustainability 14(4):2303. doi: 10.3390/su14042303

[245] Zöllick J, Kuhlmey A, Nordheim J, et al (2020) Technik und Pflege - eine ambivalente Beziehung. Pflegezeitschrift 73(3):50–53. doi: 10.1007/s41906-019-0653-7

[246] Zöllick J, Kuhlmey A, Nordheim J, et al (2022) Robotik in der Pflege – Potenziale und Grenzen. Der Hautarzt 73(5):405–407. doi: 10.1007/s00105-022-04965-y

[247] Zöllick JC, Rössle S, Kluy L, et al (2022) Potenziale und Herausforderungen von sozialen Robotern für Beziehungen älterer Menschen: eine Bestandsaufnahme mittels ”rapid review”. Zeitschrift für Gerontologie und Geriatrie 55(4):298–304. doi: 10.1007/s00391-021-01932-5

[248] Zwick MM, Hampel J (2019) Cui bono? Zum Für und Wider von Robotik in der Pflege. TATuP - Zeitschrift für Technikfolgenabschätzung in Theorie und Praxis 28(2):52–57. doi: 10.14512/tatup.28.2.s52
