# Supplementary material for: Ethical aspects of the use of social robots in caring for older people – a systematic qualitative review
Source: Med Health Care Philos. 2026 Feb 5;29(1):209–24. doi: 10.1007/s11019-025-10313-3 (PMC12960314; doi:10.1007/s11019-025-10313-3)
Supplement: Supplementary file 3 — Online Resource 3 (PDF 159 kb) [file 11019_2025_10313_MOESM3_ESM.pdf]

Online Resource 3 to: Ethical Aspects of the Use of Social Robots in Elderly Care

A Systematic Qualitative Review

Marianne Leineweber[1], Clara Victoria Keusgen[1], Marc Bubeck[1], Robert Ranisch[1*], Joschka Haltaufderheide[1†], Corinna Klingler[1†]

[1]Juniorprofessorship for Medical Ethics with a focus on Digitization, Faculty for Health Sciences Brandenburg, University of Potsdam, Am Mühlenberg 9, Potsdam, 14476, Brandenburg, Germany.

*Corresponding author(s). E-mail(s): ranisch@uni-potsdam.de;
†Joschka Haltaufderheide and Corinna Klingler contributed equally as last authors.

Further information on extracted data

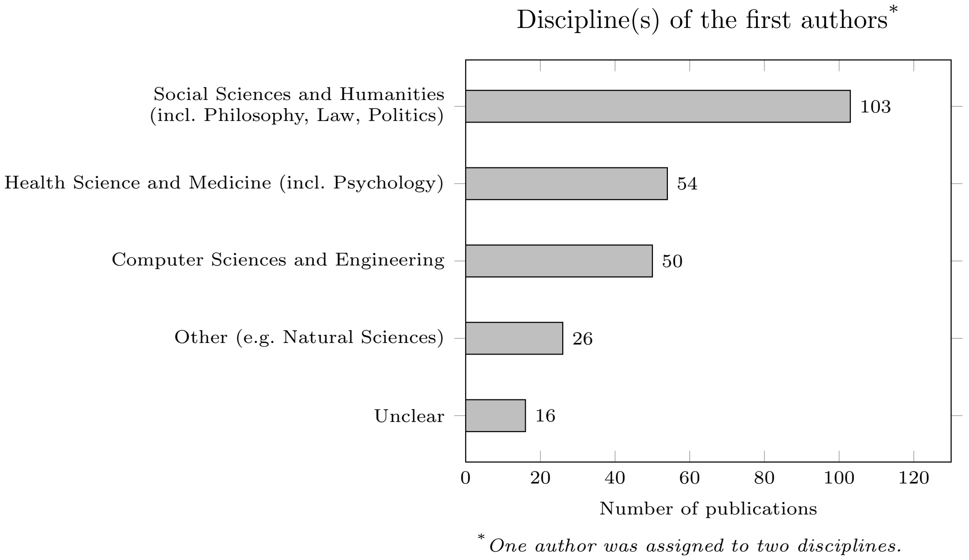

Fig. 1 Distribution of first authors' disciplines.

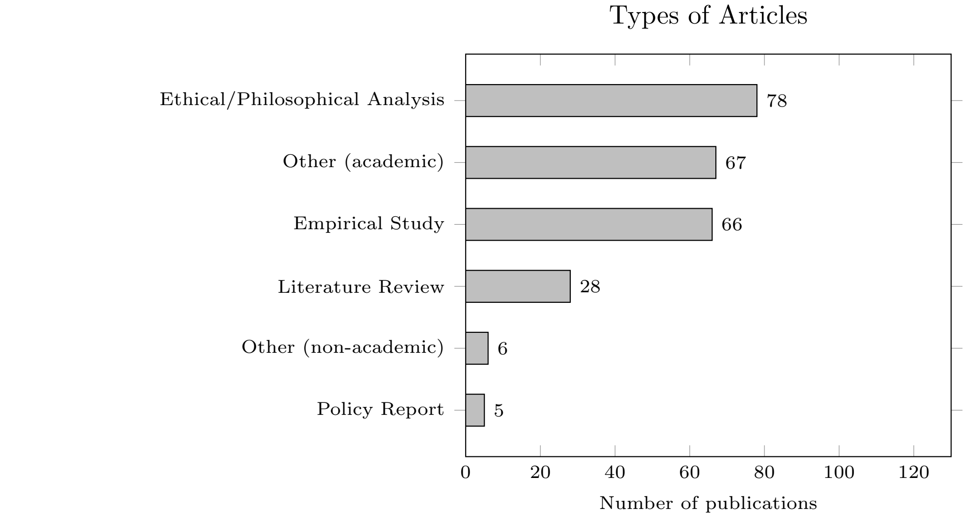

Fig. 2 Distribution of article types.

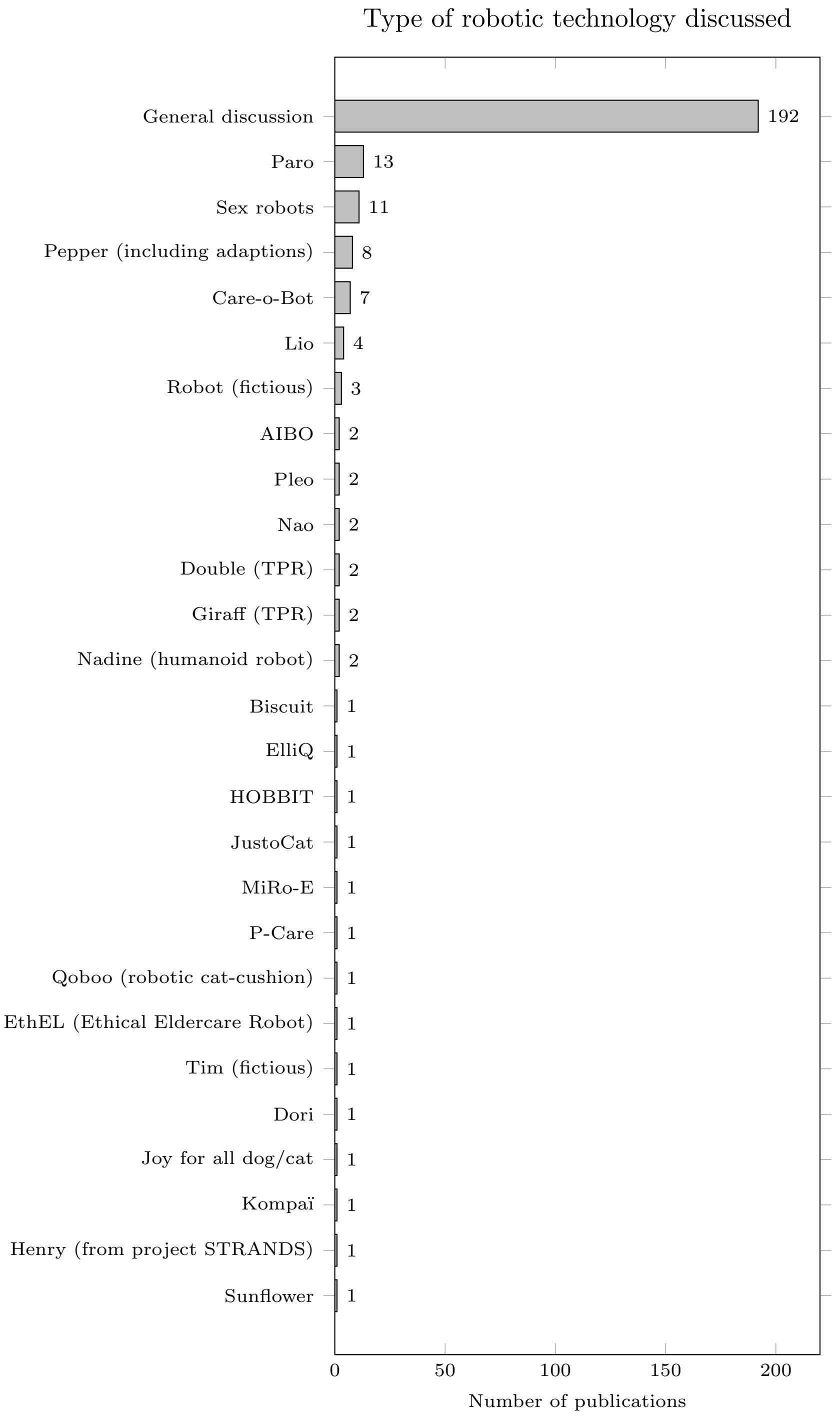

Fig. 3 Distribution of type of robotic technology discussed.

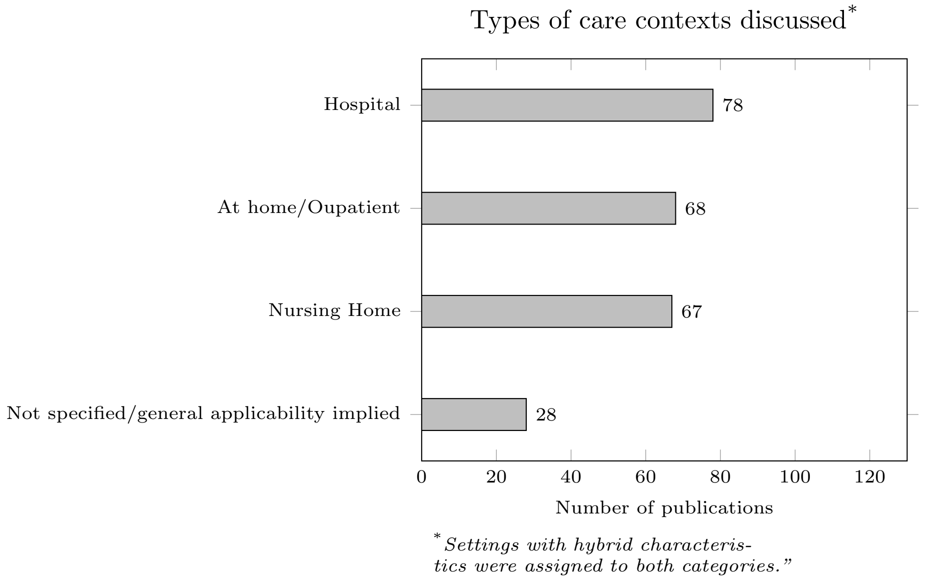

Fig. 4 Distribution of types of care context discussed.

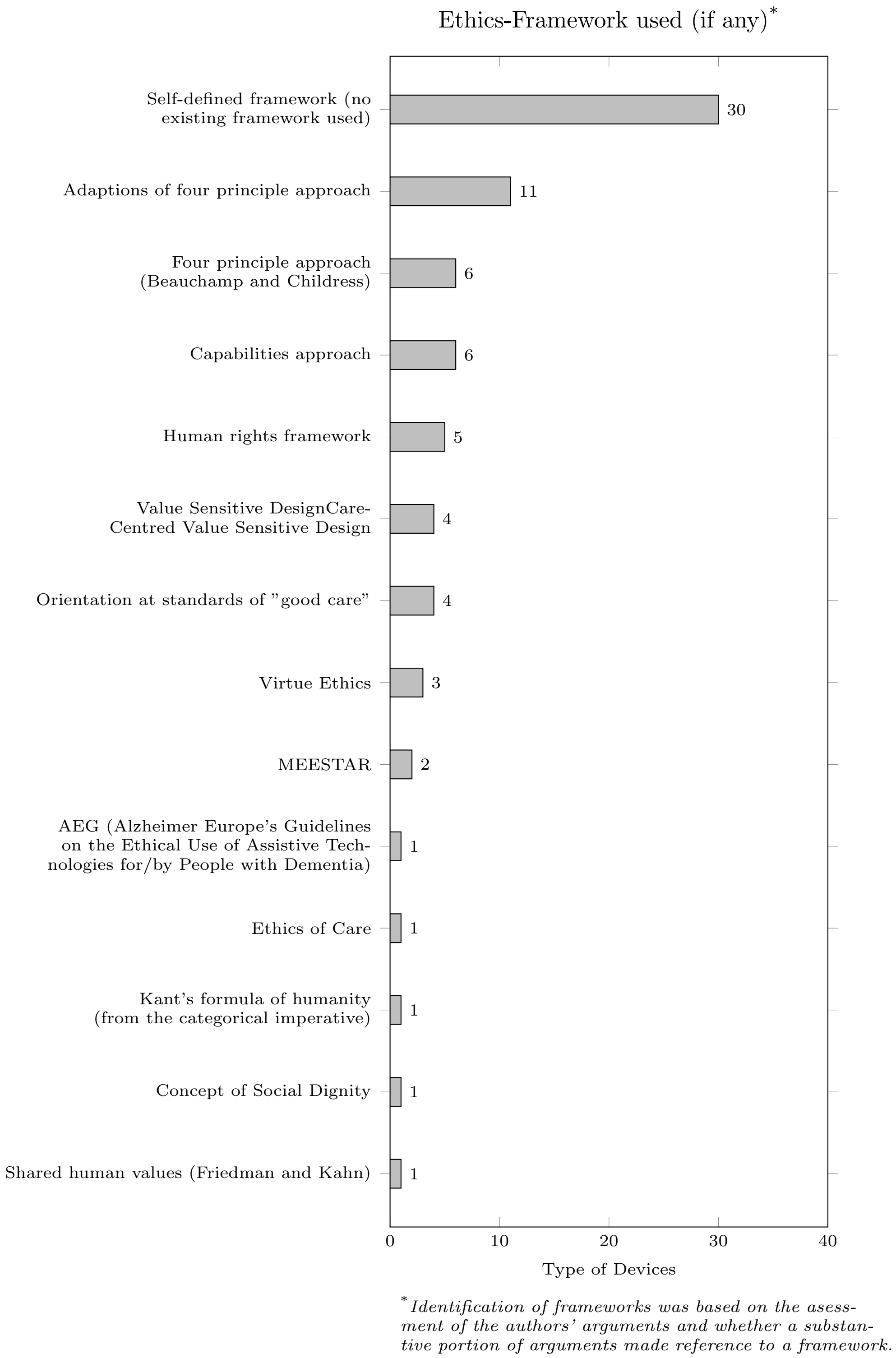

Fig. 5 Distribution of Ethics-Framework used (if any)
