# Supplementary material for: Ethical aspects of the use of social robots in caring for older people – a systematic qualitative review
Source: Med Health Care Philos. 2026 Feb 5;29(1):209–24. doi: 10.1007/s11019-025-10313-3 (PMC12960314; doi:10.1007/s11019-025-10313-3)
Supplement: Supplementary file 4 — Online Resource 4 (PDF 88 kb) [file 11019_2025_10313_MOESM4_ESM.pdf]

Online Resource 4 to: Ethical Aspects of the Use of Social Robots in Elderly Care

A Systematic Qualitative Review

Marianne Leineweber[1], Clara Victoria Keusgen[1], Marc Bubeck[1], Robert Ranisch[1*], Joschka Haltaufderheide[1†], Corinna Klingler[1†]

[1]Juniorprofessorship for Medical Ethics with a focus on Digitization, Faculty for Health Sciences Brandenburg, University of Potsdam, Am Mühlenberg 9, Potsdam, 14476, Brandenburg, Germany.

*Corresponding author(s). E-mail(s): ranisch@uni-potsdam.de;
†Joschka Haltaufderheide and Corinna Klingler contributed equally as last authors.

Search strings

CINAHL

((MH ”Robotics+”) OR (MH ”Assistive Technology”) OR TX robot* OR (TX social* AND TX assistiv*) OR (TX sozial* and TX assistiv*) OR (TX social* AND TX interactiv*) OR (TX sozial* AND interaktiv*)) AND ((MH ”Aged+”) OR (MH ”Geriatrics+”) OR (MH ”Dementia+”) OR (MH ”Dementia Patients”) OR (MH ”Nursing Home Patients”) OR TX elderl* OR TX Pflege OR TX Altenpflege OR TX old* OR TX aged OR TX senior* OR TX geriatr* OR TX dement* OR TX Demenz*) AND ((MH ”Ethics+”) OR (MH ”Ethics, Medical”) OR (MH ”Ethics, Nursing”) OR (MH ”Morale”) OR (MH ”Morals”) OR TX ethic* OR TX ethis* OR TX Ethik OR (TX moral* NOT TX ”Morales“))

TIB

(title:(Robot*) OR ”socially assistive” OR ”sozial assistiv” OR ”sozial assistive” OR ”socially interactive” OR ”sozial interaktiv” OR ”sozial interaktive” OR ”companion robot” OR ”companion robots” OR ”Begleitroboter” OR ”care robots” OR ”care robot” OR ”Pflegeroboter” OR ”service robot” OR ”service robots” OR ”Serviceroboter”) AND (Pflege OR Altenpflege OR alt* OR old* OR aged OR elderl* OR senior* OR geriatr* OR dement* OR Demenz*) AND (ethic* OR ethis* OR Ethik OR moral* NOT ”Morales”)

BELIT

(Robot* OR (social* AND assistiv*) OR (sozial* AND assistiv*) OR (social* AND interactiv*) OR (sozial* AND interaktiv*)) AND (Pflege OR Altenpflege OR alt* OR old* OR aged OR elderl* OR senior* OR geriatr* OR dement* OR Demenz*)
